# Water Triggers Hydrogen‐Bond‐Network Reshaping in the Glycoaldehyde Dimer

**DOI:** 10.1002/anie.201914888

**Published:** 2020-03-20

**Authors:** Cristóbal Pérez, Amanda L. Steber, Berhane Temelso, Zbigniew Kisiel, Melanie Schnell

**Affiliations:** ^1^ Deutsches Elektronen-Synchrotron DESY Notkestraße 85 22607 Hamburg Germany; ^2^ Christian-Albrechts-Universität zu Kiel Max-Eyth-Str. 1 24118 Kiel Germany; ^3^ Division of Information Technology College of Charleston Charleston SC 29424 USA; ^4^ Institute of Physics Polish Academy of Sciences 02-668 Warszawa Poland

**Keywords:** Chirality, Hydration, Hydrogen bonding, Rotational Spectroscopy, Self-aggregation

## Abstract

Carbohydrates are ubiquitous biomolecules in nature. The vast majority of their biomolecular activity takes place in aqueous environments. Molecular reactivity and functionality are, therefore, often strongly influenced by not only interactions with equivalent counterparts, but also with the surrounding water molecules. Glycoaldehyde (Gly) represents a prototypical system to identify the relevant interactions and the balance that governs them. Here we present a broadband rotational‐spectroscopy study on the stepwise hydration of the Gly dimer with up to three water molecules. We reveal the preferred hydrogen‐bond networks formed when water molecules sequentially bond to the sugar dimer. We observe that the dimer structure and the hydrogen‐bond networks at play remarkably change upon the addition of just a single water molecule to the dimer. Further addition of water molecules does not significantly alter the observed hydrogen‐bond topologies.

## Graphical abstract

**1‐2‐3, leave the structure be**: Broadband rotational spectroscopy reveals the structure and hydrogen‐bond networks of the glycoaldehyde dimer upon interaction with water. The structure of the isolated dimer changes upon addition of a single water molecule, while further addition of one or two water molecules does not significantly alter the hydrogen‐bond network observed for the monohydrate.

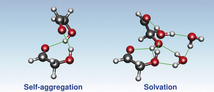

Carbohydrates belong to the most abundant and versatile classes of biomolecules. They are key players in a wide variety of biological functions such as cell‐energy‐storage units, tags of cellular identity in membranes, structural building blocks in plants, and as fundamental constituents of nucleotides in RNA and DNA.^1^ At a molecular level, their interactions with partner molecules as well as with the surrounding aqueous environment are controlled by a subtle interplay of intra‐ and intermolecular contacts.^2^ These range from stronger, more directional contacts such as hydrogen bonds (HB) to weaker, less directional dispersive interactions. Since these interactions are largely responsible for the molecular function and activity of the sugars, an in‐depth knowledge of these forces is mandatory in order to enlarge our understanding of such biological processes. Of particular interest is the competing, complementary trade‐off between self‐aggregation and the interactions with surrounding molecules of water. A well‐established approach to learn about these interactions is to generate and characterize small molecular self‐aggregates and/or their complexes with a few water molecules in the gas phase using supersonic jets.^3^ Under these isolated conditions, several monomer units or water molecules can be added to the structure of the cluster in a stepwise, controlled manner. The resulting molecular clusters can then be probed with different spectroscopic techniques to reveal their structural preferences and shed light on the preferred anchoring sites for both intra‐ and intermolecular interactions.^4^ Among the simple sugars, glycoaldehyde (Gly), HOCH_2_−CHO, has been the object of numerous experimental and theoretical studies.^5^, ^6^, ^7^, ^8^, ^9^, ^10^, ^11^ From a structural standpoint, Gly is often considered to be the simplest sugar (diose), as it is the smallest molecule containing both an aldehyde and a hydroxyl group (aldoses). Gly has been shown to be a key intermediate in the formation of larger sugars through the formose reaction,^5^ and it has even been discovered in the interstellar medium.^6^ Using rotational spectroscopy, the structure and HB networks of the monomer^7^ and the complex with one molecule of water^8^ were investigated. The structural preferences of the dimer^9^ and its complex with water^10^ were also studied using IR and IR‐VUV spectroscopies, respectively. More recently, Zinn et al.^11^ studied the self‐aggregation of Gly using rotational spectroscopy and provided accurate structural information of two qualitatively different Gly dimers in the gas phase.

In this Communication, we present the structure and HB networks at play for the mixed (Gly)_2_‐(H_2_O)_1–3_ complexes using the high resolution and sensitivity of chirped‐pulse Fourier‐transform microwave (CP‐FTMW) spectroscopy.^12^, ^13^ We observe that the Gly‐dimer structure drastically changes with respect to that previously reported^11^ upon the addition of only one water molecule. This new structure exhibits a remarkable HB rearrangement, which is then preserved upon addition of a second and third water monomer.

The experiments were performed with the CP‐FTMW COMPACT spectrometer operating in the 2–8 and 8–15 GHz configurations. Experimental details as well as its operation principle have been described elsewhere.^14^, ^15^ A brief description follows. A commercial Gly (2,5‐dihydroxy‐1,4‐dioxane, dimeric form of Gly) sample was placed in an internal reservoir and heated to 80 °C. Water was introduced by flowing neon over an external reservoir. The gaseous mixture was then supersonically expanded into a vacuum chamber, which results in a rovibrational molecular cooling to low effective temperatures in the single‐digit Kelvin region. The isolated, cold molecules were probed by microwave radiation to yield the rotational spectrum of the most abundant polar species present in the molecular expansion.

Two sets of experiments were performed using first a pure H_2_^16^O sample followed by a sample doped with H_2_^18^O in a 1:3 (H_2_^18^O/H_2_^16^O) ratio. The first spectrum was used to identify the water‐containing clusters, while the second one was used to extract structural information from the changes in the moments of inertia upon isotopic substitution. The previously observed Gly monomer and dimer were easily identified at 4000:1 and 800:1 signal‐to‐noise ratios (SNR), respectively. Further analysis revealed the presence of (Gly)_2_‐H_2_O, (Gly)_2_‐(H_2_O)_2_, and (Gly)_2_‐(H_2_O)_3_. Small regions of the rotational spectrum of (Gly)_2_‐(H_2_O)_2_ are showcased in Figure 1 to demonstrate the unambiguous identification of the clusters and the effect of isotopic substitution. The experimental rotational parameters of the parent species are shown in Table 1, and rotational transition frequencies are reported in Tables S1–S11 of the Supporting Information. An exhaustive comparison of the rotational parameters and relative stability of the observed clusters using both MP2/6‐311++G(d,p) and MP2/aug‐cc‐pVDZ (aVDZ) as well as density‐functional methods (B3LYP‐D3(BJ)/def2‐TZVP) are reported in Tables S20–S25 and Figures S5–S8. The structural analysis was first based on Kraitchman's equations^16^ to evaluate the *r*_s_ structure. This approach determines the magnitude of an isotopically labeled atom coordinate in the principal‐axis system. The choice of undetermined signs of such coordinates usually relies on comparisons with structures from quantum chemistry. Comparisons between the experimental oxygen‐atom positions and the theoretical structures obtained using the aVDZ basis set can be found in the Supporting Information, Table S13.

**Figure 1:**
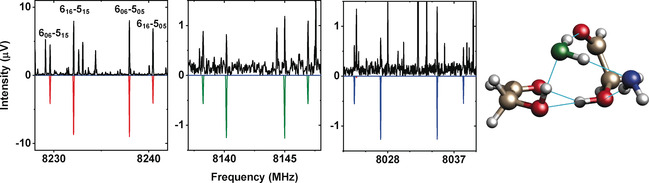
Sections of the rotational spectrum of (Gly)_2_‐(H_2_O)_2_, showing the effect of the single H_2_^18^O isotopic substitution in the spectrum of the cluster depending on the substituted water unit. The black trace is the experimental spectrum (4 million acquisitions), the colored traces are simulations at 1.5 K based on the fitted rotational parameters. The red trace simulates the spectrum of the species with two H_2_^16^O molecules. The blue and green traces show the spectra of single H_2_^18^O insertions according to the color code shown in the structure. The rotational levels involved in each transition are denoted using the standard asymmetric‐top notation, *J* ${}_{K{}_{a}K{}_{c}}$_,_ where *J* is the total rotational angular‐momentum quantum number and *K_a_*, *K_c_* represent the quantum numbers for the projection of the angular momentum onto the symmetry axis (*a*‐ or *c*‐axis) in the two limiting cases of prolate and oblate symmetric tops, respectively.

**Table 1:** Experimentally determined rotational parameters for the (Gly)_2_‐(H_2_O)_1–3_ complexes. *A*, *B*, and *C* are the rotational constants, Δ_*J*_, Δ_*JK*_, Δ_*K*_, δ_*J*_, and δ_*K*_ are the centrifugal‐distortion constants, σ is the deviation of the fit, *N* is the number of transitions in the fit, and κ is Ray's asymmetry parameter defined as (2*B*−*A*−*C*)/(*A*−*C*).

	(Gly)_2_‐H_2_O	(Gly)_2_‐(H_2_O)_2_	Gly)_2_‐(H_2_O)_3_
*A* (MHz)	1707.45161(31)	1160.01937(62)	789.9518(16)
*B* (MHz)	998.95996(22)	860.74300(26)	673.87562(84)
*C* (MHz)	862.07586(24)	659.75702(27)	507.12181(52)
			
Δ_*J*_ (kHz)	0.7619(50)	0.1264(30)	0.2670(77)
Δ_*JK*_ (kHz)	−2.0090(95)	1.172(20)	−0.597(34)
Δ_*K*_ (kHz)	4.6940(95)	−0.491(36)	–
δ_*J*_ (kHz)	0.1988(13)	–	0.0997(39)
δ_*K*_ (kHz)	0.602(24)	–	−0.113(29)
			
σ (kHz)	5.1	6.8	8.2
*N*	104	82	42
			
κ	−0.67	−0.19	0.18

A different, well‐established method is to perform a least‐squares fit^17^, ^18^ (*r_0_*, *r*_m_^(1)^, …) to the experimentally available moments of inertia of a theoretical structure, where some geometrical parameters are constrained. This method yields high‐quality experimental information that is used to determine both the structures and the interactions that hold a molecular system together. The relevant structural parameters to determine the intermolecular heavy‐atom backbone of the (Gly)_2_‐(H_2_O)_*n*_ clusters are the O⋅⋅⋅O distances, and these are shown in Figure 2, while the HB distances are reported in Figures S9–S12 for the sake of clarity. The experimental structures are compared to the previously observed (Gly)_2_.^11^ As shown in Figure 2 a, (Gly)_2_ exhibits *C*_2_ spatial symmetry with two equivalent intramolecular HBs between the hydroxy and the carbonyl groups. Upon the addition of one water molecule, a remarkable rearrangement occurs. The water monomer is inserted into the structure of the cluster in such a way that it establishes interactions with both Gly units simultaneously, acting as both a donor and an acceptor. To map out these interactions, noncovalent interaction (NCI) analysis^19^ was used, and the results are displayed in Figure 2. This method allows for an intuitive, three‐dimensional description of the interactions at play. This analysis confirms the existence of two strong, highly directional HBs between the two Gly monomers and the water molecule, as well as two additional, weaker HBs between the two Gly units that establish a bifurcated contact to best exploit the donor–acceptor character of the monomers with 2.43 Å and 1.98 Å. Moreover, the two sugar units are further stabilized by a weaker but non‐negligible interaction that spans throughout the carbon backbone of one of the monomers.

**Figure 2:**
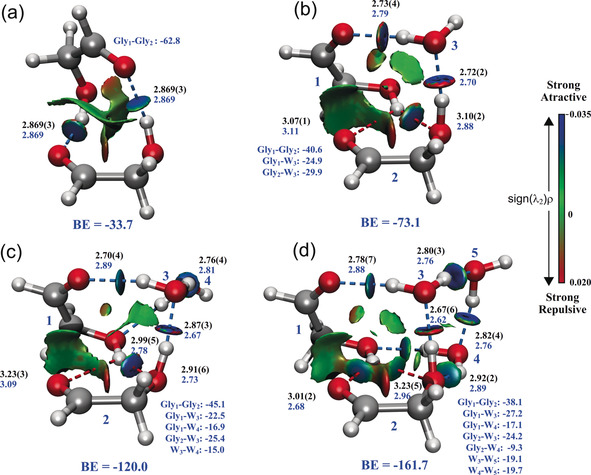
Experimental structures for a) (Gly)_2_, b) (Gly)_2_‐H_2_O, c) (Gly)_2_‐(H_2_O)_2_, and d) (Gly)_2_‐(H_2_O)_3_. The structure of (Gly)_2_ was taken from Zinn et al.^11^ (b–d) are the results of *r*_0_ least‐squares fits to the available moments of inertia for each cluster. The relevant experimental O⋅⋅⋅O distances (black) are compared to those predicted by theory with the MP2/aVDZ method (blue). The bifurcated HBs are highlighted by dotted red lines. All distances are in Å. The NCI plots map the location and strength of intermolecular interactions. Interactions range from attractive, strong HBs shown in blue to repulsive interactions shown in red based on the sign of (λ_2_)ρ. λ_2_ is the second eigenvalue of the electron‐density Hessian and ρ is the electron density. The two‐body stabilization energies for each pairwise interaction within each cluster and the total binding energies (BE) are also displayed in blue. Energies are shown in kJ mol^−1^ and were calculated using the MP2‐F12/VTZ‐F12//MP2/aVDZ method.

(Gly)_2_‐(H_2_O)_2_ displays clear similarities with the one‐water‐molecule cluster. The second water molecule (W4 in Figure 2 c) maximizes the interactions within the cluster, also exploiting its donor–acceptor character. It is linked to the dangling hydrogen of the first water molecule through an O_w_H⋅⋅⋅O_w_ HB (acting as an acceptor) and establishes an additional HB with one of the Gly hydroxy groups O_w_H⋅⋅⋅O_Gly_H (which fulfills its role as a donor). The two water molecules along with the two hydroxy groups create an additional eight‐membered HB ring that further stabilizes the structure and resembles the arrangement of the pure‐water tetramer.^20^ The addition of the second water unit does not noticeably modify the HB network created in the one‐water‐molecule cluster, and the O⋅⋅⋅O distances are only slightly modified to host the additional water monomer. The bifurcated HB between the two Gly monomers is preserved, with computed distances of 2.41 Å and 1.86 Å.

The stepwise addition of the third water monomer enabled the observation of the (Gly)_2_‐(H_2_O)_3_ complex shown in Figure 2 d. This cluster can be formed by the insertion of a water molecule (W4) between the two water monomers in the two‐water‐molecules complex. The structure is stabilized by the presence of two tri‐coordinated water molecules (W3 and W4) that take part in a less distorted water tetramer in which one of the corners is occupied by a hydroxy group of Gly. The two Gly units still exhibit a strong, bifurcated HB between them with theoretical distances of 2.05 Å and 2.17 Å. This interaction survives the complexation of three molecules and shows how the Gly–Gly interactions are of crucial importance even when the sugar is surrounded by a handful of water molecules. Additionally, cooperative effects are likely to be responsible for the stabilization of the observed HB networks. At a first glance, a cooperativity effect is not observed, however, further insight could be drawn by accounting for the local environment of a given HB. This leads to cooperative and anticooperative adjacent interactions that strengthen or weaken a particular HB and therefore affect the O⋅⋅⋅O distances.^21^

To gain further insight into the nature and magnitude of the molecular interactions at play, we performed a many‐body decomposition (MBD) of the interaction energy of each cluster.^22^ In MBD, the total interaction energy of a cluster is expanded as the sum of the one‐body deformation energy of each monomer as it distorts to form a stable cluster, and the binding energy of the cluster. The binding energy is the sum of all many‐body (two‐body, three‐body, four‐body, …) interactions between the monomers. This allows us to establish the main contributors to the interaction energy. The complete analysis is reported in Figures S9–S13 and Tables S26–S30 of the Supporting Information. The two‐body contributions of all pairwise interactions within each cluster are shown in Figure 2 and Tables S26–S30. Several interesting conclusions can be drawn. First, we observe that the one‐body deformation energy of the glycoaldehyde monomers in the clusters can be as large as 14.6 kJ mol^−1^ for (Gly)_2_, 25.8 kJ mol^−1^ for (Gly)_2_‐(H_2_O), 25.6 kJ mol^−1^ for (Gly)_2_‐(H_2_O)_2_, and 22.0 kJ mol^−1^ for (Gly)_2_‐(H_2_O)_3_, as reported in Tables S26–S30. The global minimum of the glycoaldehyde monomer (see Figure S14) in the gas phase has a *cis* configuration of *C_s_* symmetry stabilized by an intramolecular hydrogen bond between the hydroxyl group and the carbonyl group. As the monomers form clusters, they undergo a remarkable distortion from the *cis* configuration in their isolated gas‐phase form^7^ to a conformation in which the hydroxyl group rotates away from the carbonyl group by 60–70°. They also undergo torsion of up to 10° about the C−C axis relative to the *cis* configuration. We observe this effect for both the Gly dimer and the hydrated clusters. To further explore this, we calculated the barrier to this rotation about the C−O and C−C bonds by performing fully relaxed potential‐energy scans and obtained values of 20–25 kJ mol^−1^ and 21–33 kJ mol^−1^, respectively, as shown in Figures S15 and S16 of the Supporting Information. The observed Gly torsions optimize the HB interactions with the other Gly monomers and with the solvating water molecules. Higher‐energy forms of the constituent monomers have also been previously reported in trimers.^23^, ^24^

Related to this, the rotation of the hydroxy group generates non‐superimposable geometries that can be seen as transient enantiomers.^25^ This transient chirality is frozen when the cluster is formed, and it can be extracted from our observations. It is worth mentioning that in all observed clusters, the two Gly monomers are present in the same spatial orientation. They can, therefore, be seen as superimposable enantiomers that show the same handedness. This is a particularly relevant finding, as it indicates that self‐recognition and homochirality play an important role already in these small aggregates.

Second, we observe that the pairwise contacts are the main contributors to the stabilization of each cluster. Figure 2 shows the two‐body contributions between each pair of monomers, Gly–Gly, Gly–water and water–water. The largest contribution to the binding energy in all clusters comes from the interaction between the two sugar units. This can be attributed to the existence of the above‐mentioned bifurcated HB that persists in all hydrated clusters. Despite the relatively long experimental O–O distances (≈3 Å), the persistence of this kind of interaction constitutes a solid ground for cluster growth.

Lastly, this analysis allows us to study the effect of the stepwise addition of water from an energetic standpoint. As mentioned above, the addition of water appears to be sequential and builds on the already formed cluster. Therefore, it is of interest to look at features of the initial solvation unit, that is, consisting of Gly1, Gly2, and W3 (see Figure 2) in all three observed hydrated clusters. It is notable that the many‐body analysis shows remarkably consistent results for all three clusters as well as for one‐, two‐, and many‐body contributions (see Figure S17 and Table S31 in the Supporting Information for the full analysis). These results show that this solvation unit is not only structurally similar among clusters, but also presents comparable energetic features that make it an important building block and support the sequential formation of the observed water clusters. Preliminary calculations on (Gly)_3_ and (Gly)_3_‐H_2_O show that the same structural features observed for (Gly)_2_‐(H_2_O)_*n*_ clusters persist in the larger clusters.

In summary, the successful combination of broadband rotational spectroscopy and high‐level computational chemistry allowed for the identification and characterization of microsolvated structures of the smallest sugar dimer. The accurate structural information shows that upon complexation with water, the structure and HB networks of the isolated dimer undergo a drastic change even when only one water molecule is added to the cluster. Sequential further addition of water molecules essentially preserves the basic structure of the one‐water‐molecule aggregate. All structures exhibit the presence of a bifurcated HB between the two sugar units. Many‐body energy decomposition analysis found that this interaction is the main contributor to the binding energy of the cluster. Our results show that self‐aggregation is of crucial importance in the solvation of sugars and prevails over the formation of other HBs with the solvent molecules. In all observed clusters, the sugar monomers present the same chirality, which points to self‐recognition and to the prevalence of homochirality that can be preserved for ever larger clusters.

## Conflict of interest

The authors declare no conflict of interest.

## Supporting information

As a service to our authors and readers, this journal provides supporting information supplied by the authors. Such materials are peer reviewed and may be re‐organized for online delivery, but are not copy‐edited or typeset. Technical support issues arising from supporting information (other than missing files) should be addressed to the authors.

Supplementary
