# Supplementary material for: Water Triggers Hydrogen‐Bond‐Network Reshaping in the Glycoaldehyde Dimer
Source: Angew Chem Int Ed Engl. 2020 Mar 20;59(22):8401–5. doi: 10.1002/anie.201914888 (PMC7318665; doi:10.1002/anie.201914888)

Angewandte Chemie
Eine Zeitschrift der Gesellschaft Deutscher Chemiker

Supporting Information

# Water Triggers Hydrogen-Bond-Network Reshaping in the Glycoaldehyde Dimer

*Cristóbal Pérez,* Amanda L. Steber, Berhane Temelso, Zbigniew Kisiel, and Melanie Schnell**

anie_201914888_sm_miscellaneous_information.pdf

# Supporting information for: Water Triggers Hydrogen Bond Network reshaping in the Glycoaldehyde Dimer

Cristóbal Pérez,[*,†] Amanda L. Steber,[†,‡] Berhane Temelso,[¶] Zbigniew Kisiel,[§] and Melanie Schnell[*,†,‡]

†*Deutsches Elektronen-Synchrotron DESY, Notkestraße 85, D-22607 Hamburg, Germany*

‡*Christian-Albrechts-Universität zu Kiel, Institute of Physical Chemistry, Max-Eyth-Straße 1, D-24118 Kiel, Germany*

¶*Division of Information Technology, College of Charleston, Charleston, SC 29403, USA*

§*Institute of Physics, Polish Academy of Sciences, 02-668 Warszawa, Poland*

E-mail: cristobal.perez@desy.de; melanie.schnell@desy.de

# Contents

**List of Figures** **S3**

**List of Tables** **S5**

**1 Experimental Results** **S9**

1.1 Rotational Constants and Observed Frequencies . . . . . . . . . . . . . . . S9

1.2 Structural Analysis . . . . . . . . . . . . . . . . . . . . . . . . . . . . . . . S22

**2 Computational Methodology** **S34**

2.1 Structure Determination . . . . . . . . . . . . . . . . . . . . . . . . . . . . S34

2.1.1 Genetic Algorithm Search on Semi-empirical PES . . . . . . . . . S34

2.1.2 Refinements using *ab initio* Methods . . . . . . . . . . . . . . . . S34

2.2 Rotational Constants, Principal Dipole Moments and Energies . . . . . . S36

2.2.1 Rotational Constants, Principal Dipole Moments and Relative Energies . . . . . . . . . . . . . . . . . . . . . . . . . . . . . . S36

2.2.2 Binding Energies . . . . . . . . . . . . . . . . . . . . . . . . . . . . S37

2.3 Comparison of QM Methods . . . . . . . . . . . . . . . . . . . . . . . . . S38

2.3.1 $(Gly)_2$ . . . . . . . . . . . . . . . . . . . . . . . . . . . . . . . . . . S40

2.3.2 $(Gly)_2W_1$ . . . . . . . . . . . . . . . . . . . . . . . . . . . . . . . S41

2.3.3 $(Gly)_2W_2$ . . . . . . . . . . . . . . . . . . . . . . . . . . . . . . . S42

2.3.4 $(Gly)_2W_3$ . . . . . . . . . . . . . . . . . . . . . . . . . . . . . . . S43

2.3.5 Takeaways . . . . . . . . . . . . . . . . . . . . . . . . . . . . . . . . S44

2.4 Many-Body Expansion of Cluster Interaction Energies . . . . . . . . . . . S46

2.4.1 Definition of Many-Body Expansion . . . . . . . . . . . . . . . . . S46

2.4.2 $(Gly)_2$ . . . . . . . . . . . . . . . . . . . . . . . . . . . . . . . . . . S48

2.4.3 $(Gly)_2W_1$ . . . . . . . . . . . . . . . . . . . . . . . . . . . . . . . S49

2.4.4 $(Gly)_2W_2$ . . . . . . . . . . . . . . . . . . . . . . . . . . . . . . . S51

2.4.5 $(Gly)_2W_3$ . . . . . . . . . . . . . . . . . . . . . . . . . . . . . . . S53

2.4.6 Comparison of $(Gly)_2$ and $(Gly)_1W_1$ . . . . . . . . . . . . . . . . . S55

2.4.7 Takeaways from MBE Analysis . . . . . . . . . . . . . . . . . . . . . S56

2.5 Torsional Potentials of Glycoaldhyde . . . . . . . . . . . . . . . . . . . . . . S57

2.5.1 Rotation of -O-H Group about -C-O- Axis . . . . . . . . . . . . . . S57

2.5.2 Torsion about the C-C bond . . . . . . . . . . . . . . . . . . . . . . S59

2.6 Effects of Adding Water Molecules Incrementally . . . . . . . . . . . . . . S60

2.7 Homochirality of Glycoaldehyde Monomers in $Gly_nW_m$ Clusters . . . . S62

2.8 Cartesian Coordinates . . . . . . . . . . . . . . . . . . . . . . . . . . . . . S64

2.8.1 Glycoaldehyde and Water Monomers . . . . . . . . . . . . . . . . . S64

2.8.2 $(Gly)_2$ . . . . . . . . . . . . . . . . . . . . . . . . . . . . . . . . . S66

2.8.3 $(Gly)_2W_1$ . . . . . . . . . . . . . . . . . . . . . . . . . . . . . . . S68

2.8.4 $(Gly)_2W_2$ . . . . . . . . . . . . . . . . . . . . . . . . . . . . . . . S71

2.8.5 $(Gly)_2W_3$ . . . . . . . . . . . . . . . . . . . . . . . . . . . . . . . S73

**References** **S76**

# List of Figures

S1 Preferred partial $r_0$ geometries determined for the $Gly_2$-$(H_2O)_n$ clusters. The experimental O-O distances are shown in Angstrom. . . . . . . . . . S24

S2 Atom numbering used in the least-squares fit of the $Gly_2$-$H_2O$ structure. S25

S3 Atom numbering used in the least-squares fit of the $Gly_2$-$(H_2O)_2$ structure. S28

S4 Atom numbering used in the least-squares fit of the $Gly_2$-$(H_2O)_3$ structure. S31

S5 Comparison of the lowest energy isomers of $(Gly)_2$ computed using different QM methods. See Table S22 below. . . . . . . . . . . . . . . . . . S40

S6 Comparison of the lowest energy isomers of $(Gly)_2W_1$ computed using different QM methods. See Table S23 below. . . . . . . . . . . . . . . . . . S41

S7 Comparison of the lowest energy isomers of $(Gly)_2W_2$ computed using different QM methods. See Table S24 below. . . . . . . . . . . . . . . . . . S42

S8 Comparison of the lowest energy isomers of $(Gly)_2W_3$ computed using different QM methods. See Table S25 below. . . . . . . . . . . . . . . . . . S43

S9 The two MP2-F12/VTZ-F12//MP2/aVDZ lowest energy isomers of $(Gly)_2$. The numbers in green correspond to the monomer/fragment labels in the table below. . . . . . . . . . . . . . . . . . . . . . . . . . . . . . . . S48

S10 The three MP2-F12/VTZ-F12//MP2/aVDZ lowest energy isomers of $(Gly)_2(W)_1$. The numbers in green correspond to the monomer/fragment labels in the table below. . . . . . . . . . . . . . . . . . . . . . . . . . . . . . S49

S11 The three MP2-F12/VTZ-F12//MP2/aVDZ lowest energy isomers of $(Gly)_2(W)_2$. The numbers in green correspond to the monomer/fragment labels in the table below. . . . . . . . . . . . . . . . . . . . . . . . . . . . . . S51

S12 The three MP2-F12/VTZ-F12//MP2/aVDZ lowest energy isomers of $(Gly)_2(W)_3$. The numbers in green correspond to the monomer/fragment labels in the table below. . . . . . . . . . . . . . . . . . . . . . . . . . . . . . S53

S13 Comparison of $(Gly)_2$ and $(Gly)_1W_1$'s MP2-F12/VTZ-F12//MP2/aVDZ interaction energies. The numbers in green correspond to the monomer/fragment labels in the table below. . . . . . . . . . . . . . . . . . . . . . . . . . . . . . S55

S14 The MP2/aVDZ geometry of glycoaldehyde. The torsional potentials of the H4-O1 are about the O1-C1 axis, and O2-C2 about the C2-C1 axis are plotted below. . . . . . . . . . . . . . . . . . . . . . . . . . . . . . . . S57

S15 The MP2/aVDZ and MP2-F12/VTZ-F12//MP2/aVDZ relaxed potential energy scan of the O-H torsion about the adjacent C-O axis of glycoaldehyde (H4-O1-C1-C2 in Figure S14). The barrier to free rotation of the hydroxyl group is 20 - 25 kJ $mol^{-1}$. . . . . . . . . . . . . . . . . . . S58

S16 The MP2/aVDZ relaxed potential energy scan of glycoaldehyde about the central C-C bond. The relevant dihedral angle here is O2-C2-C1-O1 in Figure S14. The barrier to rotation about C-C axis is 21 - 33 kJ $mol^{-1}$. S59

S17 The global minima of $(Gly)_2(W)_{1-3}$ superimposed on The global minimum of $(Gly)_2(W)_1$ is preserved in $(Gly)_2(W)_2$ and $(Gly)_2(W)_3$. The second water molecule of $Gly)_2(W)_2$ and the second and third water molecules of $Gly)_2(W)_3$ are removed for clarity. The numbers in green correspond to the monomer/fragment labels in the Table S31. . . . . . . S60
S18 Experimentally observed structures of $(Gly)_2W_{m=0-3}$ and MP2/aVDZ calculated structures of $(Gly)_3W_{m=0-1}$. . . . . . . . . . . . . . . . . . . . . . S63
S19 The structures of the low energy $(Gly)_2W_{n=0-3}$ isomers. See the tables below for their Cartesian coordinates. . . . . . . . . . . . . . . . . . . . . . S64

# List of Tables

S1 Experimentally determined rotational parameters for the $(Gly)_2$-$(H_2O)$ and singly $H_2^{18}O$ substituted complexes. A, B and C are the rotational constants. $\Delta_J$, $\Delta_{JK}$, $\Delta_K$, $\delta_J$, $\delta_K$ are the centrifugal distortion constants. $\sigma$ is the deviation of the fit. $N$ is the number of transitions in the fit. . . . . S9
S2 Measured rotational transitions (Obs) for the $(Gly)_2$-$(H_2O)$ complex, and the residuals (Obs-Calc) in MHz. . . . . . . . . . . . . . . . . . . . . . . . . S10
S3 Measured rotational transitions (Obs) for the $(Gly)_2$-$(H_2^{18}O)$ complex, and the residuals (Obs-Calc) in MHz. . . . . . . . . . . . . . . . . . . . . . S11
S4 Experimentally determined rotational parameters for the $(Gly)_2$-$(H_2O)_2$ and singly $H_2{}^{18}O$ substituted complexes. A, B and C are the rotational constants. $\Delta_J$, $\Delta_{JK}$, $\Delta_K$, $\delta_J$, $\delta_K$ are the centrifugal distortion constants. $\sigma$ is the deviation of the fit. $N$ is the number of transitions in the fit. . . . . S13
S5 Measured rotational transitions (Obs) for the $(Gly)_2$-$(H_2O)_2$ complex, and the residuals (Obs-Calc) in MHz. . . . . . . . . . . . . . . . . . . . . . S13
S6 Measured rotational transitions (Obs) for the $(Gly)_2$-$(H_2{}^{18}O1)(H_2{}^{16}O2)$ complex, and the residuals (Obs-Calc) in MHz. . . . . . . . . . . . . . . . S16

S7 Measured rotational transitions (Obs) for the $(Gly)_2$-$(H_2{}^{16}O1)(H_2{}^{18}O2)$ complex, and the residuals (Obs-Calc) in MHz. . . . . . . . . . . . . . . . S17
S8 Experimentally determined rotational parameters for the $(Gly)_2$-$(H_2O)_3$ and singly $H_2{}^{18}O$ substituted complexes. A, B and C are the rotational constants. $\Delta_J$, $\Delta_{JK}$, $\Delta_K$, $\delta_J$, $\delta_K$ are the centrifugal distortion constants. $\sigma$ is the deviation of the fit. $N$ is the number of transitions in the fit. Parameters in square brackets were fixed at the parent species values. . . S18
S9 Measured rotational transitions (Obs) for the $(Gly)_2$-$(H_2O)_3$ complex, and the residuals (Obs-Calc) in MHz. . . . . . . . . . . . . . . . . . . . . . . S18
S10 Measured rotational transitions (Obs) for the $(Gly)_2$-$(H_2{}^{18}O1)(H_2{}^{16}O2)(H_2{}^{16}O3)$ complex, and the residuals (Obs-Calc) in MHz. . . . . . . . . . . . . . . . S20
S11 Measured rotational transitions (Obs) for the $(Gly)_2$-$(H_2{}^{16}O1)(H_2{}^{18}O2)(H_2{}^{16}O3)$ complex, and the residuals (Obs-Calc) in MHz. . . . . . . . . . . . . . . . S20
S12 Measured rotational transitions (Obs) for the $(Gly)_2$-$(H_2{}^{16}O1)(H_2{}^{16}O2)(H_2{}^{18}O3)$ complex, and the residuals (Obs-Calc) in MHz. . . . . . . . . . . . . . . . S21
S13 Comparison of the values of principal coordinates (Å) for the oxygen atoms in the complexed water molecules in $Gly_2$-$(H_2O)_n$ clusters. . . . . S23
S14 The abbreviated results of fitting the partial $r_0$ geometry of the $Gly_2$-$H_2O$ cluster with the STRFIT program. . . . . . . . . . . . . . . . . . . . . . . S25
S15 The abbreviated results of fitting the partial $r_0$ geometry of the $Gly_2$-$(H_2O)_2$ cluster with the STRFIT program. . . . . . . . . . . . . . . . . . . S28
S16 The abbreviated results of fitting the partial $r_0$ geometry of the $Gly_2$-$(H_2O)_3$ cluster with the STRFIT program. . . . . . . . . . . . . . . . . . . S31
S17 Details of GA runs for $(Gly)_2W_n$, where $n = 0 - 3$ . . . . . . . . . . . . . S34
S18 The MP2/aVDZ rotational constants, principal dipole moments and MP2-F12/VTZ-F12//MP2/aVDZ relative energies of low energy isomers of $(Gly)_2W_n$, where $n = 0 - 3$ . . . . . . . . . . . . . . . . . . . . . . S36
S19 MP2-F12/VTZ-F12//MP2/aVDZ Binding Energies (ΔE, ΔG) of the low energy isomers of $(Gly)_2W_n$, where $n = 0 - 3$ . . . . . . . . . . . . . . . . S37

S20 The rotational constants (A, B, C in MHz) of the two low energy isomers of $(Gly)_2$ calculated using different DFT methods and basis sets as implemented in different packages. . . . . . . . . . . . . . . . . . . . . . . . S38
S21 The rotational constants (A, B, C in MHz) of the two low energy isomers of $(Gly)_2$ calculated using MP2 and different basis sets as implemented in different packages. . . . . . . . . . . . . . . . . . . . . . . . . . . . . S39
S22 The rotational constants (A, B, C in MHz) and relative energies ($\Delta\Delta E_e$ in kcal mol$^{-1}$) of the low energy isomers of $(Gly)_2$ calculated using MP2-F12/VTZ-F12//MP2/aVDZ, MP2/6-311++G(d,p) and B3LYP-D3BJ/def2-TZVP . . . . . . . . . . . . . . . . . . . . . . . . . . . . . . . . . . S40
S23 The rotational constants (A, B, C in MHz) and relative energies ($\Delta\Delta E_e$ in kcal mol$^{-1}$) of the low energy isomers of $(Gly)_2W_1$ calculated using MP2-F12/VTZ-F12//MP2/aVDZ, MP2/6-311++G(d,p) and B3LYP-D3BJ/def2-TZVP . . . . . . . . . . . . . . . . . . . . . . . . . . . . . . . . . . S42
S24 The rotational constants (A, B, C in MHz) and relative energies ($\Delta\Delta E_e$ in kcal mol$^{-1}$) of the low energy isomers of $(Gly)_2W_2$ calculated using MP2-F12/VTZ-F12//MP2/aVDZ, MP2/6-311++G(d,p) and B3LYP-D3BJ/def2-TZVP . . . . . . . . . . . . . . . . . . . . . . . . . . . . . . . . . . S43
S25 The rotational constants (A, B, C in MHz) and relative energies ($\Delta\Delta E_e$ in kcal mol$^{-1}$) of the low energy isomers of $(Gly)_2W_3$ calculated using MP2-F12/VTZ-F12//MP2/aVDZ, MP2/6-311++G(d,p) and B3LYP-D3BJ/def2-TZVP . . . . . . . . . . . . . . . . . . . . . . . . . . . . . . . . . . S44
S26 Many-body decomposition of the MP2-F12/VTZ-F12//MP2/aVDZ binding energy of two $(Gly)_2(W)_0$ isomers in kJ mol$^{-1}$ . . . . . . . . . . . . . S48
S27 Many-body decomposition of the MP2-F12/VTZ-F12//MP2/aVDZ binding energy of three $(Gly)_2(W)_1$ isomers in kJ mol$^{-1}$ . . . . . . . . . . . . S50
S28 Many-body decomposition of the MP2-F12/VTZ-F12//MP2/aVDZ binding energy of two $(Gly)_2(W)_2$ isomers in kJ mol$^{-1}$ . . . . . . . . . . . . . S52

S29 Many-body decomposition of the MP2-F12/VTZ-F12//MP2/aVDZ binding energy of three $(Gly)_2(W)_3$ isomers in kJ $mol^{-1}$ . . . . . . . . . . . . S54
S30 Many-body decomposition of the MP2-F12/VTZ-F12//MP2/aVDZ binding energy of two $(Gly)_2$ and $(Gly)_1(W)_1$ isomers in kJ $mol^{-1}$ . . . . . . . S55
S31 Many-body decomposition of the MP2-F12/VTZ-F12//MP2/aVDZ interaction energy of the global minima of $(Gly)_2(W)_{1-3}$ in kJ $mol^{-1}$. Only the components corresponding to the $(Gly)_2(W)_1$ unit in $(Gly)_2(W)_{1-3}$ shown in Figure S17 are reported here. . . . . . . . . . . . . . . . . . . . . . S61
S32 MP2/aVDZ optimized Cartesian coordinates[a] of glycoaldehyde (Gly) and water monomers . . . . . . . . . . . . . . . . . . . . . . . . . . S65
S33 MP2/aVDZ optimized Cartesian coordinates[a] of $(Gly)_2W_0$-I . . . . . . . S66
S34 MP2/aVDZ optimized Cartesian coordinates[a] of $(Gly)_2W_0$-II . . . . . . . S67
S35 MP2/aVDZ optimized Cartesian coordinates[a] of $(Gly)_2W_1$-I . . . . . . . S68
S36 MP2/aVDZ optimized Cartesian coordinates[a] of $(Gly)_2W_1$-II . . . . . . . S69
S37 MP2/aVDZ optimized Cartesian coordinates[a] of $(Gly)_2W_1$-III . . . . . . S70
S38 MP2/aVDZ optimized Cartesian coordinates[a] of $(Gly)_2W_2$-I . . . . . . . S71
S39 MP2/aVDZ optimized Cartesian coordinates[a] of $(Gly)_2W_2$-II . . . . . . . S72
S40 MP2/aVDZ optimized Cartesian coordinates[a] of $(Gly)_2W_3$-I . . . . . . . S73
S41 MP2/aVDZ optimized Cartesian coordinates[a] of $(Gly)_2W_3$-II . . . . . . . S74
S42 MP2/aVDZ optimized Cartesian coordinates[a] of $(Gly)_2W_3$-III . . . . . . S75

# 1 Experimental Results

The rotational spectra were analyzed using JB95,[S1] AABS[S2] and Pgopher[S3] program suites. In all cases, we use a Watson's Hamiltonian in the A-reduction and the $I^r$ representation.

Note: We would like to report a correction to "High-resolution rotational spectroscopy study of the smallest sugar dimer: Interplay of hydrogen bond in the glycoaldehyde dimer" *Angew. Chem.* **2016**, 128, 60079-6084. doi: 10.1002/anie.201511077. In that study, the experimentally determined structure of the glycoaldehyde dimer is reported and compared to quantum chemical calculations. An error occured in Figure 4 for the intermolecular distances C1-C2′/C2-C1′ and C1-C1′/C2-C2′ of isomer 1. The correct values are 4.052(4) and 4.451(1), respectively.

## 1.1 Rotational Constants and Observed Frequencies

Table S1: Experimentally determined rotational parameters for the (Gly)$_2$-($H_2O$) and singly $H_2^{18}O$ substituted complexes. A, B and C are the rotational constants. $\Delta_J$, $\Delta_{JK}$, $\Delta_K$, $\delta_J$, $\delta_K$ are the centrifugal distortion constants. $\sigma$ is the deviation of the fit. $N$ is the number of transitions in the fit.

| | (Gly)$_2$-$H_2O$ | (Gly)$_2$-($H_2^{18}O$) |
|---|---|---|
| A (MHz) | 1707.45161(31) | 1645.08086(63) |
| B (MHz) | 998.95996(22) | 998.17133(34) |
| C (MHz) | 862.07586(24) | 846.11989(39) |
| $\Delta_J$ (kHz) | 0.7619(50) | 0.7507(68) |
| $\Delta_{JK}$ (kHz) | -2.0090(95) | -1.996(41) |
| $\Delta_K$ (kHz) | 4.6940(95) | 4.750(58) |
| $\delta_J$ (kHz) | 0.1988(13) | 0.2113(23) |
| $\delta_K$ (kHz) | 0.602(24) | - |
| $\sigma$ (kHz) | 5.1 | 4.9 |
| $N$ | 104 | 52 |

Table S2: Measured rotational transitions (Obs) for the $(Gly)_2$-$(H_2O)$ complex, and the residuals (Obs-Calc) in MHz.

| $J'$ | $K_a'$ | $K_c'$ | $J''$ | $K_a''$ | $K_c''$ | Obs | (Obs-Calc) |
|---|---|---|---|---|---|---|---|
| 6 | 2 | 4 | 6 | 1 | 5 | 2072.0915 | 0.0062 |
| 1 | 1 | 0 | 0 | 0 | 0 | 2706.4064 | 0.0006 |
| 2 | 0 | 2 | 1 | 1 | 1 | 2995.5731 | 0.0001 |
| 2 | 2 | 1 | 2 | 1 | 2 | 2536.0801 | -0.0021 |
| 2 | 2 | 0 | 2 | 1 | 2 | 2554.0649 | 0.0013 |
| 3 | 2 | 2 | 3 | 1 | 3 | 2752.0036 | -0.0001 |
| 3 | 2 | 1 | 3 | 1 | 3 | 2839.9328 | -0.0019 |
| 2 | 0 | 2 | 1 | 1 | 0 | 2858.6963 | 0.0033 |
| 4 | 2 | 3 | 4 | 1 | 4 | 3043.6315 | -0.0011 |
| 4 | 2 | 2 | 4 | 1 | 4 | 3295.2108 | -0.0003 |
| 5 | 2 | 4 | 5 | 1 | 5 | 3410.6197 | 0.0091 |
| 5 | 3 | 2 | 5 | 2 | 3 | 3525.9558 | 0.0079 |
| 2 | 1 | 2 | 1 | 1 | 1 | 3585.1777 | -0.0023 |
| 7 | 1 | 6 | 7 | 0 | 7 | 3614.4328 | -0.0015 |
| 4 | 3 | 2 | 4 | 2 | 2 | 3689.5234 | 0.0054 |
| 4 | 3 | 1 | 4 | 2 | 2 | 3699.8543 | -0.0001 |
| 2 | 0 | 2 | 1 | 0 | 1 | 3704.0673 | 0.0013 |
| 3 | 3 | 1 | 3 | 2 | 1 | 3807.1373 | 0.0001 |
| 3 | 3 | 0 | 3 | 2 | 1 | 3808.6316 | 0.0026 |
| 2 | 1 | 1 | 1 | 1 | 0 | 3858.9340 | 0.0033 |
| 3 | 3 | 1 | 3 | 2 | 2 | 3895.0677 | -0.0006 |
| 3 | 3 | 0 | 3 | 2 | 2 | 3896.5594 | -0.0004 |
| 4 | 3 | 2 | 4 | 2 | 3 | 3941.0959 | -0.0005 |
| 4 | 3 | 1 | 4 | 2 | 3 | 3951.4320 | -0.001 |
| 5 | 3 | 3 | 5 | 2 | 4 | 4028.7881 | 0.0014 |
| 5 | 3 | 2 | 5 | 2 | 4 | 4069.3134 | 0.0046 |
| 10 | 4 | 7 | 10 | 3 | 7 | 4102.3933 | -0.0051 |
| 6 | 3 | 3 | 6 | 2 | 5 | 4288.8591 | 0.0001 |
| 2 | 1 | 2 | 1 | 0 | 1 | 4293.6744 | 0.0014 |
| 4 | 1 | 4 | 3 | 2 | 1 | 4298.4078 | -0.0013 |
| 4 | 1 | 4 | 3 | 2 | 2 | 4386.3349 | -0.0051 |
| 3 | 0 | 3 | 2 | 1 | 1 | 4512.8420 | 0.0041 |
| 2 | 1 | 1 | 1 | 0 | 1 | 4704.3053 | 0.0015 |
| 3 | 0 | 3 | 2 | 1 | 2 | 4923.4674 | -0.0011 |
| 7 | 4 | 4 | 7 | 3 | 4 | 5135.1166 | 0.0087 |
| 9 | 7 | 3 | 8 | 8 | 0 | 5226.9092 | 0.0015 |
| 6 | 4 | 3 | 6 | 3 | 3 | 5281.9340 | 0.0097 |
| 6 | 4 | 2 | 6 | 3 | 3 | 5286.4386 | -0.0105 |
| 5 | 2 | 4 | 4 | 3 | 2 | 5323.9021 | -0.0041 |
| 5 | 4 | 2 | 5 | 3 | 2 | 5361.3251 | 0.0012 |
| 5 | 4 | 1 | 5 | 3 | 2 | 5362.2423 | 0.0027 |
| 3 | 1 | 3 | 2 | 1 | 2 | 5367.1515 | -0.0003 |
| 6 | 4 | 3 | 6 | 3 | 4 | 5399.1875 | 0.001 |
| 4 | 4 | 0 | 4 | 3 | 1 | 5399.3133 | 0.0039 |
| 5 | 4 | 2 | 5 | 3 | 3 | 5401.8493 | 0.0033 |
| 6 | 4 | 2 | 6 | 3 | 4 | 5403.7111 | -0.0001 |
| 4 | 4 | 1 | 4 | 3 | 2 | 5409.5434 | 0.0000 |
| 4 | 4 | 0 | 4 | 3 | 2 | 5409.6482 | 0.0023 |
| 3 | 0 | 3 | 2 | 0 | 2 | 5513.0726 | -0.0028 |
| 3 | 2 | 2 | 2 | 2 | 1 | 5583.0711 | -0.0023 |
| 3 | 2 | 1 | 2 | 2 | 0 | 5653.0243 | 0.0011 |
| 4 | 1 | 3 | 3 | 2 | 1 | 5656.7307 | 0.0002 |

| | | | | | | | |
|---|---|---|---|---|---|---|---|
| 4 | 1 | 3 | 3 | 2 | 2 | 5744.6707 | 0.0092 |
| 3 | 1 | 2 | 2 | 1 | 1 | 5776.2648 | 0.0024 |
| 5 | 1 | 5 | 4 | 2 | 3 | 5854.3973 | 0.0051 |
| 10 | 4 | 6 | 10 | 3 | 8 | 5932.0545 | -0.0056 |
| 3 | 1 | 3 | 2 | 0 | 2 | 5956.7608 | 0.0019 |
| 2 | 2 | 1 | 1 | 1 | 0 | 5984.3854 | 0.0032 |
| 2 | 2 | 0 | 1 | 1 | 0 | 6002.3649 | 0.0015 |
| 4 | 0 | 4 | 3 | 1 | 2 | 6016.7800 | 0.0063 |
| 2 | 2 | 1 | 1 | 1 | 1 | 6121.2648 | 0.0025 |
| 2 | 2 | 0 | 1 | 1 | 1 | 6139.2462 | 0.0027 |
| 3 | 1 | 2 | 2 | 0 | 2 | 6776.4999 | -0.0002 |
| 4 | 0 | 4 | 3 | 1 | 3 | 6836.5150 | 0.0001 |
| 2 | 2 | 0 | 1 | 0 | 1 | 6847.7420 | 0.0054 |
| 10 | 5 | 6 | 10 | 4 | 7 | 6848.4783 | 0.0049 |
| 6 | 5 | 2 | 6 | 4 | 3 | 6939.7597 | -0.0033 |
| 6 | 2 | 5 | 5 | 3 | 2 | 7016.4489 | -0.0086 |
| 4 | 1 | 4 | 3 | 1 | 3 | 7138.3481 | 0.0041 |
| 4 | 0 | 4 | 3 | 0 | 3 | 7280.2010 | 0.0027 |
| 4 | 3 | 1 | 4 | 0 | 4 | 7296.8877 | -0.0069 |
| 5 | 0 | 5 | 4 | 1 | 3 | 7352.0609 | 0.0087 |
| 4 | 2 | 3 | 3 | 2 | 2 | 7429.9763 | 0.0036 |
| 4 | 3 | 2 | 3 | 3 | 1 | 7476.0059 | 0.005 |
| 4 | 3 | 1 | 3 | 3 | 0 | 7484.8514 | 0.0056 |
| 4 | 1 | 4 | 3 | 0 | 3 | 7582.0292 | 0.002 |
| 4 | 2 | 2 | 3 | 2 | 1 | 7593.6202 | -0.0001 |
| 4 | 1 | 3 | 3 | 1 | 2 | 7676.9311 | 0.007 |
| 3 | 2 | 2 | 2 | 1 | 1 | 7708.5150 | -0.0099 |
| 3 | 2 | 1 | 2 | 1 | 1 | 7796.4595 | 0.0036 |
| 5 | 1 | 4 | 4 | 2 | 3 | 7866.7299 | -0.0012 |
| 3 | 2 | 2 | 2 | 1 | 2 | 8119.1469 | -0.0087 |
| 3 | 2 | 1 | 2 | 1 | 2 | 8207.0770 | -0.0096 |
| 5 | 0 | 5 | 4 | 1 | 4 | 8710.3755 | 0.0019 |
| 5 | 1 | 5 | 4 | 1 | 4 | 8898.0268 | 0.0019 |
| 4 | 1 | 3 | 3 | 0 | 3 | 8940.3428 | -0.0058 |
| 5 | 0 | 5 | 4 | 0 | 4 | 9012.1923 | -0.0103 |
| 5 | 1 | 5 | 4 | 0 | 4 | 9199.8449 | -0.0089 |
| 5 | 2 | 4 | 4 | 2 | 3 | 9265.0093 | 0.0065 |
| 5 | 3 | 3 | 4 | 3 | 2 | 9352.6909 | -0.002 |
| 4 | 2 | 3 | 3 | 1 | 2 | 9362.2373 | 0.002 |
| 3 | 3 | 1 | 2 | 2 | 0 | 9460.1643 | 0.0039 |
| 3 | 3 | 0 | 2 | 2 | 0 | 9461.6547 | 0.0026 |
| 3 | 3 | 1 | 2 | 2 | 1 | 9478.1431 | 0.0013 |
| 3 | 3 | 0 | 2 | 2 | 1 | 9479.6348 | 0.0014 |
| 4 | 2 | 3 | 3 | 1 | 3 | 10181.9734 | -0.003 |
| 4 | 2 | 2 | 3 | 1 | 3 | 10433.5604 | 0.0053 |
| 6 | 0 | 6 | 5 | 1 | 5 | 10538.4173 | 0.0083 |
| 5 | 2 | 4 | 4 | 1 | 3 | 10950.3033 | -0.0107 |
| 5 | 1 | 4 | 4 | 0 | 4 | 11212.2045 | 0.0116 |
| 4 | 3 | 1 | 3 | 2 | 1 | 11293.4656 | -0.0091 |
| 4 | 3 | 2 | 3 | 2 | 2 | 11371.0574 | -0.0117 |
| 4 | 3 | 1 | 3 | 2 | 2 | 11381.3967 | -0.009 |
| 5 | 2 | 3 | 4 | 1 | 3 | 11493.6651 | -0.0098 |
| rms | | | | | | | 5.11 kHz |

Table S3: Measured rotational transitions (Obs) for the $(Gly)_2$-($H_2^{18}O$) complex, and the residuals (Obs-Calc) in MHz.

| $J'$ | $K'_a$ | $K'_c$ | $J''$ | $K''_a$ | $K''_c$ | Obs | (Obs-Calc) |
|---|---|---|---|---|---|---|---|
| 2 | 2 | 1 | 2 | 1 | 2 | 2396.8331 | -0.0033 |
| 3 | 2 | 2 | 3 | 1 | 3 | 2638.7725 | -0.0110 |
| 1 | 1 | 0 | 0 | 0 | 0 | 2643.2437 | -0.0026 |
| 3 | 3 | 1 | 3 | 2 | 1 | 3513.0367 | -0.0070 |
| 2 | 1 | 2 | 1 | 1 | 1 | 3536.5242 | 0.0001 |
| 3 | 3 | 1 | 3 | 2 | 2 | 3628.3537 | 0.0059 |
| 3 | 3 | 0 | 3 | 2 | 2 | 3630.7140 | 0.0060 |
| 2 | 0 | 2 | 1 | 0 | 1 | 3664.7718 | 0.0000 |
| 4 | 3 | 2 | 4 | 2 | 3 | 3688.3396 | 0.0045 |
| 5 | 3 | 3 | 5 | 2 | 4 | 3801.1834 | 0.0039 |
| 3 | 1 | 2 | 2 | 2 | 1 | 3803.9194 | -0.0107 |
| 2 | 1 | 1 | 1 | 1 | 0 | 3840.6065 | -0.0021 |
| 2 | 1 | 2 | 1 | 0 | 1 | 4183.4331 | -0.0018 |
| 2 | 1 | 1 | 1 | 0 | 1 | 4639.5712 | 0.0043 |
| 3 | 0 | 3 | 2 | 1 | 2 | 4922.6122 | 0.0000 |
| 3 | 1 | 3 | 2 | 1 | 2 | 5290.8946 | 0.0012 |
| 3 | 0 | 3 | 2 | 0 | 2 | 5441.2766 | 0.0012 |
| 10 | 3 | 8 | 10 | 2 | 9 | 5518.8149 | -0.0010 |
| 3 | 2 | 2 | 2 | 2 | 1 | 5532.8436 | 0.0031 |
| 3 | 2 | 1 | 2 | 2 | 0 | 5624.3706 | 0.0128 |
| 2 | 2 | 1 | 1 | 1 | 0 | 5781.3114 | -0.0017 |
| 4 | 1 | 3 | 3 | 2 | 1 | 5781.5925 | -0.0006 |
| 2 | 2 | 0 | 1 | 1 | 0 | 5805.1003 | 0.0005 |
| 3 | 1 | 3 | 2 | 0 | 2 | 5809.5638 | 0.0073 |
| 2 | 2 | 1 | 1 | 1 | 1 | 5933.3517 | -0.0088 |
| 2 | 2 | 0 | 1 | 1 | 1 | 5957.1443 | -0.0027 |
| 2 | 2 | 0 | 1 | 0 | 1 | 6604.0628 | 0.0048 |
| 3 | 1 | 2 | 2 | 0 | 2 | 6719.4294 | -0.0002 |
| 4 | 0 | 4 | 3 | 1 | 3 | 6799.2672 | -0.0004 |
| 4 | 1 | 4 | 3 | 1 | 3 | 7031.6421 | -0.0017 |
| 4 | 0 | 4 | 3 | 0 | 3 | 7167.5435 | -0.0054 |
| 4 | 1 | 4 | 3 | 0 | 3 | 7399.9193 | -0.0057 |
| 4 | 3 | 1 | 3 | 3 | 0 | 7432.3459 | 0.0042 |
| 3 | 2 | 2 | 2 | 1 | 1 | 7473.5451 | 0.0001 |
| 4 | 2 | 2 | 3 | 2 | 1 | 7567.6601 | 0.0000 |
| 3 | 2 | 1 | 2 | 1 | 1 | 7588.8486 | -0.0003 |
| 4 | 1 | 3 | 3 | 1 | 2 | 7625.8143 | 0.0068 |
| 5 | 1 | 4 | 4 | 2 | 2 | 7685.4971 | 0.0015 |
| 3 | 2 | 2 | 2 | 1 | 2 | 7929.6742 | -0.0026 |
| 3 | 2 | 1 | 2 | 1 | 2 | 8044.9843 | 0.0035 |
| 5 | 0 | 5 | 4 | 1 | 4 | 8625.7546 | 0.0015 |
| 5 | 1 | 5 | 4 | 0 | 4 | 8990.9009 | -0.0056 |
| 4 | 2 | 3 | 3 | 1 | 2 | 9087.3451 | 0.0048 |
| 3 | 3 | 1 | 2 | 2 | 0 | 9137.4091 | 0.0074 |
| 3 | 3 | 0 | 2 | 2 | 0 | 9139.7643 | 0.0025 |
| 3 | 3 | 1 | 2 | 2 | 1 | 9161.1784 | -0.0098 |
| 3 | 3 | 0 | 2 | 2 | 1 | 9163.5502 | 0.0017 |
| 5 | 2 | 4 | 4 | 2 | 3 | 9168.4415 | 0.0001 |
| 4 | 2 | 2 | 3 | 1 | 2 | 9411.8782 | 0.0039 |
| 4 | 2 | 3 | 3 | 1 | 3 | 9997.2108 | -0.0027 |
| 10 | 5 | 6 | 9 | 6 | 4 | 10823.3164 | 0.0003 |
| 4 | 3 | 2 | 3 | 2 | 1 | 10931.4517 | -0.0094 |
| rms | | | | | | | 4.94 kHz |

Table S4: Experimentally determined rotational parameters for the $(Gly)_2$-$(H_2O)_2$ and singly $H_2{}^{18}O$ substituted complexes. A, B and C are the rotational constants. $\Delta_J$, $\Delta_{JK}$, $\Delta_K$, $\delta_J$, $\delta_K$ are the centrifugal distortion constants. $\sigma$ is the deviation of the fit. $N$ is the number of transitions in the fit.

| | $(Gly)_2$-$(H_2O)_2$ | $(Gly)_2$-$(H_2{}^{18}O1)$ $(H_2{}^{16}O2)$ | $(Gly)_2$-$(H_2{}^{16}O1)$ $(H_2{}^{18}O2)$ |
|---|---|---|---|
| A (MHz) | 1160.01937(62) | 1134.7853(10) | 1151.52770(81) |
| B (MHz) | 860.74300(26) | 853.54700(45) | 835.10608(32) |
| C (MHz) | 659.75702(27) | 652.54138(47) | 643.11861(34) |
| $\Delta_J$ (kHz) | 0.1264(30) | 0.1345(66) | 0.1014(49) |
| $\Delta_{JK}$ (kHz) | 1.172(20) | 1.004(30) | 1.151(37) |
| $\Delta_K$ (kHz) | -0.491(36) | - | - |
| $\delta_J$ (kHz) | - | - | - |
| $\delta_K$ (kHz) | - | - | - |
| $\sigma$ (kHz) | 6.8 | 7.0 | 5.0 |
| $N$ | 82 | 36 | 38 |

Table S5: Measured rotational transitions (Obs) for the $(Gly)_2$-$(H_2O)_2$ complex, and the residuals (Obs-Calc) in MHz.

| J′ | $K'_a$ | $K'_c$ | J″ | $K''_a$ | $K''_c$ | Obs | (Obs-Calc) |
|---|---|---|---|---|---|---|---|
| 4 | 3 | 2 | 4 | 2 | 3 | 2203.2879 | 0.0023 |
| 5 | 4 | 1 | 5 | 3 | 2 | 2398.9509 | 0.0073 |
| 5 | 3 | 3 | 5 | 2 | 4 | 2483.2660 | -0.0030 |
| 5 | 4 | 2 | 5 | 3 | 3 | 2748.6626 | -0.0106 |
| 2 | 1 | 2 | 1 | 1 | 1 | 2840.0041 | -0.0012 |
| 6 | 4 | 3 | 6 | 3 | 4 | 2866.7453 | 0.0079 |
| 6 | 3 | 4 | 6 | 2 | 5 | 2881.1610 | -0.0020 |
| 2 | 0 | 2 | 1 | 0 | 1 | 2968.4977 | 0.0006 |
| 2 | 1 | 2 | 1 | 0 | 1 | 3139.2743 | -0.0055 |
| 2 | 1 | 1 | 1 | 1 | 0 | 3241.9890 | 0.0116 |
| 6 | 2 | 5 | 6 | 1 | 6 | 3418.4418 | 0.0091 |
| 2 | 2 | 1 | 1 | 1 | 0 | 4139.7876 | -0.0051 |
| 3 | 0 | 3 | 2 | 1 | 2 | 4146.8547 | 0.0022 |
| 3 | 1 | 3 | 2 | 1 | 2 | 4220.5561 | -0.0006 |
| 3 | 0 | 3 | 2 | 0 | 2 | 4317.6339 | -0.0013 |
| 2 | 2 | 1 | 1 | 1 | 1 | 4340.7791 | 0.0004 |
| 3 | 1 | 3 | 2 | 0 | 2 | 4391.3311 | -0.0085 |
| 2 | 2 | 0 | 1 | 1 | 1 | 4413.2815 | 0.0038 |
| 3 | 2 | 2 | 2 | 2 | 1 | 4561.4725 | 0.0141 |
| 3 | 2 | 1 | 2 | 2 | 0 | 4805.3193 | 0.0098 |
| 3 | 1 | 2 | 2 | 1 | 1 | 4805.8466 | 0.0032 |
| 3 | 2 | 2 | 2 | 1 | 1 | 5459.2668 | -0.0068 |
| 4 | 0 | 4 | 3 | 1 | 3 | 5546.8831 | -0.0006 |
| 4 | 1 | 4 | 3 | 1 | 3 | 5573.1412 | -0.0035 |
| 4 | 0 | 4 | 3 | 0 | 3 | 5620.5841 | -0.0039 |

| | | | | | | | |
|---|---|---|---|---|---|---|---|
| 4 | 1 | 3 | 3 | 2 | 2 | 5628.1993 | 0.0043 |
| 4 | 1 | 4 | 3 | 0 | 3 | 5646.8444 | -0.0047 |
| 4 | 2 | 3 | 3 | 2 | 2 | 6023.6947 | 0.0033 |
| 3 | 2 | 2 | 2 | 1 | 2 | 6062.2437 | 0.0121 |
| 4 | 3 | 2 | 3 | 3 | 1 | 6188.7410 | 0.0160 |
| 4 | 1 | 3 | 3 | 1 | 2 | 6281.6205 | -0.0046 |
| 4 | 3 | 1 | 3 | 3 | 0 | 6284.2037 | 0.0152 |
| 4 | 2 | 2 | 3 | 2 | 1 | 6481.5362 | 0.0016 |
| 3 | 3 | 1 | 2 | 2 | 0 | 6527.2108 | -0.0006 |
| 3 | 3 | 1 | 2 | 2 | 1 | 6599.7162 | 0.0058 |
| 3 | 3 | 0 | 2 | 2 | 1 | 6617.3841 | 0.0024 |
| 5 | 2 | 3 | 4 | 3 | 2 | 6664.8267 | -0.0103 |
| 4 | 2 | 3 | 3 | 1 | 2 | 6677.1151 | -0.0066 |
| 5 | 0 | 5 | 4 | 1 | 4 | 6898.8685 | -0.0011 |
| 5 | 1 | 5 | 4 | 1 | 4 | 6907.2335 | -0.0023 |
| 5 | 0 | 5 | 4 | 0 | 4 | 6925.1321 | 0.0013 |
| 5 | 1 | 5 | 4 | 0 | 4 | 6933.4995 | 0.0025 |
| 10 | 3 | 8 | 9 | 5 | 5 | 7179.5426 | -0.0011 |
| 5 | 1 | 4 | 4 | 2 | 3 | 7249.3984 | -0.0035 |
| 5 | 2 | 4 | 4 | 2 | 3 | 7443.1205 | -0.0014 |
| 4 | 2 | 2 | 3 | 1 | 2 | 7451.3146 | -0.0004 |
| 5 | 1 | 4 | 4 | 1 | 3 | 7644.8886 | -0.0098 |
| 5 | 3 | 3 | 4 | 3 | 2 | 7723.1031 | -0.0021 |
| 5 | 4 | 1 | 4 | 4 | 0 | 7788.0302 | 0.0139 |
| 5 | 2 | 4 | 4 | 1 | 3 | 7838.6175 | -0.0008 |
| 4 | 2 | 3 | 3 | 1 | 3 | 7865.3786 | 0.0124 |
| 7 | 4 | 3 | 6 | 5 | 2 | 7880.8237 | -0.0006 |
| 4 | 3 | 2 | 3 | 2 | 1 | 7910.6166 | -0.0102 |
| 5 | 3 | 2 | 4 | 3 | 1 | 7989.7334 | -0.0018 |
| 4 | 3 | 1 | 3 | 2 | 1 | 8023.7579 | -0.0038 |
| 5 | 2 | 3 | 4 | 2 | 2 | 8093.9268 | -0.0025 |
| 4 | 3 | 2 | 3 | 2 | 2 | 8226.9778 | 0.0008 |
| 6 | 0 | 6 | 5 | 1 | 5 | 8229.6027 | 0.0033 |
| 6 | 1 | 6 | 5 | 1 | 5 | 8232.0941 | 0.0054 |
| 6 | 0 | 6 | 5 | 0 | 5 | 8237.9686 | 0.0029 |
| 6 | 1 | 6 | 5 | 0 | 5 | 8240.4600 | 0.0050 |
| 6 | 1 | 5 | 5 | 2 | 4 | 8743.2373 | -0.0060 |
| 6 | 2 | 5 | 5 | 2 | 4 | 8822.4105 | -0.0034 |
| 4 | 4 | 1 | 3 | 3 | 0 | 8881.3096 | -0.0049 |
| 4 | 4 | 0 | 3 | 3 | 0 | 8884.8526 | 0.0016 |
| 4 | 4 | 1 | 3 | 3 | 1 | 8898.9850 | -0.0009 |
| 4 | 4 | 0 | 3 | 3 | 1 | 8902.5222 | -0.0001 |
| 6 | 2 | 5 | 5 | 1 | 4 | 9016.1251 | -0.0087 |
| 5 | 3 | 3 | 4 | 2 | 2 | 9152.1972 | -0.0003 |
| 6 | 3 | 4 | 5 | 3 | 3 | 9220.3085 | 0.0005 |
| 5 | 2 | 3 | 4 | 1 | 3 | 9263.6106 | -0.0085 |
| 6 | 4 | 3 | 5 | 4 | 2 | 9338.3621 | -0.0099 |
| 6 | 4 | 2 | 5 | 4 | 1 | 9442.9699 | -0.0135 |
| 7 | 0 | 7 | 6 | 1 | 6 | 9552.5176 | 0.0025 |
| 7 | 1 | 7 | 6 | 1 | 6 | 9553.2350 | 0.0124 |

| | | | | | | | |
|---|---|---|---|---|---|---|---|
| 7 | 0 | 7 | 6 | 0 | 6 | 9555.0123 | 0.0080 |
| 7 | 1 | 7 | 6 | 0 | 6 | 9555.7230 | 0.0112 |
| 6 | 2 | 4 | 5 | 2 | 3 | 9602.6084 | -0.0118 |
| 7 | 1 | 6 | 6 | 2 | 5 | 10143.2963 | 0.0101 |
| 7 | 1 | 6 | 6 | 1 | 5 | 10222.4542 | -0.0026 |
| 7 | 2 | 6 | 6 | 1 | 5 | 10251.0419 | -0.0088 |
| 5 | 4 | 2 | 4 | 3 | 1 | 10358.6421 | -0.0015 |
| rms | | | | | | | 6.80 kHz |

Table S6: Measured rotational transitions (Obs) for the $(Gly)_2$-$(H_2{}^{18}O1)(H_2{}^{16}O2)$ complex, and the residuals (Obs-Calc) in MHz.

| $J'$ | $K'_a$ | $K'_c$ | $J''$ | $K''_a$ | $K''_c$ | Obs | (Obs-Calc) |
|---|---|---|---|---|---|---|---|
| 3 | 0 | 3 | 2 | 1 | 2 | 4111.1112 | 0.0039 |
| 2 | 2 | 0 | 1 | 1 | 0 | 4132.5108 | 0.0053 |
| 3 | 1 | 3 | 2 | 1 | 2 | 4175.7584 | -0.0043 |
| 3 | 0 | 3 | 2 | 0 | 2 | 4266.9640 | -0.0043 |
| 3 | 1 | 3 | 2 | 0 | 2 | 4331.6136 | -0.0103 |
| 3 | 1 | 2 | 2 | 1 | 1 | 4759.4242 | 0.0069 |
| 4 | 0 | 4 | 3 | 1 | 3 | 5490.0160 | 0.0005 |
| 4 | 1 | 4 | 3 | 1 | 3 | 5512.1174 | -0.0016 |
| 4 | 0 | 4 | 3 | 0 | 3 | 5554.6635 | -0.0075 |
| 4 | 1 | 4 | 3 | 0 | 3 | 5576.7629 | -0.0116 |
| 4 | 1 | 3 | 3 | 2 | 2 | 5610.1966 | 0.0142 |
| 4 | 2 | 3 | 3 | 2 | 2 | 5963.3561 | 0.0015 |
| 3 | 2 | 2 | 2 | 1 | 2 | 5964.9423 | 0.0019 |
| 4 | 1 | 3 | 3 | 1 | 2 | 6212.6998 | 0.0111 |
| 4 | 3 | 1 | 3 | 3 | 0 | 6237.8876 | 0.0072 |
| 3 | 3 | 1 | 2 | 2 | 0 | 6392.2131 | 0.0019 |
| 4 | 2 | 2 | 3 | 2 | 1 | 6428.9368 | 0.0020 |
| 3 | 3 | 0 | 2 | 2 | 1 | 6487.2094 | 0.0015 |
| 5 | 0 | 5 | 4 | 1 | 4 | 6823.6952 | 0.0036 |
| 5 | 1 | 5 | 4 | 1 | 4 | 6830.4505 | -0.0015 |
| 5 | 0 | 5 | 4 | 0 | 4 | 6845.7975 | 0.0022 |
| 5 | 1 | 5 | 4 | 0 | 4 | 6852.5536 | -0.0021 |
| 5 | 1 | 4 | 4 | 2 | 3 | 7198.5520 | 0.0039 |
| 5 | 2 | 4 | 4 | 2 | 3 | 7364.3363 | -0.0143 |
| 5 | 1 | 4 | 4 | 1 | 3 | 7551.7085 | -0.0116 |
| 5 | 3 | 3 | 4 | 3 | 2 | 7652.2397 | 0.0003 |
| 4 | 3 | 2 | 3 | 2 | 1 | 7757.1250 | -0.0047 |
| 5 | 3 | 2 | 4 | 3 | 1 | 7935.2211 | -0.0088 |
| 5 | 2 | 3 | 4 | 2 | 2 | 8018.6511 | -0.0132 |
| 6 | 0 | 6 | 5 | 1 | 5 | 8138.2400 | 0.0060 |
| 6 | 1 | 6 | 5 | 1 | 5 | 8140.1742 | 0.0082 |
| 6 | 0 | 6 | 5 | 0 | 5 | 8145.0024 | 0.0079 |
| 6 | 1 | 6 | 5 | 0 | 5 | 8146.9350 | 0.0085 |
| 4 | 3 | 1 | 3 | 2 | 2 | 8206.8674 | 0.0056 |
| 7 | 1 | 6 | 6 | 2 | 5 | 10034.3127 | -0.0043 |
| 8 | 4 | 4 | 7 | 5 | 3 | 10029.1254 | -0.0019 |
| MW rms | | | | | | | 7.04 kHz |

Table S7: Measured rotational transitions (Obs) for the $(Gly)_2$-($H_2{}^{16}O1$)($H_2{}^{18}O2$) complex, and the residuals (Obs-Calc) in MHz.

| J′ | $K'_a$ | $K'_c$ | J″ | $K''_a$ | $K''_c$ | Obs | (Obs-Calc) |
|---|---|---|---|---|---|---|---|
| 2 | 0 | 2 | 1 | 0 | 1 | 2891.9379 | 0.0005 |
| 3 | 0 | 3 | 2 | 1 | 2 | 4024.5449 | -0.0044 |
| 2 | 2 | 1 | 1 | 1 | 0 | 4097.6689 | -0.0038 |
| 3 | 1 | 3 | 2 | 1 | 2 | 4111.3321 | 0.0029 |
| 3 | 0 | 3 | 2 | 0 | 2 | 4213.4849 | -0.0004 |
| 3 | 1 | 3 | 2 | 0 | 2 | 4300.2634 | -0.0018 |
| 2 | 2 | 0 | 1 | 1 | 1 | 4354.1734 | 0.0043 |
| 3 | 1 | 2 | 2 | 1 | 1 | 4672.7970 | 0.0034 |
| 3 | 2 | 2 | 2 | 1 | 1 | 5383.8792 | -0.0002 |
| 4 | 0 | 4 | 3 | 1 | 3 | 5399.0060 | 0.0001 |
| 4 | 1 | 4 | 3 | 1 | 3 | 5432.0381 | 0.0042 |
| 4 | 0 | 4 | 3 | 0 | 3 | 5485.7833 | -0.0023 |
| 4 | 1 | 4 | 3 | 0 | 3 | 5518.8147 | 0.0010 |
| 4 | 2 | 3 | 3 | 2 | 2 | 5861.2468 | 0.0101 |
| 7 | 0 | 7 | 6 | 1 | 5 | 6053.5791 | 0.0011 |
| 4 | 1 | 3 | 3 | 1 | 2 | 6120.3894 | -0.0078 |
| 3 | 2 | 1 | 2 | 1 | 2 | 6245.5208 | -0.0076 |
| 4 | 2 | 2 | 3 | 2 | 1 | 6285.6452 | -0.0013 |
| 3 | 3 | 1 | 2 | 2 | 0 | 6467.4796 | -0.0003 |
| 3 | 3 | 0 | 2 | 2 | 0 | 6481.9831 | 0.0053 |
| 3 | 3 | 1 | 2 | 2 | 1 | 6531.9938 | 0.0049 |
| 3 | 3 | 0 | 2 | 2 | 1 | 6546.4992 | 0.0125 |
| 5 | 0 | 5 | 4 | 1 | 4 | 6723.1983 | -0.0024 |
| 5 | 1 | 5 | 4 | 1 | 4 | 6734.4317 | 0.0006 |
| 5 | 0 | 5 | 4 | 0 | 4 | 6756.2286 | -0.0002 |
| 5 | 1 | 5 | 4 | 0 | 4 | 6767.4607 | 0.0015 |
| 8 | 6 | 3 | 7 | 7 | 0 | 6899.1065 | 0.0033 |
| 5 | 2 | 4 | 4 | 2 | 3 | 7249.0865 | -0.0012 |
| 4 | 2 | 2 | 3 | 1 | 2 | 7282.4151 | -0.0041 |
| 5 | 1 | 4 | 4 | 1 | 3 | 7464.4843 | -0.0140 |
| 5 | 3 | 3 | 4 | 3 | 2 | 7504.0965 | 0.0071 |
| 6 | 0 | 6 | 5 | 1 | 5 | 8023.4751 | 0.0015 |
| 6 | 1 | 6 | 5 | 1 | 5 | 8027.0417 | 0.0043 |
| 6 | 0 | 6 | 5 | 0 | 5 | 8034.7022 | -0.0017 |
| 6 | 1 | 6 | 5 | 0 | 5 | 8038.2666 | -0.0010 |
| 6 | 2 | 5 | 5 | 2 | 4 | 8599.1975 | -0.0100 |
| 6 | 3 | 4 | 5 | 3 | 3 | 8967.0326 | 0.0011 |
| 7 | 0 | 7 | 6 | 1 | 6 | 9314.3189 | -0.0011 |
| rms | | | | | | | 5.01 kHz |

Table S8: Experimentally determined rotational parameters for the $(Gly)_2$-$(H_2O)_3$ and singly $H_2{}^{18}O$ substituted complexes. A, B and C are the rotational constants. $\Delta_J$, $\Delta_{JK}$, $\Delta_K$, $\delta_J$, $\delta_K$ are the centrifugal distortion constants. $\sigma$ is the deviation of the fit. $N$ is the number of transitions in the fit. Parameters in square brackets were fixed at the parent species values.

| | $(Gly)_2$-$(H_2O)_3$ | $(Gly)_2$-$(H_2{}^{18}O1)$ $(H_2{}^{16}O2)(H_2{}^{16}O3)$ | $(Gly)_2$-$(H_2{}^{16}O1)$ $(H_2{}^{18}O2)(H_2{}^{16}O3)$ | $(Gly)_2$-$(H_2{}^{16}O1)$ $(H_2{}^{16}O2)(H_2{}^{18}O3)$ |
|---|---|---|---|---|
| A (MHz) | 789.9518(16) | 782.4049(37) | 788.7531(35) | 782.3840(47) |
| B (MHz) | 673.87562(84) | 659.49682(75) | 651.8477(14) | 666.63037(84) |
| C (MHz) | 507.12181(52) | 501.73438(51) | 494.85508(46) | 506.01417(65) |
| $\Delta_J$ (kHz) | 0.2670(77) | [0.2670(77)] | [0.2670(77)] | [0.2670(77)] |
| $\Delta_{JK}$ (kHz) | -0.597(34) | [-0.597(34)] | [-0.597(34)] | [-0.597(34)] |
| $\Delta_K$ (kHz) | - | - | - | - |
| $\delta_J$ (kHz) | 0.0997(39) | [0.0997(39)] | [0.0997(39)] | [0.0997(39)] |
| $\delta_K$ (kHz) | -0.113(29) | [-0.113(29)] | [-0.113(29)] | [-0.113(29)] |
| $\sigma$ (kHz) | 8.25 | 12.75 | 13.65 | 13.03 |
| $N$ | 42 | 26 | 21 | 21 |

Table S9: Measured rotational transitions (Obs) for the $(Gly)_2$-$(H_2O)_3$ complex, and the residuals (Obs-Calc) in MHz.

| $J'$ | $K'_a$ | $K'_c$ | $J''$ | $K''_a$ | $K''_c$ | Obs | (Obs-Calc) |
|---|---|---|---|---|---|---|---|
| 3 | 1 | 3 | 2 | 1 | 2 | 3244.2244 | -0.0028 |
| 3 | 0 | 3 | 2 | 0 | 2 | 3276.3732 | 0.0059 |
| 3 | 2 | 2 | 2 | 2 | 1 | 3542.9778 | 0.0045 |
| 3 | 1 | 2 | 2 | 1 | 1 | 3704.9852 | 0.0034 |
| 4 | 1 | 4 | 3 | 1 | 3 | 4270.5028 | -0.007 |
| 4 | 0 | 4 | 3 | 0 | 3 | 4279.0485 | -0.0011 |
| 4 | 2 | 3 | 3 | 2 | 2 | 4645.2126 | -0.0045 |
| 4 | 1 | 3 | 3 | 1 | 2 | 4762.9065 | 0.0132 |
| 4 | 3 | 2 | 3 | 3 | 1 | 4843.9048 | -0.0025 |
| 4 | 2 | 2 | 3 | 2 | 1 | 5076.3494 | -0.0056 |
| 4 | 3 | 1 | 3 | 3 | 0 | 5018.4670 | -0.0123 |
| 5 | 1 | 5 | 4 | 1 | 4 | 5287.7818 | -0.0022 |
| 5 | 0 | 5 | 4 | 0 | 4 | 5289.5865 | -0.0023 |
| 5 | 2 | 4 | 4 | 2 | 3 | 5702.8965 | 0.0034 |
| 5 | 1 | 4 | 4 | 1 | 3 | 5752.6841 | 0.0074 |
| 5 | 3 | 3 | 4 | 3 | 2 | 6004.3126 | -0.0087 |
| 5 | 2 | 3 | 4 | 2 | 2 | 6229.5855 | 0.0036 |
| 6 | 1 | 6 | 5 | 1 | 5 | 6302.5859 | -0.0028 |
| 6 | 0 | 6 | 5 | 0 | 5 | 6302.9253 | -0.0052 |
| 5 | 3 | 2 | 4 | 3 | 1 | 6368.8076 | -0.0061 |
| 6 | 2 | 5 | 5 | 2 | 4 | 6732.8942 | 0.0008 |
| 6 | 1 | 5 | 5 | 1 | 4 | 6747.3358 | 0.0061 |
| 6 | 3 | 4 | 5 | 3 | 3 | 7109.9637 | -0.0132 |
| 6 | 2 | 4 | 5 | 2 | 3 | 7254.5003 | 0.0025 |

| | | | | | | | |
|---|---|---|---|---|---|---|---|
| 6 | 4 | 3 | 5 | 4 | 2 | 7318.6894 | 0.0008 |
| 6 | 5 | 1 | 5 | 5 | 0 | 7381.2620 | 0.0081 |
| 6 | 4 | 2 | 5 | 4 | 1 | 7578.6695 | 0.0019 |
| 6 | 3 | 3 | 5 | 3 | 2 | 7629.0913 | 0.0017 |
| 7 | 2 | 6 | 6 | 2 | 5 | 7751.2462 | 0.0234 |
| 7 | 1 | 6 | 6 | 1 | 5 | 7754.6655 | 0.0056 |
| 5 | 3 | 2 | 4 | 1 | 3 | 7896.0068 | 0.0065 |
| 7 | 3 | 5 | 6 | 3 | 4 | 8168.4508 | -0.0008 |
| 7 | 2 | 5 | 6 | 2 | 4 | 8228.0343 | -0.0136 |
| 8 | 0 | 8 | 7 | 0 | 7 | 8331.0442 | -0.0049 |
| 8 | 1 | 8 | 7 | 1 | 7 | 8331.0342 | 0.0051 |
| 7 | 5 | 3 | 6 | 5 | 2 | 8592.9095 | 0.0029 |
| 7 | 3 | 4 | 6 | 3 | 3 | 8749.3919 | -0.0219 |
| 8 | 2 | 7 | 7 | 2 | 6 | 8766.1030 | 0.0059 |
| 8 | 1 | 7 | 7 | 1 | 6 | 8766.8233 | 0.008 |
| 7 | 4 | 3 | 6 | 4 | 2 | 8939.6048 | 0.0049 |
| 9 | 0 | 9 | 8 | 0 | 8 | 9345.1913 | -0.0055 |
| 9 | 1 | 9 | 8 | 1 | 8 | 9345.1897 | -0.0039 |
| rms | | | | | | | 8.25 kHz |

Table S10: Measured rotational transitions (Obs) for the $(Gly)_2$-$(H_2{}^{18}O1)(H_2{}^{16}O2)(H_2{}^{16}O3)$ complex, and the residuals (Obs-Calc) in MHz.

| J′ | $K_a'$ | $K_c'$ | J″ | $K_a''$ | $K_c''$ | Obs | (Obs-Calc) |
|---|---|---|---|---|---|---|---|
| 3 | 1 | 3 | 2 | 1 | 2 | 3203.2474 | -0.001 |
| 3 | 0 | 3 | 2 | 0 | 2 | 3238.8021 | 0.0005 |
| 3 | 1 | 2 | 2 | 1 | 1 | 3643.7016 | 0.0234 |
| 4 | 1 | 4 | 3 | 1 | 3 | 4219.8194 | -0.0056 |
| 4 | 0 | 4 | 3 | 0 | 3 | 4230.2123 | -0.0023 |
| 4 | 2 | 3 | 3 | 2 | 2 | 4574.9978 | 0.0283 |
| 4 | 1 | 3 | 3 | 1 | 2 | 4699.9235 | 0.0092 |
| 5 | 1 | 5 | 4 | 1 | 4 | 5226.9093 | -0.0078 |
| 5 | 0 | 5 | 4 | 0 | 4 | 5229.3111 | -0.0061 |
| 5 | 2 | 4 | 4 | 2 | 3 | 5624.6215 | 0.0015 |
| 5 | 1 | 4 | 4 | 1 | 3 | 5683.2773 | 0.0110 |
| 5 | 3 | 3 | 4 | 3 | 2 | 5901.9160 | 0.0129 |
| 6 | 1 | 6 | 5 | 1 | 5 | 6231.1271 | -0.0074 |
| 5 | 3 | 2 | 4 | 3 | 1 | 6231.2980 | -0.0161 |
| 6 | 0 | 6 | 5 | 0 | 5 | 6231.6270 | 0.0000 |
| 6 | 2 | 5 | 5 | 2 | 4 | 6646.3811 | -0.001 |
| 6 | 1 | 5 | 5 | 1 | 4 | 6665.2027 | -0.0018 |
| 6 | 3 | 4 | 5 | 3 | 3 | 7000.1922 | -0.0278 |
| 6 | 2 | 4 | 5 | 2 | 3 | 7161.5800 | -0.0115 |
| 6 | 4 | 3 | 5 | 4 | 2 | 7182.5169 | -0.0116 |
| 6 | 5 | 2 | 5 | 5 | 1 | 7197.2761 | 0.0177 |
| 6 | 5 | 1 | 5 | 5 | 0 | 7224.0247 | 0.0000 |
| 7 | 0 | 7 | 6 | 0 | 6 | 7234.7579 | -0.0021 |
| 7 | 1 | 7 | 6 | 1 | 6 | 7234.6793 | 0.0134 |
| 7 | 2 | 6 | 6 | 2 | 5 | 7655.2081 | -0.0018 |
| 7 | 1 | 6 | 6 | 1 | 5 | 7660.1036 | 0.0041 |
| rms | | | | | | | 12.75 kHz |

Table S11: Measured rotational transitions (Obs) for the $(Gly)_2$-$(H_2{}^{16}O1)(H_2{}^{18}O2)(H_2{}^{16}O3)$ complex, and the residuals (Obs-Calc) in MHz.

| J′ | $K_a'$ | $K_c'$ | J″ | $K_a''$ | $K_c''$ | Obs | (Obs-Calc) |
|---|---|---|---|---|---|---|---|
| 3 | 1 | 3 | 2 | 1 | 2 | 3163.2569 | -0.0074 |
| 3 | 0 | 3 | 2 | 0 | 2 | 3204.3465 | -0.0138 |
| 3 | 1 | 2 | 2 | 1 | 1 | 3605.6857 | 0.004 |
| 4 | 1 | 4 | 3 | 1 | 3 | 4167.9131 | 0.0123 |
| 8 | 2 | 7 | 7 | 4 | 4 | 4328.4457 | -0.0147 |
| 4 | 2 | 3 | 3 | 2 | 2 | 4521.6750 | -0.0043 |
| 4 | 1 | 3 | 3 | 1 | 2 | 4659.8955 | 0.0062 |
| 4 | 2 | 2 | 3 | 2 | 1 | 4918.8297 | -0.0296 |
| 5 | 1 | 5 | 4 | 1 | 4 | 5162.1373 | 0.0113 |
| 5 | 0 | 5 | 4 | 0 | 4 | 5165.4643 | 0.0087 |

| | | | | | | | |
|---|---|---|---|---|---|---|---|
| 5 | 2 | 4 | 4 | 2 | 3 | 5562.6419 | -0.0101 |
| 5 | 1 | 4 | 4 | 1 | 3 | 5634.3947 | -0.035 |
| 5 | 3 | 3 | 4 | 3 | 2 | 5829.9870 | 0.0014 |
| 5 | 3 | 2 | 4 | 3 | 1 | 6140.3910 | 0.0146 |
| 6 | 0 | 6 | 5 | 0 | 5 | 6153.6547 | 0.0242 |
| 6 | 1 | 6 | 5 | 1 | 5 | 6152.9023 | 0.0164 |
| 6 | 2 | 5 | 5 | 2 | 4 | 6574.4239 | -0.0183 |
| 6 | 1 | 5 | 5 | 1 | 4 | 6599.9230 | -0.022 |
| 7 | 1 | 7 | 6 | 1 | 6 | 7142.7703 | 0.0347 |
| 7 | 0 | 7 | 6 | 0 | 6 | 7142.9190 | 0.0285 |
| 7 | 2 | 6 | 6 | 2 | 5 | 7571.3444 | -0.0242 |
| rms | | | | | | | 13.65 kHz |

Table S12: Measured rotational transitions (Obs) for the $(Gly)_2$-$(H_2{}^{16}O1)(H_2{}^{16}O2)(H_2{}^{18}O3)$ complex, and the residuals (Obs-Calc) in MHz.

| $J'$ | $K'_a$ | $K'_c$ | $J''$ | $K''_a$ | $K''_c$ | Obs | (Obs-Calc) |
|---|---|---|---|---|---|---|---|
| 3 | 1 | 3 | 2 | 1 | 2 | 3230.8542 | 0.0007 |
| 3 | 0 | 3 | 2 | 0 | 2 | 3263.3664 | -0.0004 |
| 3 | 1 | 2 | 2 | 1 | 1 | 3676.1341 | 0.015 |
| 4 | 1 | 4 | 3 | 1 | 3 | 4254.9923 | -0.0022 |
| 4 | 0 | 4 | 3 | 0 | 3 | 4263.8951 | 0.0017 |
| 4 | 1 | 3 | 3 | 1 | 2 | 4733.7457 | 0.0145 |
| 4 | 2 | 2 | 3 | 2 | 1 | 5030.4012 | 0.0287 |
| 5 | 1 | 5 | 4 | 1 | 4 | 5270.1644 | 0.0018 |
| 5 | 0 | 5 | 4 | 0 | 4 | 5272.0939 | -0.0006 |
| 5 | 2 | 4 | 4 | 2 | 3 | 5671.5949 | 0.0043 |
| 5 | 3 | 3 | 4 | 3 | 2 | 5959.5486 | 0.012 |
| 5 | 2 | 3 | 4 | 2 | 2 | 6181.5666 | -0.0276 |
| 6 | 1 | 6 | 5 | 1 | 5 | 6282.7947 | 0.0095 |
| 6 | 0 | 6 | 5 | 0 | 5 | 6283.1636 | 0.0053 |
| 5 | 3 | 2 | 4 | 3 | 1 | 6306.0501 | 0.0019 |
| 6 | 2 | 5 | 5 | 2 | 4 | 6699.7921 | -0.0108 |
| 6 | 1 | 5 | 5 | 1 | 4 | 6715.1565 | -0.0128 |
| 6 | 4 | 3 | 5 | 4 | 2 | 7258.6148 | 0.0091 |
| 6 | 4 | 2 | 5 | 4 | 1 | 7500.9338 | -0.0177 |
| 7 | 2 | 6 | 6 | 2 | 5 | 7716.2041 | -0.003 |
| 7 | 1 | 6 | 6 | 1 | 5 | 7719.9422 | -0.0072 |
| rms | | | | | | | 13.03 kHz |

## 1.2 Structural Analysis

Complete structure analysis, even of the relative orientations of the component molecules in the studied clusters, requires observation of a considerably greater number of isotopic species than is available from $^{18}O$ substitution. Relative orientations of two complexed molecules already require six structural parameters for description, specifically the length and two angles for the centre of mass separation vector, and three rotation angles of the second molecule relative to the first one. Adding each further molecule increases the parameter count correspondingly.

It is possible, however, to use a hybrid approach combining information from *ab initio* calculations and experiment. Observation of single $H_2{}^{18}O$ isotopic species of the cluster delivers rather precise values of coordinates of the substituted atom. These can then be used to assess the consistency of experiment with global minimum geometries from computations. Successful calibration of this type then allows exploration of least-squares fitting models for refining the calculated structure for closest agreement with experiment. The criteria in seeking such models are to identify parameters in the structural fit that are most efficient in reducing the deviation of fit, while avoiding overfitting the experimental data. This means that there should be a significantly smaller number of parameters of fit than of the available number of rotational constants. The reliability of the fitted geometries can, again, be assessed by comparing the resulting principal coordinates of the oxygen atoms in the water molecules, as carried out in Table S13.

Only the least-squares fit of an $r_0$ geometry is realistic at present and appropriate assumptions concerning the geometries of constituent molecules are necessary. The preference in such cases is to use $r_0$ geometries for the monomers. For glycolaldehyde we used the B3LYP/6311++G(d,p) calculated monomer geometry, which accidentally closely reproduces the experimental ground state rotational constants, $A_0$, $B_0$, $C_0$. The only change from the $C_s$ geometry of the isolated monomer to that in the $Gly_2$ has been to change the dihedral angle $D$(HOCC) to the calculated values for the hydrated clusters. Similarly, the $r_0$ geometry for water (F.C.De Lucia et al., Phys.Rev.A 8,2785(1973)) was used. The lightest cluster, $Gly_2$-$H_2O$, was an exception to this procedure since we found that the use of the calculated geometry gave the best deviation of fit at the most reasonable values of the principal coordinate of the water oxygen atom.

The plausible cluster geometries derived in this way are compared in Figures 2 and S1 and there is good consistency between the principal coordinates in Table S13. It is noted that the $r_0$ coordinates have a significantly greater uncertainty than the $r_s$ coordinates, but this is the price that has to be paid for deriving structural information on the whole cluster and not only on a single atom. All determined structures share the same bifurcated intermolecular bonding motif that appears in the glycolaldehyde dimer unit on addition of even only one $H_2O$ molecule.

Table S13: Comparison of the values of principal coordinates (Å) for the oxygen atoms in the complexed water molecules in Gly$_2$-(H$_2$O)$_n$ clusters.

| | $a$ | $b$ | $c$ |
|---|---|---|---|
| (Gly)$_2$-H$_2$O: | | | |
| | | O1 = atom 17 | |
| calc.[a] | 0.3041 | 2.2899 | 0.5437 |
| $r_s$[b,c] | 0.2364(63) | 2.3496(7) | 0.4036(44) |
| $r_0$ | 0.277(37) | 2.347(6) | 0.376(37) |
| (Gly)$_2$-(H$_2$O)$_2$: | | | |
| | | O1 = atom 20 | |
| calc. | 2.9560 | 1.1488 | -0.4945 |
| $r_s$ | 2.9304(10) | 1.1848(27) | -0.6187(50) |
| $r_0$[d] | 2.932(14) | 1.230(23) | -0.578(24) |
| | | O2 = atom 17 | |
| calc. | 0.8730 | 1.7893 | 1.2768 |
| $r_s$ | 0.9453(30) | 1.8222(16) | 1.2748(24) |
| $r_0$[d] | 0.965(40) | 1.838(21) | 1.266(34) |
| (Gly)$_2$-(H$_2$O)$_3$: | | | |
| | | O1 = atom 23 | |
| calc. | 2.4269 | 0.2601 | -1.6672 |
| $r_s$ | 2.2729(7) | 0.3799(40) | -1.7516(9) |
| $r_0$[e] | 2.287(12) | 0.413(71) | -1.756(17) |
| | | O2 = atom 20 | |
| calc. | 3.3456 | -0.6575 | 0.7663 |
| $r_s$ | 3.5103(4) | -0.3326(45) | 0.6667(23) |
| $r_0$[e] | 3.520(7) | -0.376(79) | 0.659(31) |
| | | O3 = atom 17 | |
| calc. | 0.9197 | 0.1783 | 1.7974 |
| $r_s$ | 1.0136(15) | 0.2288(66) | 1.7563(9) |
| $r_0$[e] | 1.044(24) | 0.324(60) | 1.758(18) |

[a]MP2/aVDZ calculation, converted to principal axes.
[b]Coordinate signs assigned for consistency with the least-squares fit.
[c]Fit of 5 parameters to 6 rotational constants, $\sigma_{\text{fit}}$=0.050 uÅ$^2$.
[d]Fit of 7 parameters to 9 rotational constants, $\sigma_{\text{fit}}$=0.139 uÅ$^2$.
[e]Fit of 9 parameters to 12 rotational constants, $\sigma_{\text{fit}}$=0.135 uÅ$^2$.

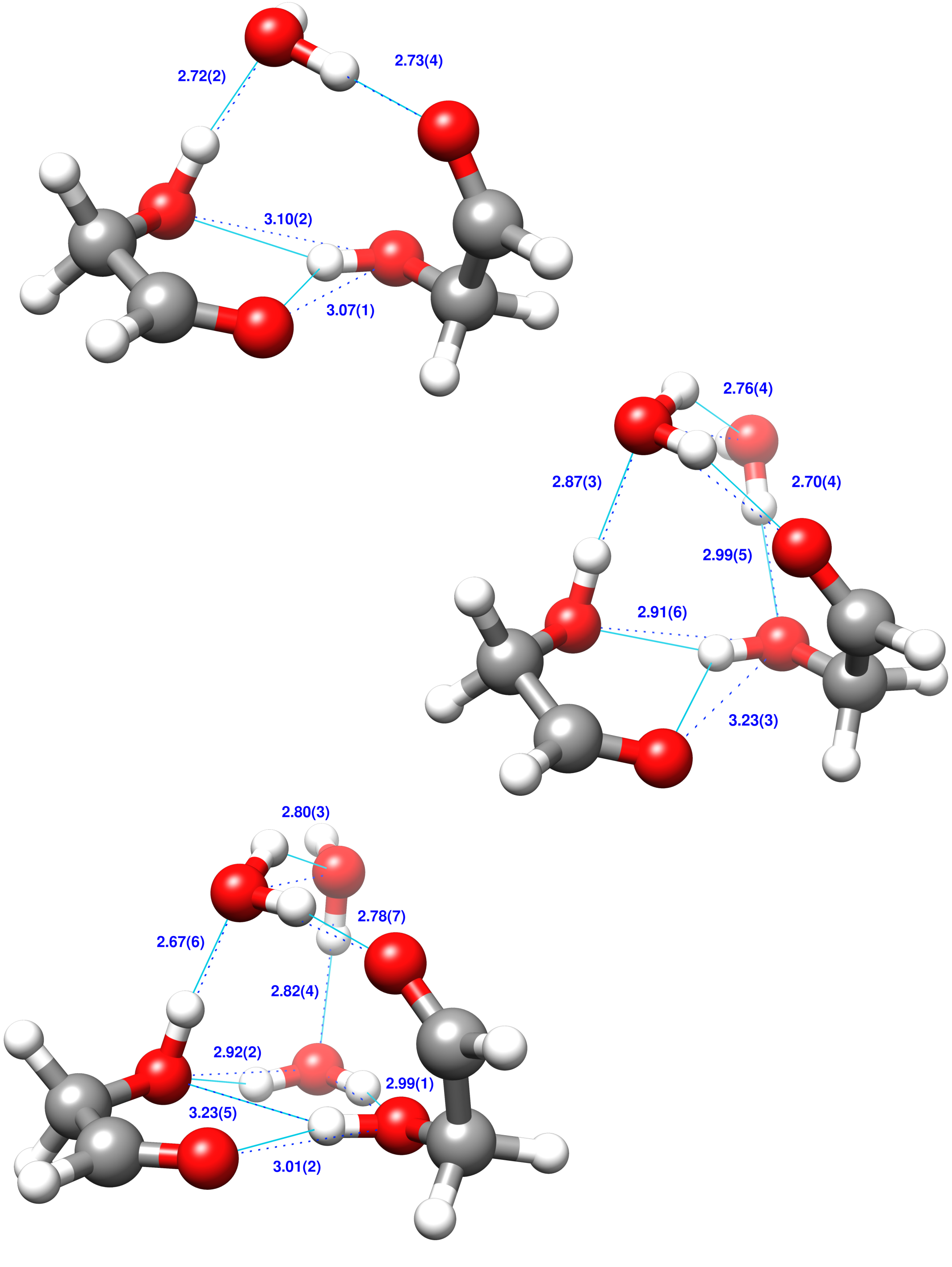


Figure S1: Preferred partial $r_0$ geometries determined for the $Gly_2$-$(H_2O)_n$ clusters. The experimental O-O distances are shown in Angstrom.

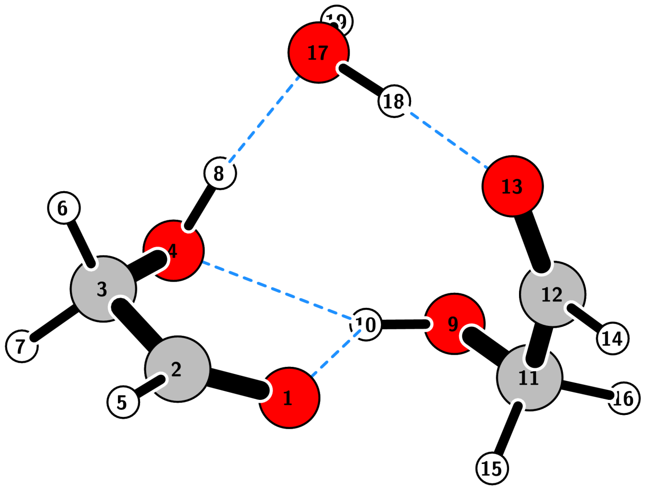


Figure S2: Atom numbering used in the least-squares fit of the $Gly_2$-$H_2O$ structure.

Table S14: The abbreviated results of fitting the partial $r_0$ geometry of the $Gly_2$-$H_2O$ cluster with the STRFIT program.

```
(Gly)2+(H2O) based on reordered unscaled coordinates
```

```
!
!  calculation = EFP-2Gly1W-99-mp2.log Energy: -333624.5698086
!
NUMBER OF ATOMS = 19

  NO  NA  NB  NC       NO.NA      NO.NA.NB   NO.NA.NB.NC        MASS

   1   0   0   0     0.000000     0.000000     0.000000     15.9949150
   2   1   0   0     1.225771     0.000000     0.000000     12.0000000
   3   2   1   0     1.515335   124.173344     0.000000     12.0000000
   4   3   2   1     1.410089   113.873492     1.033896     15.9949150
   5   2   3   4     1.116396   115.031521  -178.747889      1.0078250
   6   3   2   1     1.108259   107.266353  -124.390312      1.0078250
   7   3   2   1     1.105728   107.079066   121.544985      1.0078250
   8   4   3   2     0.988475   107.129733   -84.720955      1.0078250
   9   1   2   3     3.111876   119.675010    -1.564350     15.9949150
  10   9   1   2     0.977403    39.075019   -11.788385      1.0078250
  11   9   1   2     1.405789    69.940328   177.249664     12.0000000
  12  11   9  10     1.512013   115.345934   -75.319556     12.0000000
  13  12  11   9     1.227340   125.834337    -0.680985     15.9949150
  14  12  11   9     1.115500   114.385345  -179.853571      1.0078250
  15  11  12  13     1.106931   106.509543  -127.495734      1.0078250
  16  11  12  13     1.106174   106.328094   118.999351      1.0078250
  17   4   3   2     2.698986   104.263050   -79.008554     15.9949150
  18  17   4   3     0.982487    97.555426    92.376855      1.0078250
  19  17  18  13     0.966024   104.399461   166.071938      1.0078250
!
---------------------------------------------
TOTAL NUMBER OF STRUCTURAL PARAMETERS:     5
---------------------------------------------

Parameters to be fitted:
          R( 9, 1) =     2.500000
          R(17, 4) =     2.698986
       A( 9, 1, 2) =   119.675010
       A(17, 4, 3) =   104.263050
    D(11, 9, 1, 2) =   177.249664
!------------------------------------------------------------------------
!  Rotational constants for the parent + the H218O species:
```

```
TOTAL NUMBER OF SPECTROSCOPIC CONSTANTS:     6

Isotopic    B-expt     Ib-expt
species
---------------------------------
     A    1707.45161  295.98438
     B     998.95996  505.90517
     C     862.07586  586.23496
 2   A    1645.08080  307.20619
     B     998.17130  506.30489
     C     846.11980  597.29014

-------------------------------------------------------
DEFINITIONS OF SUBSTITUTED ISOTOPIC SPECIES
-------------------------------------------------------
!
!                  18.O1 = atom 17
!
ISOTOPIC SPECIES  2,  changes from parent species:
atom no.,parameter no.,value    17  4       17.9991315

-------------------------------------------------------
after:   7 iterations,  ALAMDA=  0.10E-09

FINAL RESULTS OF LEAST SQUARES FIT:
                                                     starting

          R( 9, 1) =     3.072613 +-  0.014533        3.1118759
          R(17, 4) =     2.719390 +-  0.019971        2.6989855
       A( 9, 1, 2) =   125.119651 +-  0.368321      119.6750097
       A(17, 4, 3) =   106.208201 +-  1.067356      104.2630496
    D(11, 9, 1, 2) =   170.571497 +-  0.894148      177.2496637

              Chi-squared =        0.0025005341
         Deviation of fit =        0.050005

Ni Axis     Iobs       Icalc     Io-c            Bobs       Bcalc     Bo-c

 1  a    295.98438   296.02024  -0.03585        1707.4516   1707.2448    0.2068
 1  b    505.90517   505.90221   0.00296         998.9600    998.9658   -0.0058
 1  c    586.23496   586.22902   0.00594         862.0759    862.0846   -0.0087
 2  a    307.20619   307.17264   0.03356        1645.0808   1645.2605   -0.1797
 2  b    506.30489   506.30741  -0.00252         998.1713    998.1663    0.0050
 2  c    597.29014   597.29635  -0.00621         846.1198    846.1110    0.0088

Correlation coefficients:

                          1      2      3      4      5

1:          R( 9, 1)   1.000
2:          R(17, 4)  -0.097  1.000
3:       A( 9, 1, 2)  -0.994  0.207  1.000
4:       A(17, 4, 3)   0.490 -0.915 -0.584  1.000
5:    D(11, 9, 1, 2)  -0.045 -0.989 -0.067  0.847  1.000

Principal coordinates and estimated uncertainties:

ATOM NO.      A       dA           B       dB           C        dC

  1       0.58833  0.02496     -1.47670  0.01678      0.54735  0.04071
  2       1.76727  0.02205     -1.22076  0.03395      0.76442  0.03366
  3       2.55336  0.00273     -0.14858  0.02669      0.03728  0.00545
  4       1.81106  0.01215      0.53360  0.02964     -0.94861  0.02571
  5       2.34294  0.03410     -1.78820  0.06463      1.53446  0.05392
  6       2.97036  0.00640      0.53202  0.05024      0.80613  0.02490
  7       3.41541  0.00816     -0.64738  0.03594     -0.44304  0.01942
  8       1.31818  0.02125      1.25872  0.01559     -0.49217  0.05100
  9      -1.15829  0.00188     -0.09924  0.07450     -1.57229  0.00333
 10      -0.22455  0.00146     -0.18190  0.05633     -1.29550  0.00190
 11      -1.95422  0.00815     -0.90982  0.03967     -0.74422  0.03706
 12      -2.19689  0.00579     -0.37194  0.03144      0.64790  0.01140
 13      -1.77423  0.00318      0.69171  0.05281      1.09103  0.04077
```

| | | | | | | |
|---|---|---|---|---|---|---|
| 14 | -2.83220 | 0.01301 | -1.02629 | 0.05948 | 1.29020 | 0.04397 |
| 15 | -1.55769 | 0.01777 | -1.93584 | 0.03124 | -0.62032 | 0.08620 |
| 16 | -2.94927 | 0.00820 | -1.00890 | 0.06833 | -1.21717 | 0.04330 |
| 17 | 0.27703 | 0.03728 | 2.34702 | 0.00635 | 0.37552 | 0.03665 |
| 18 | -0.52257 | 0.02841 | 1.78624 | 0.01360 | 0.48247 | 0.01718 |
| 19 | 0.08534 | 0.03972 | 2.89380 | 0.03600 | -0.39746 | 0.06359 |

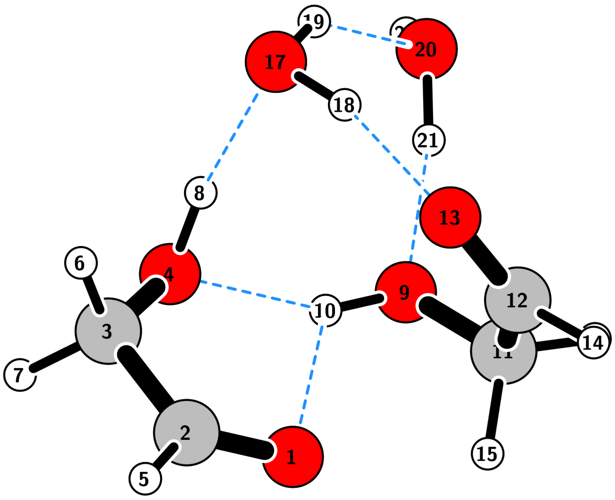


Figure S3: Atom numbering used in the least-squares fit of the $Gly_2$-$(H_2O)_2$ structure.

Table S15: The abbreviated results of fitting the partial $r_0$ geometry of the $Gly_2$-$(H_2O)_2$ cluster with the STRFIT program.

---

```
(Gly)2-(H2O)2 based on reordered unscaled coordinates
```

---

```
!
!  calculation = hf3c-87-1285-mp2.log Energy: -381346.9241904
!
!  glycolaldehyde: 1/ B3LYP/6311++G(d,p) calculation, which reproduces A0,B0,C0
!                        (with nominal Cs equilibrium structure)
!                     2/ dihedral D(OHCC) from calculation
!
!  H2O: r0 OH and HOH from Table VI, F.C.De Lucia et al., Phys.Rev.A 8,2785(1973)
!

NUMBER OF ATOMS = 22
```

| NO | NA | NB | NC | NO.NA | NO.NA.NB | NO.NA.NB.NC | MASS |
|---|---|---|---|---|---|---|---|
| 1 | 0 | 0 | 0 | 0.000000 | 0.000000 | 0.000000 | 15.9949150 |
| 2 | 1 | 0 | 0 | 1.206800 | 0.000000 | 0.000000 | 12.0000000 |
| 3 | 2 | 1 | 0 | 1.504100 | 122.087000 | 0.000000 | 12.0000000 |
| 4 | 3 | 2 | 1 | 1.400500 | 112.605000 | 2.307751 | 15.9949150 |
| 5 | 2 | 3 | 4 | 1.107100 | 116.413000 | 177.286630 | 1.0078250 |
| 6 | 3 | 2 | 1 | 1.100500 | 107.832000 | -122.740000 | 1.0078250 |
| 7 | 3 | 2 | 1 | 1.100500 | 107.832000 | 122.740000 | 1.0078250 |
| 8 | 4 | 3 | 2 | 0.970600 | 106.709000 | -102.920980 | 1.0078250 |
| 9 | 1 | 2 | 3 | 3.090733 | 117.523014 | 3.239962 | 15.9949150 |
| 10 | 9 | 1 | 2 | 0.970600 | 39.190517 | -13.166577 | 1.0078250 |
| 11 | 9 | 1 | 2 | 1.400500 | 70.455288 | 158.993154 | 12.0000000 |
| 12 | 11 | 9 | 10 | 1.504100 | 112.605000 | -63.913761 | 12.0000000 |
| 13 | 12 | 11 | 9 | 1.206800 | 122.087000 | 0.000000 | 15.9949150 |
| 14 | 12 | 11 | 9 | 1.107100 | 116.413000 | -180.000000 | 1.0078250 |
| 15 | 11 | 12 | 13 | 1.100500 | 107.832000 | -122.740000 | 1.0078250 |
| 16 | 11 | 12 | 13 | 1.100500 | 107.832000 | 122.740000 | 1.0078250 |
| 17 | 4 | 3 | 2 | 2.665982 | 107.182264 | -97.093055 | 15.9949150 |
| 18 | 17 | 4 | 3 | 0.965000 | 94.321478 | 76.164632 | 1.0078250 |
| 19 | 17 | 18 | 13 | 0.965000 | 104.800000 | -80.264724 | 1.0078250 |
| 20 | 17 | 4 | 3 | 2.808333 | 89.916366 | 164.547053 | 15.9949150 |
| 21 | 20 | 17 | 4 | 0.965000 | 84.349728 | -36.305510 | 1.0078250 |
| 22 | 20 | 21 | 17 | 0.965000 | 104.800000 | -132.079262 | 1.0078250 |

---------------------------------------------

```
TOTAL NUMBER OF STRUCTURAL PARAMETERS:     7
---------------------------------------------

Parameters to be fitted:
          R( 9, 1) =     4.400000
          R(17, 4) =     4.000000
          R(20,17) =     2.808333
       A( 9, 1, 2) =   117.523014
       A(17, 4, 3) =   107.182264
       A(20,17, 4) =    89.916366
    D(20,17, 4, 3) =   164.547053
!
!---------------------------------------------------------------------
!  Rotational constants for the parent + the two single H218O

TOTAL NUMBER OF SPECTROSCOPIC CONSTANTS:     9

Isotopic    B-expt     Ib-expt
species
---------------------------------
     A    1160.01937  435.66429
     B     860.74300  587.14275
     C     659.75702  766.00778
 2   A    1151.52770  438.87699
     B     835.10608  605.16744
     C     643.11862  785.82550
 3   A    1134.78530  445.35209
     B     853.54700  592.09277
     C     652.54130  774.47820
---------------------------------


-------------------------------------------------------
DEFINITIONS OF SUBSTITUTED ISOTOPIC SPECIES
-------------------------------------------------------
!
!                  18.O2 = atom 20
!
ISOTOPIC SPECIES  2,  changes from parent species:
atom no.,parameter no.,value    20  4       17.9991315
!
!                  18.O1 = atom 17
!
ISOTOPIC SPECIES  3,  changes from parent species:
atom no.,parameter no.,value    17  4       17.9991315

-------------------------------------------------------
after:   8 iterations,  ALAMDA=  0.10E-10

FINAL RESULTS OF LEAST SQUARES FIT:
                                                   starting

          R( 9, 1) =     3.231877 +-  0.031513        3.0907334
          R(17, 4) =     2.869521 +-  0.029824        2.6659815
          R(20,17) =     2.763910 +-  0.050864        2.8083333
       A( 9, 1, 2) =   121.474682 +-  0.930719      117.5230136
       A(17, 4, 3) =   103.935113 +-  2.132367      107.1822636
       A(20,17, 4) =    86.374626 +-  0.360006       89.9163659
    D(20,17, 4, 3) =   172.881817 +-  2.027098     -164.7965060

              Chi-squared =        0.0389045432
         Deviation of fit =        0.139471

Ni Axis     Iobs       Icalc     Io-c            Bobs       Bcalc     Bo-c

 1  a    435.66429   435.60895   0.05533        1160.0194   1160.1667   -0.1473
 1  b    587.14275   587.14875  -0.00600         860.7430    860.7342    0.0088
 1  c    766.00778   765.86826   0.13952         659.7570    659.8772   -0.1202
 2  a    438.87699   438.92086  -0.04387        1151.5277   1151.4126    0.1151
 2  b    605.16744   605.11962   0.04782         835.1061    835.1721   -0.0660
 2  c    785.82550   785.91498  -0.08948         643.1186    643.0454    0.0732
 3  a    445.35209   445.36123  -0.00914        1134.7853   1134.7620    0.0233
 3  b    592.09277   592.13345  -0.04068         853.5470    853.4884    0.0586
```

| | | | | | | | |
|---|---|---|---|---|---|---|---|
| 3 | c | 774.47820 | 774.52705 | -0.04886 | 652.5413 | 652.5001 | 0.0412 |

Correlation coefficients:

| | | 1 | 2 | 3 | 4 | 5 | 6 | 7 |
|---|---|---|---|---|---|---|---|---|
| 1: | R( 9, 1) | 1.000 | | | | | | |
| 2: | R(17, 4) | 0.711 | 1.000 | | | | | |
| 3: | R(20,17) | -0.870 | -0.940 | 1.000 | | | | |
| 4: | A( 9, 1, 2) | -0.946 | -0.864 | 0.950 | 1.000 | | | |
| 5: | A(17, 4, 3) | -0.906 | -0.822 | 0.858 | 0.960 | 1.000 | | |
| 6: | A(20,17, 4) | 0.794 | 0.779 | -0.788 | -0.906 | -0.961 | 1.000 | |
| 7: | D(20,17, 4, 3) | 0.953 | 0.813 | -0.943 | -0.989 | -0.916 | 0.856 | 1.000 |

Principal coordinates and estimated uncertainties:

| ATOM NO. | A | dA | B | dB | C | dC |
|---|---|---|---|---|---|---|
| 1 | -2.06436 | 0.03301 | -0.95049 | 0.02610 | -0.05139 | 0.06869 |
| 2 | -2.69128 | 0.00398 | 0.06032 | 0.04882 | 0.15259 | 0.04759 |
| 3 | -2.15939 | 0.02954 | 1.41832 | 0.02478 | -0.21517 | 0.01992 |
| 4 | -0.91246 | 0.01545 | 1.35437 | 0.04294 | -0.84958 | 0.05168 |
| 5 | -3.65344 | 0.01631 | 0.03991 | 0.09386 | 0.69986 | 0.07517 |
| 6 | -2.14151 | 0.07009 | 2.03193 | 0.05179 | 0.69821 | 0.04366 |
| 7 | -2.88497 | 0.02743 | 1.89205 | 0.03071 | -0.89356 | 0.01845 |
| 8 | -0.24906 | 0.03539 | 1.60531 | 0.03412 | -0.18702 | 0.07969 |
| 9 | 0.92291 | 0.04171 | -0.86409 | 0.05371 | -1.28174 | 0.03040 |
| 10 | 0.14879 | 0.03065 | -0.29472 | 0.04066 | -1.14528 | 0.02660 |
| 11 | 0.72252 | 0.04181 | -2.08376 | 0.03053 | -0.62322 | 0.01582 |
| 12 | 0.61946 | 0.02542 | -1.92505 | 0.02120 | 0.86892 | 0.01363 |
| 13 | 0.70485 | 0.04046 | -0.84795 | 0.03738 | 1.40645 | 0.02470 |
| 14 | 0.46033 | 0.03561 | -2.85201 | 0.04082 | 1.45295 | 0.04767 |
| 15 | -0.19808 | 0.05716 | -2.57396 | 0.03164 | -0.97437 | 0.05732 |
| 16 | 1.54599 | 0.05662 | -2.78344 | 0.04738 | -0.83163 | 0.01595 |
| 17 | 0.96489 | 0.04034 | 1.83784 | 0.02057 | 1.26606 | 0.03415 |
| 18 | 0.87980 | 0.03120 | 0.95331 | 0.02829 | 1.64233 | 0.00368 |
| 19 | 1.89572 | 0.04688 | 1.91389 | 0.03444 | 1.02315 | 0.04777 |
| 20 | 2.93212 | 0.01446 | 1.22964 | 0.02338 | -0.57766 | 0.02375 |
| 21 | 2.29388 | 0.01486 | 0.58306 | 0.02022 | -0.90294 | 0.02789 |
| 22 | 3.24204 | 0.01803 | 1.68906 | 0.02953 | -1.36766 | 0.02075 |

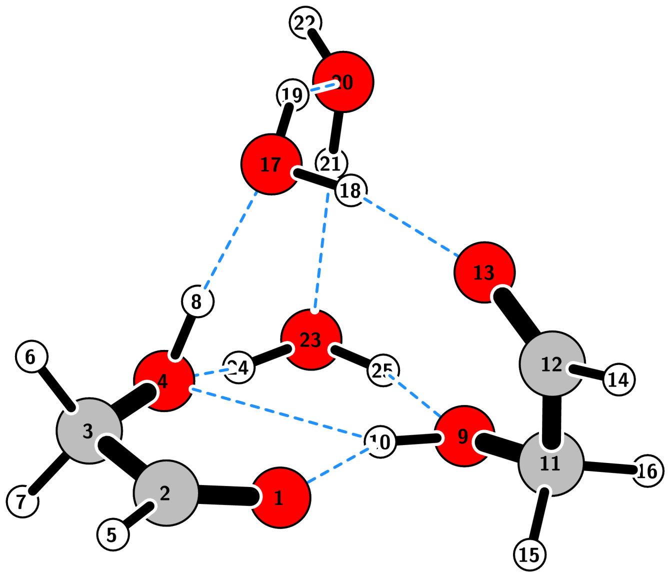


Figure S4: Atom numbering used in the least-squares fit of the $Gly_2$-$(H_2O)_3$ structure.

Table S16: The abbreviated results of fitting the partial $r_0$ geometry of the $Gly_2$-$(H_2O)_3$ cluster with the STRFIT program.

```
(Gly)2-(H2O)3 based on Reordered unscaled coordinates from ORCA-job ofinal-22

!
!  glycolaldehyde: 1/ B3LYP/6311++G(d,p) calculation, which reproduces A0,B0,C0
!                        (with nominal Cs equilibrium structure)
!                    2/ Dihedral HOCC in the cluster from the ORCA calculation
!
!  H2O: r0 OH and HOH from Table VI, F.C.De Lucia et al., Phys.Rev.A 8,2785(1973)
!
!
NUMBER OF ATOMS = 25

  NO  NA  NB  NC       NO.NA      NO.NA.NB   NO.NA.NB.NC        MASS

   1   0   0   0     0.000000     0.000000     0.000000     15.9949150
   2   1   0   0     1.206800     0.000000     0.000000     12.0000000
   3   2   1   0     1.504100   122.087000     0.000000     12.0000000
   4   3   2   1     1.400500   112.605000     0.000000     15.9949150
   5   2   3   4     1.107100   116.413000   180.000000      1.0078250
   6   3   2   1     1.100500   107.832000  -122.740000      1.0078250
   7   3   2   1     1.100500   107.832000   122.740000      1.0078250
   8   4   3   2     0.970600   106.709000   -78.804145      1.0078250
   9   1   2   3     2.885196   124.791751    -6.361413     15.9949150
  10   9   1   2     0.970600    26.239540     3.071319      1.0078250
  11   9   1   2     1.400500    81.317276   175.540215     12.0000000
  12  11   9  10     1.504100   112.605000   -66.685828     12.0000000
  13  12  11   9     1.206800   122.087000     0.000000     15.9949150
  14  12  11   9     1.107100   116.413000  -180.000000      1.0078250
  15  11  12  13     1.100500   107.832000  -122.740000      1.0078250
  16  11  12  13     1.100500   107.832000   122.740000      1.0078250
  17   4   3   2     2.624140   106.615430   -76.789018     15.9949150
  18  17   4   3     0.965000   103.842613    90.606955      1.0078250
  19  17  18  13     0.965000   104.800000  -154.128869      1.0078250
  20  17   4   3     2.765306    93.540045  -164.796506     15.9949150
  21  20  17   4     0.965000    94.451031   -15.300227      1.0078250
  22  20  21  17     0.965000   104.800000  -135.747573      1.0078250
  23   4   3   2     2.893638   166.770077    90.546181     15.9949150
```

```
  24  23   4   3     0.965000    25.075218    46.939899      1.0078250
  25  23  24   4     0.965000   104.800000    45.114705      1.0078250

---------------------------------------------
TOTAL NUMBER OF STRUCTURAL PARAMETERS:     9
---------------------------------------------

Parameters to be fitted:
          R( 9, 1) =     4.400000
          R(17, 4) =     3.000000
          R(20,17) =     2.765306
          R(23, 4) =     2.893638
       A( 9, 1, 2) =   124.791751
       A(17, 4, 3) =   106.615430
       A(20,17, 4) =    93.540045
       A(23, 4, 3) =   166.770077
    D( 9, 1, 2, 3) =    -6.361413
!
!------------------------------------------------------------------------
!  Rotational constants for the parent + the three single H218O
!

TOTAL NUMBER OF SPECTROSCOPIC CONSTANTS:    12

Isotopic    B-expt     Ib-expt
species
---------------------------------
     A     789.95455  639.75707
     B     673.86436  749.97141
     C     507.12204  996.56289
 2   A     782.41149  645.92483
     B     659.50109  766.30504
     C     501.73441 1007.26400
 3   A     788.71724  640.76070
     B     651.84401  775.30667
     C     494.86097 1021.25454
 4   A     782.39834  645.93569
     B     666.62252  758.11872
     C     506.01645  998.74028
---------------------------------

-------------------------------------------------------
DEFINITIONS OF SUBSTITUTED ISOTOPIC SPECIES
-------------------------------------------------------
!
!                  18.01 = atom 23
!
ISOTOPIC SPECIES  2,  changes from parent species:
atom no.,parameter no.,value    23  4       17.9991315
!
!                  18.02 = atom 20
!
ISOTOPIC SPECIES  3,  changes from parent species:
atom no.,parameter no.,value    20  4       17.9991315
!
!                  18.03 = atom 17
!
ISOTOPIC SPECIES  4,  changes from parent species:
atom no.,parameter no.,value    17  4       17.9991315

------------------------------------------------------------------------------
after:   9 iterations,  ALAMDA=  0.10E-11

FINAL RESULTS OF LEAST SQUARES FIT:
                                                            starting

          R( 9, 1) =     3.009999 +-  0.023762              2.885196
          R(17, 4) =     2.671617 +-  0.066266              2.624139
          R(20,17) =     2.798180 +-  0.032536              2.765306
          R(23, 4) =     2.925433 +-  0.023668              2.893638
       A( 9, 1, 2) =   131.780731 +-  0.446144            124.791751
       A(17, 4, 3) =   110.643849 +-  3.236085            106.615430
       A(20,17, 4) =    96.902931 +-  1.196389             93.540044
```

```
     A(23, 4, 3) =   165.624191 +-  4.757496            166.770076
  D( 9, 1, 2, 3) =   -10.334412 +-  3.573743             -6.361413

            Chi-squared =        0.0549050636
       Deviation of fit =        0.135284
```

| Ni | Axis | Iobs | Icalc | Io-c | Bobs | Bcalc | Bo-c |
|---|---|---|---|---|---|---|---|
| 1 | a | 639.75707 | 639.70320 | 0.05388 | 789.9545 | 790.0211 | -0.0665 |
| 1 | b | 749.97141 | 749.86460 | 0.10681 | 673.8644 | 673.9603 | -0.0960 |
| 1 | c | 996.56289 | 996.41033 | 0.15256 | 507.1220 | 507.1997 | -0.0776 |
| 2 | a | 645.92483 | 645.94824 | -0.02341 | 782.4115 | 782.3831 | 0.0284 |
| 2 | b | 766.30504 | 766.36107 | -0.05603 | 659.5011 | 659.4529 | 0.0482 |
| 2 | c | 1007.26400 | 1007.29372 | -0.02973 | 501.7344 | 501.7196 | 0.0148 |
| 3 | a | 640.76070 | 640.73639 | 0.02431 | 788.7172 | 788.7472 | -0.0299 |
| 3 | b | 775.30667 | 775.32682 | -0.02015 | 651.8440 | 651.8271 | 0.0169 |
| 3 | c | 1021.25454 | 1021.29902 | -0.04448 | 494.8610 | 494.8394 | 0.0216 |
| 4 | a | 645.93569 | 645.98976 | -0.05407 | 782.3983 | 782.3329 | 0.0655 |
| 4 | b | 758.11872 | 758.14241 | -0.02370 | 666.6225 | 666.6017 | 0.0208 |
| 4 | c | 998.74028 | 998.81957 | -0.07929 | 506.0164 | 505.9763 | 0.0402 |

Correlation coefficients:

| | | 1 | 2 | 3 | 4 | 5 | 6 | 7 | 8 | 9 |
|---|---|---|---|---|---|---|---|---|---|---|
| 1: | R( 9, 1) | 1.000 | | | | | | | | |
| 2: | R(17, 4) | -0.857 | 1.000 | | | | | | | |
| 3: | R(20,17) | 0.603 | -0.724 | 1.000 | | | | | | |
| 4: | R(23, 4) | 0.774 | -0.657 | 0.384 | 1.000 | | | | | |
| 5: | A( 9, 1, 2) | 0.018 | -0.400 | 0.270 | 0.158 | 1.000 | | | | |
| 6: | A(17, 4, 3) | 0.913 | -0.887 | 0.510 | 0.544 | 0.101 | 1.000 | | | |
| 7: | A(20,17, 4) | -0.752 | 0.729 | -0.814 | -0.353 | -0.184 | -0.752 | 1.000 | | |
| 8: | A(23, 4, 3) | -0.882 | 0.895 | -0.576 | -0.474 | -0.093 | -0.990 | 0.786 | 1.000 | |
| 9: | D( 9, 1, 2, 3) | -0.862 | 0.953 | -0.655 | -0.709 | -0.517 | -0.855 | 0.737 | 0.831 | 1.000 |

Principal coordinates and estimated uncertainties:

| ATOM NO. | A | dA | B | dB | C | dC |
|---|---|---|---|---|---|---|
| 1 | -2.21357 | 0.02237 | 0.67199 | 0.05657 | -0.08141 | 0.03307 |
| 2 | -2.16586 | 0.04942 | 1.84944 | 0.05859 | 0.17878 | 0.05636 |
| 3 | -0.88240 | 0.07268 | 2.63090 | 0.01870 | 0.11283 | 0.03767 |
| 4 | 0.20834 | 0.05901 | 1.84285 | 0.02746 | -0.27534 | 0.06842 |
| 5 | -3.07030 | 0.06096 | 2.41241 | 0.09163 | 0.47996 | 0.11506 |
| 6 | -0.71051 | 0.07524 | 3.08075 | 0.03789 | 1.10237 | 0.04861 |
| 7 | -1.03252 | 0.11963 | 3.46706 | 0.02616 | -0.58674 | 0.06540 |
| 8 | 0.48399 | 0.04006 | 1.34207 | 0.02125 | 0.50907 | 0.09078 |
| 9 | -0.19722 | 0.05659 | -1.19317 | 0.04408 | -1.31251 | 0.02006 |
| 10 | -0.49303 | 0.03368 | -0.33525 | 0.03198 | -0.96823 | 0.03470 |
| 11 | -1.19985 | 0.06877 | -2.12709 | 0.03869 | -1.02281 | 0.04491 |
| 12 | -1.35176 | 0.06747 | -2.37313 | 0.03710 | 0.45323 | 0.05768 |
| 13 | -0.66660 | 0.07046 | -1.80907 | 0.04344 | 1.27101 | 0.01790 |
| 14 | -2.12872 | 0.08994 | -3.10238 | 0.06294 | 0.75356 | 0.10693 |
| 15 | -2.17373 | 0.07063 | -1.79198 | 0.06283 | -1.41059 | 0.07050 |
| 16 | -0.98902 | 0.10206 | -3.09332 | 0.04944 | -1.50558 | 0.06835 |
| 17 | 1.04377 | 0.02380 | 0.32393 | 0.05956 | 1.75751 | 0.01813 |
| 18 | 0.53662 | 0.02824 | -0.49127 | 0.05340 | 1.66025 | 0.01632 |
| 19 | 1.95354 | 0.03333 | 0.03328 | 0.07097 | 1.89558 | 0.16101 |
| 20 | 3.52012 | 0.00732 | -0.37616 | 0.07887 | 0.65870 | 0.03110 |
| 21 | 3.28773 | 0.01143 | -0.09516 | 0.07057 | -0.23476 | 0.02886 |
| 22 | 4.41115 | 0.00786 | -0.03397 | 0.09822 | 0.80081 | 0.04046 |
| 23 | 2.28683 | 0.01230 | 0.41300 | 0.07107 | -1.75640 | 0.01679 |
| 24 | 1.74377 | 0.02724 | 1.17810 | 0.05966 | -1.53072 | 0.02327 |
| 25 | 1.66585 | 0.02197 | -0.32397 | 0.06155 | -1.80624 | 0.03268 |

# 2 Computational Methodology

## 2.1 Structure Determination

### 2.1.1 Genetic Algorithm Search on Semi-empirical PES

Determining the structure of the most stable molecular clusters is challenging because it requires that

- one has an accurate and inexpensive way to generate the potential energy surface (PES), which is especially challenging for weakly bound molecular clusters, and
- an efficient algorithm to navigate this PES which is generally very flat with many shallow minima.

To overcome these two challenges, we employed a genetic algorithm (GA)[S4] approach on semi-empirical PES. The GA approach is implemented in the OGOLEM[S5] package which interfaces with the PM7[S6] method implemented in MOPAC[S7] and HF-3c[S8] method implemented in ORCA.[S9] Additionally, another GA package named CLUSTER[S10] was interfaced with with the Effective Fragment Potential (EFP2)[S11] method to do further searches of the configurational space. The details of the GA runs are summarized in Table S17

Table S17: Details of GA runs for $(Gly)_2W_n$, where $n = 0 - 3$

| GA package | SE package | Method | Pool size$^a$ | Number of cycles$^b$ |
|---|---|---|---|---|
| OGOLEM | MOPAC | PM7 | 250-500 | 10,000 - 20,000 |
| OGOLEM | ORCA | HF-3c | 250-500 | 2,000 - 5,000 |
| CLUSTER | libEFP | EFP2 | 100-250 | 5,000 |

$^a$ The size of the initial and/or maintained population.
$^b$ Number of matings or cycles for which the population is evolved.

GA searches on semi-empirical (PM7, HF-3c, EFP2) PESs yield a large number of stable isomers which are subsequently subject to refinements using more robust *ab initio* methods.

### 2.1.2 Refinements using *ab initio* Methods

The low energy isomers from the previous step that are within 5 kcal mol$^{-1}$ of the putative global minimum for each method (PM7, HF-3c, EFP2) are first optimized using second-order Møller-Plesset perturbation theory (MP2)[S12] with a split-valence 6-31+G(d) basis set. Those isomers that are within 3 kcal mol$^{-1}$ of the MP2/6-31+G(d) global minimum are further optimized using the larger double-zeta basis set (aug-cc-pVDZ or aVDZ).[S13] For each isomer which is within 2 kcal mol$^{-1}$ of the MP2/aVDZ global minimum, we do further geometric optimizations using very tight convergence criteria and calculate the harmonic vibrational calculations using analytic Hessians as implemented in Gaussian 16 Rev B.01.[S14]

The MP2 complete basis set limit (MP2/CBS) energy is estimated using explicitly correlated MP2-F12 method with cc-pVTZ-F12 orbital basis set along with cc-pVTZ-F12-CABS Complementary Auxiliary Basis Set (CABS) and cc-pVQZ/C auxiliary basis at the MP2/aVDZ optimized geometry. These methods are implemented in ORCA 4.2.1.[S9,S15] This method should fully be described as RI-MP2-F12/cc-pVTZ-F12//RI-MP2/aug-cc-pVDZ, however we will abbreviate it as MP2-F12/VTZ-F12//MP2/aVDZ in the rest of this manuscript for the sake of brevity.

These MP2-F12/VTZ-F12//MP2/aVDZ electronic energy ($E_e$) estimates are combined with the MP2/aVDZ harmonic zero-point and thermodynamic corrections to get zero-point corrected energies ($E_0$) and Gibbs free energies at finite temperatures [G(T)].

## 2.2 Rotational Constants, Principal Dipole Moments and Energies

### 2.2.1 Rotational Constants, Principal Dipole Moments and Relative Energies

The equilibrium theoretical rotational constants (A, B, C) and the principal dipole moment components ($\mu_a$, $\mu_b$, $\mu_c$) are reported in Table S18. The relative energies ($\Delta\Delta E_e$ $\Delta\Delta$E(0k), $\Delta\Delta$G ) for a cluster $k$ are defined as

$$\Delta\Delta E_k = E_k - \min_{i=0,\ldots n} E_i \quad (1)$$

Table S18: The MP2/aVDZ rotational constants, principal dipole moments and MP2-F12/VTZ-F12//MP2/aVDZ relative energies of low energy isomers of $(Gly)_2W_n$, where $n = 0 - 3$

| | Rot. Constants (MHz) | | | Prin. Dipole Moment (Debye) | | | | Relative Energy[b] (kJ mol$^{-1}$) | | |
|---|---|---|---|---|---|---|---|---|---|---|
| Label[a] | A | B | C | $\mu_a$ | $\mu_b$ | $\mu_c$ | $\mu$-type | $\Delta\Delta E_e$ | $\Delta\Delta$E(0k) | $\Delta\Delta$G(100k) |
| $Gly_2W_0$- | | | | | | | | | | |
| I | 2757 | 1322 | 1260 | 0.00 | 0.00 | -3.61 | c | 0.0 | 0.0 | 0.0 |
| II | 2841 | 1265 | 1016 | -0.02 | 1.04 | -0.16 | b | 4.6 | 4.3 | 3.6 |
| $Gly_2W_1$- | | | | | | | | | | |
| I | 1712 | 1025 | 901 | -0.56 | -1.45 | -0.97 | b | 0.0 | 0.0 | 0.0 |
| II | 1498 | 1285 | 968 | 1.19 | -0.34 | -1.19 | | 5.0 | 3.5 | 4.1 |
| III | 2122 | 844 | 763 | -2.19 | -0.31 | 2.39 | | 4.1 | 3.7 | 3.7 |
| $Gly_2W_2$- | | | | | | | | | | |
| I | 1183 | 879 | 675 | -1.99 | 1.64 | -1.10 | | 0.0 | 0.0 | 0.0 |
| II | 1132 | 953 | 657 | 1.18 | 0.50 | 1.06 | | 2.2 | 1.4 | 1.7 |
| III | 1240 | 820 | 656 | -2.60 | 1.27 | -1.84 | a | 3.4 | 2.7 | 2.5 |
| $Gly_2W_3$- | | | | | | | | | | |
| I | 817 | 688 | 525 | -3.02 | 1.26 | 0.79 | a | 0.0 | 0.0 | 0.0 |
| II | 889 | 736 | 520 | 0.47 | -0.51 | -0.45 | | 1.4 | 0.7 | 1.2 |
| III | 985 | 581 | 480 | 2.22 | 1.65 | -1.58 | | 2.1 | 1.6 | 1.2 |

[a] Ordered by relative electronic energy ($\Delta\Delta$E), with the global minimum labeled 'I', followed by 'II', 'III', 'IV' ... *etc* for higher energy isomers

[b] MP2-F12/VTZ-F12//MP2/aVDZ single point energies combined with thermodynamic corrections at MP2/aVDZ level of theory

#### 2.2.2 Binding Energies

The total binding energy of a molecular cluster $i$ ($E^i_{bind}$ or $\Delta E^i$) is often calculated in supermolecular approaches as the difference in energy between the cluster ($E^i$) and the $N$ monomers constituting the cluster($E^j_m$) in their isolated gas phase minimum geometries.

$$E^i_{bind} = \Delta E^i = E^i - \sum_j^N E^j_m \tag{2}$$

Table S19: MP2-F12/VTZ-F12//MP2/aVDZ Binding Energies (ΔE, ΔG) of the low energy isomers of $(Gly)_2W_n$, where $n = 0 - 3$

| | Binding Energy[b] (kJ mol$^{-1}$) | | |
|---|---|---|---|
| Label[a] | $\Delta E_e$ | ΔE(0k) | ΔG(100k) |
| $Gly_2W_0$- | | | |
| I | -33.7 | -27.2 | -12.6 |
| II | -29.1 | -22.9 | -8.9 |
| $Gly_2W_1$- | | | |
| I | -73.1 | -56.4 | -31.5 |
| II | -68.1 | -52.9 | -27.4 |
| III | -69.0 | -52.7 | -27.9 |
| $Gly_2W_2$- | | | |
| I | -120.0 | -92.4 | -56.7 |
| II | -117.8 | -91.0 | -55.0 |
| III | -116.6 | -89.7 | -54.2 |
| $Gly_2W_3$- | | | |
| I | -161.7 | -123.7 | -77.8 |
| II | -160.2 | -123.0 | -76.6 |
| III | -159.6 | -122.2 | -76.6 |

[a] Ordered by relative electronic energy (ΔΔE), with the global minimum labeled 'I', followed by 'II', 'III', 'IV' ... *etc* for higher energy isomers

[b] MP2-F12/VTZ-F12//MP2/aVDZ single point energies combined with thermodynamic corrections at MP2/aVDZ level of theory

## 2.3 Comparison of QM Methods

Modeling non-covalently bonded molecular clusters accurately is a challenge for most density functional (DFT) and *a*b initio methods alike. MP2 has generally been shown to perform well for hydrogen-bonded systems while many density functionals incorporating an empirical dispersion correction such as the DFT-D3 with Becke-Johnson damping[S16] also perform reasonably well.

The current MP2/aVDZ geometries and MP2-F12/VTZ-F12//MP2/aVDZ energies are compared with MP2/6-311++G(d,p) and B3LYP-D3BJ/def2-TZVP to evaluate whether these different methods yield comparable geometries (hence rotational constants) and relative energies. The low energy isomers found at the MP2/aVDZ level of theory are re-optimized at MP2/6-311++G(d,p) and B3LYP-D3BJ/def2-TZVP using ORCA 4.2.1.[S9,S15] Please note that both the MP2/aVDZ and MP2/6-311++G(d,p) use density fitting to speed up the MP2 calculations.

Where experimental structures are available, the rotational constants of each conformer predicted by the different methods are compared against experimental fits by calculating the root-mean-square error (RMSE) which is defined as

$$RMSE = \sqrt{\frac{1}{n}\sum_{i=1}^{n}\left(X_{expt}^{i} - X_{calc}^{i}\right)^{2}} \tag{3}$$

$$RMSE = \sqrt{\frac{(A_{Expt} - A_{Calc})^2 + (B_{Expt} - B_{Calc})^2 + (C_{Expt} - C_{Calc})^2}{3}} \tag{4}$$

As Table S20 demonstrates, B3LYP calculations using two basis sets (def2-TZVP and aVTZ) give rotational constants that differ by as much as 15 MHz depending on the package (Gaussian or Orca) and version (G09 or G16) of computational chemistry program used.

Table S20: The rotational constants (A, B, C in MHz) of the two low energy isomers of $(Gly)_2$ calculated using different DFT methods and basis sets as implemented in different packages.

| | Zinn et al.[S17] | Current work | | Zinn et al.[S17] | Current work |
|---|---|---|---|---|---|
| Method | B3LYP-GD3 | B3LYP-GD3 | B3LYP-D3Zero | B3LYP-GD3 | B3LYP-D3Zero |
| Basis | def2-TZVP | def2-TZVP | def2-TZVP | aVTZ | aVTZ |
| Package | G09RevD.01 | G16RevB.01 | Orca 4.2.1 | G09RevD.01 | Orca 4.2.1 |
| $(Gly)_2$-I | | | | | |
| A | 2781.0 | 2775.4 | 2774.0 | 2783.2 | 2774.9 |
| B | 1286.0 | 1290.3 | 1288.3 | 1283.5 | 1286.7 |
| C | 1176.7 | 1180.3 | 1178.8 | 1166.7 | 1170.0 |
| RMSE[a] | 14 | 15 | 16 | 19 | 19 |
| $(Gly)_2$-II | | | | | |
| A | 2780.7 | 2830.2 | 2825.1 | 2780.7 | 2825.2 |
| B | 1243.9 | 1223.3 | 1222.7 | 1233.2 | 1211.1 |
| C | 1015.6 | 999.0 | 998.2 | 1012.8 | 994.9 |
| RMSE[a] | 105 | 73 | 75 | 102 | 72 |

[a] RMSE relative to experimental fit by Zinn et al.[S17]

In contrast, MP2 structures and energies could be reproduced well using different packages (Gaussian or Orca) and version (G09 or G16 or 4.1.2 or 4.2.1) as shown in the next table.

Table S21: The rotational constants (A, B, C in MHz) of the two low energy isomers of $(Gly)_2$ calculated using MP2 and different basis sets as implemented in different packages.

| | Zinn et al.[S17] | Current work | Zinn et al.[S17] | Current work |
|---|---|---|---|---|
| Method | MP2 | MP2[a] | MP2 | MP2[a] |
| Basis | def2-TZVP | def2-TZVP | aVTZ | aVTZ |
| Package | G09RevD.01[b] | Orca 4.2.1[c] | G09RevD.01[b] | Orca 4.2.1[c] |
| $(Gly)_2$-I | | | | |
| A | 2789.5 | 2789.5 | 2802.0 | 2802.1 |
| B | 1304.3 | 1304.5 | 1322.2 | 1322.4 |
| C | 1221.3 | 1221.5 | 1242.1 | 1242.3 |
| RMSE[d] | 18 | 18 | 34 | 35 |
| $(Gly)_2$-II | | | | |
| A | 2868.9 | 2868.7 | 2909.3 | 2909.8 |
| B | 1251.1 | 1251.8 | 1248.8 | 1249.1 |
| B | 1010.3 | 1010.9 | 1010.0 | 1010.2 |
| RMSE[d] | 67 | 68 | 55 | 55 |

[a] RI-MP2 to be precise, but the RI approximation introduces negligible errors
[b] Also reproduced using G16RevB.01
[c] Also reproduced using Orca 4.1.2
[d] RMSE relative to experimental fit by Zinn et al.[S17]

### 2.3.1 $(Gly)_2$

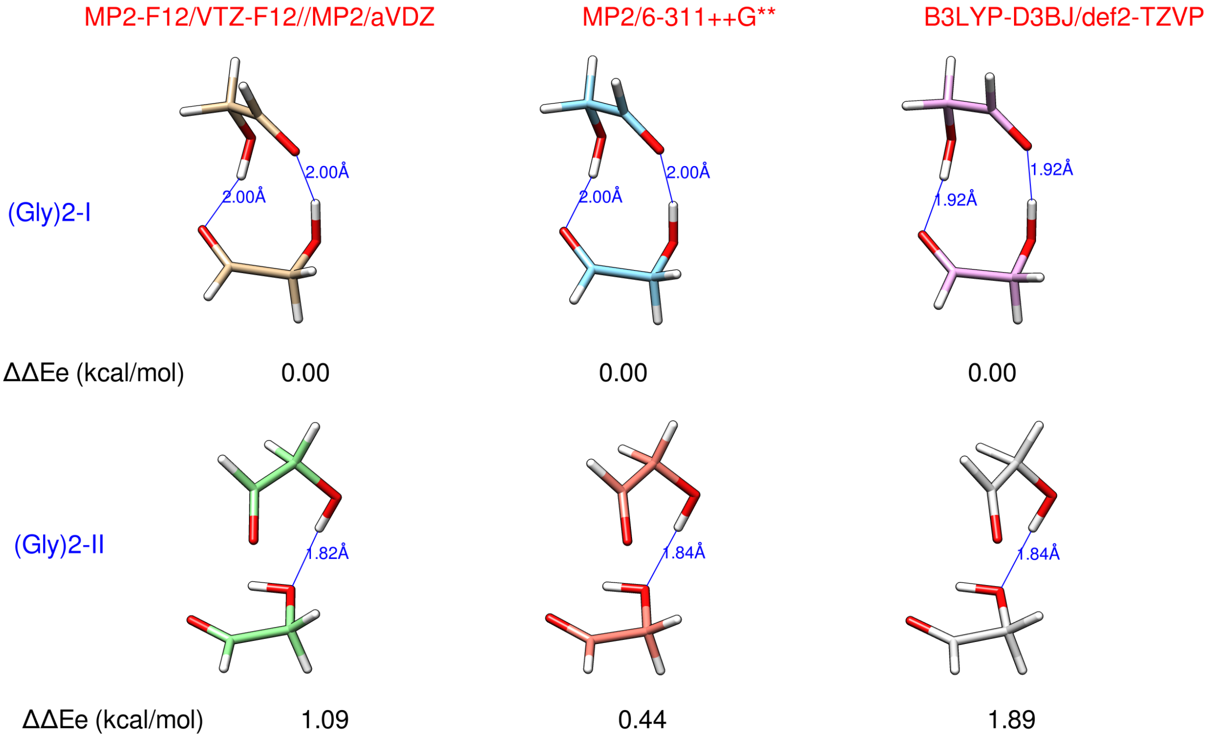


Figure S5: Comparison of the lowest energy isomers of $(Gly)_2$ computed using different QM methods. See Table S22 below.

Table S22: The rotational constants (A, B, C in MHz) and relative energies ($\Delta\Delta E_e$ in kcal $mol^{-1}$) of the low energy isomers of $(Gly)_2$ calculated using MP2-F12/VTZ-F12//MP2/aVDZ, MP2/6-311++G(d,p) and B3LYP-D3BJ/def2-TZVP

| Source | Rot. Constants (MHz) A | B | C | RMSE (MHz) vs. Expt. | Relative Energy $\Delta\Delta E_e$ (kcal $mol^{-1}$) |
|---|---|---|---|---|---|
| **Conformer $(Gly)_2$-I** | | | | | |
| Experiment(Zinn et al.[S17])[a] | 2792.7 | 1283.0 | 1198.2 | - | - |
| MP2/aVDZ[b] | 2756.5 | 1321.8 | 1259.6 | 46.9 | 0.00 |
| MP2/6-311++G(d,p) | 2811.9 | 1274.3 | 1201.5 | 12.3 | 0.00 |
| B3LYP-D3BJ/def2-TZVP | 2786.6 | 1277.4 | 1150.9 | 27.7 | 0.00 |
| **Conformer $(Gly)_2$-II** | | | | | |
| Experiment(Zinn et al.[S17])[a] | 2938.8 | 1170.3 | 964.3 | - | - |
| MP2/aVDZ[b] | 2840.9 | 1264.6 | 1016.0 | 84.0 | 1.09 |
| MP2/6-311++G(d,p) | 2867.0 | 1254.2 | 1022.4 | 72.0 | 0.44 |
| B3LYP-D3BJ/def2-TZVP | 2833.8 | 1224.4 | 999.6 | 71.2 | 1.89 |

[a] Rotational constants rounded off to $10^{th}$ decimal place to ease comparison with computed rotational constants
[b] $\Delta\Delta E_e$ calculated using MP2-F12/VTZ-F12//MP2/aVDZ energies

### 2.3.2 $(Gly)_2W_1$

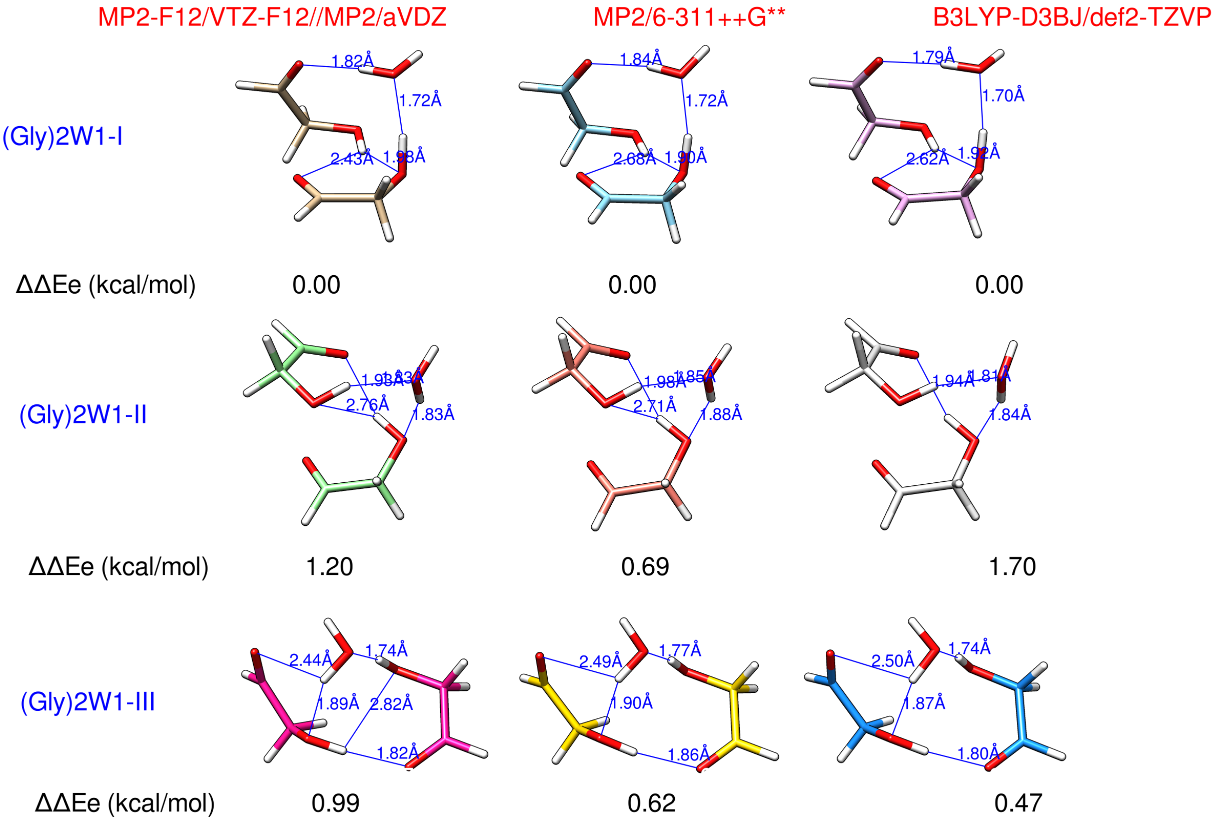


Figure S6: Comparison of the lowest energy isomers of $(Gly)_2W_1$ computed using different QM methods. See Table S23 below.

Table S23: The rotational constants (A, B, C in MHz) and relative energies ($\Delta\Delta E_e$ in kcal $mol^{-1}$) of the low energy isomers of $(Gly)_2W_1$ calculated using MP2-F12/VTZ-F12//MP2/aVDZ, MP2/6-311++G(d,p) and B3LYP-D3BJ/def2-TZVP

| Source | Rot. Constants (MHz) A | B | C | RMSE (MHz) vs. Expt. | Relative Energy $\Delta\Delta E_e$ (kcal $mol^{-1}$) |
|---|---|---|---|---|---|
| **Conformer $(Gly)_2W_1$-I** | | | | | |
| Experiment(current work)[a] | 1707.5 | 999.0 | 862.1 | - | - |
| MP2/aVDZ[b] | 1711.6 | 1025.0 | 901.2 | 27.2 | 0.00 |
| MP2/6-311++G(d,p) | 1658.7 | 1019.5 | 899.4 | 37.4 | 0.00 |
| B3LYP-D3BJ/def2-TZVP | 1660.4 | 1037.8 | 884.5 | 37.5 | 0.00 |
| **Conformer $(Gly)_2W_1$-II** | | | | | |
| MP2/aVDZ[b] | 1498.1 | 1285.2 | 968.1 | - | 1.20 |
| MP2/6-311++G(d,p) | 1516.4 | 1238.2 | 935.5 | - | 0.69 |
| B3LYP-D3BJ/def2-TZVP | 1532.6 | 1226.6 | 942.1 | - | 1.70 |
| **Conformer $(Gly)_2W_1$-III** | | | | | |
| MP2/aVDZ[b] | 2122.0 | 844.5 | 763.3 | - | 0.99 |
| MP2/6-311++G(d,p) | 2170.3 | 811.4 | 729.6 | - | 0.62 |
| B3LYP-D3BJ/def2-TZVP | 2158.0 | 830.7 | 740.7 | - | 0.47 |

[a] Rotational constants rounded off to $10^{th}$ decimal place to ease comparison with computed rotational constants
[b] $\Delta\Delta E_e$ calculated using MP2-F12/VTZ-F12//MP2/aVDZ energies

### 2.3.3 $(Gly)_2W_2$

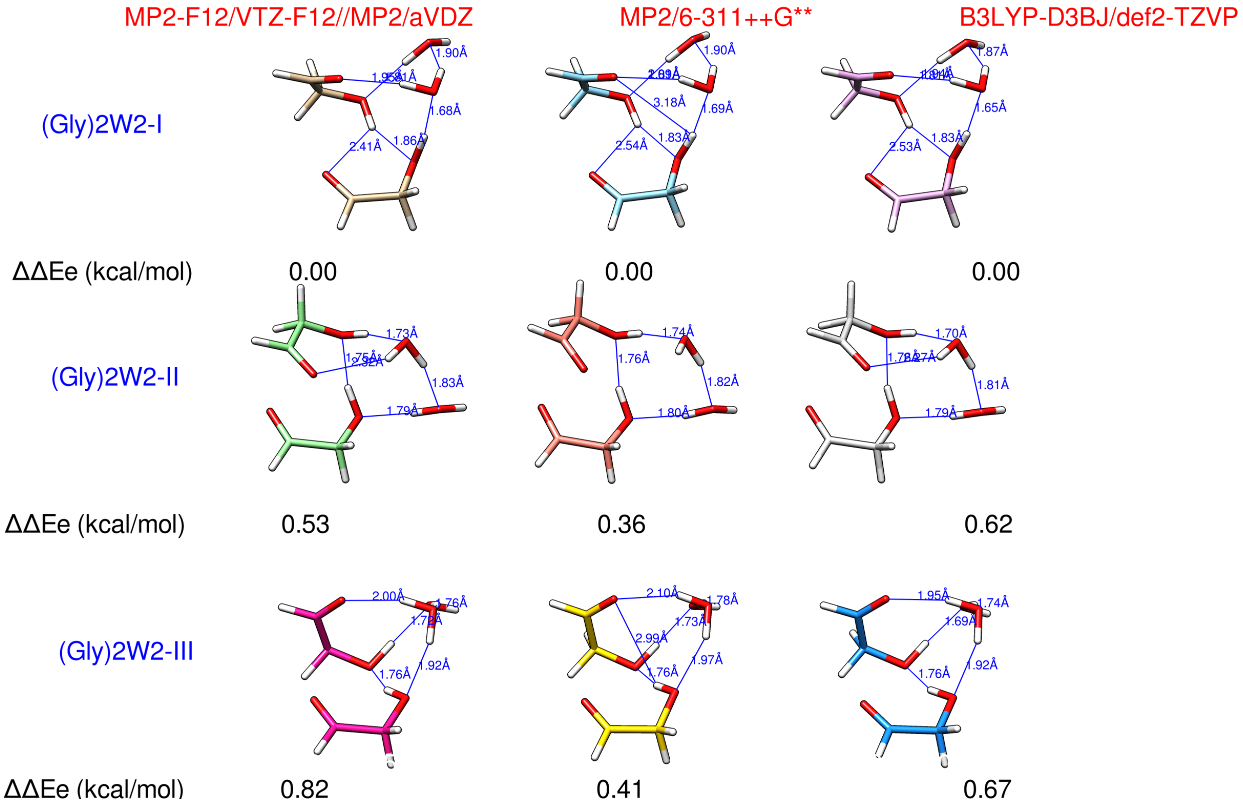


Figure S7: Comparison of the lowest energy isomers of $(Gly)_2W_2$ computed using different QM methods. See Table S24 below.

Table S24: The rotational constants (A, B, C in MHz) and relative energies ($\Delta\Delta E_e$ in kcal mol$^{-1}$) of the low energy isomers of $(Gly)_2W_2$ calculated using MP2-F12/VTZ-F12//MP2/aVDZ, MP2/6-311++G(d,p) and B3LYP-D3BJ/def2-TZVP

| Source | Rot. Constants (MHz) A | B | C | RMSE (MHz) vs. Expt. | Relative Energy $\Delta\Delta E_e$ (kcal mol$^{-1}$) |
|---|---|---|---|---|---|
| **Conformer $(Gly)_2W_2$-I** | | | | | |
| Experiment(current work)[a] | 1160.0 | 860.7 | 659.8 | - | - |
| MP2/aVDZ[b] | 1183.4 | 878.7 | 675.4 | 19.3 | 0.00 |
| MP2/6-311++G(d,p) | 1204.6 | 863.7 | 665.5 | 26.0 | 0.00 |
| B3LYP-D3BJ/def2-TZVP | 1189.5 | 862.2 | 668.5 | 17.8 | 0.00 |
| **Conformer $(Gly)_2W_2$-II** | | | | | |
| MP2/aVDZ[b] | 1132.1 | 953.4 | 657.3 | - | 0.53 |
| MP2/6-311++G(d,p) | 1111.6 | 921.1 | 625.0 | - | 0.36 |
| B3LYP-D3BJ/def2-TZVP | 1111.7 | 950.6 | 645.3 | - | 0.62 |
| **Conformer $(Gly)_2W_2$-III** | | | | | |
| MP2/aVDZ[b] | 1240.1 | 819.8 | 655.9 | - | 0.82 |
| MP2/6-311++G(d,p) | 1262.2 | 797.0 | 640.2 | - | 0.41 |
| B3LYP-D3BJ/def2-TZVP | 1247.3 | 804.1 | 648.1 | - | 0.67 |

[a] Rotational constants rounded off to 10$^{th}$ decimal place to ease comparison with computed rotational constants
[b] $\Delta\Delta E_e$ calculated using MP2-F12/VTZ-F12//MP2/aVDZ energies

### 2.3.4 $(Gly)_2W_3$

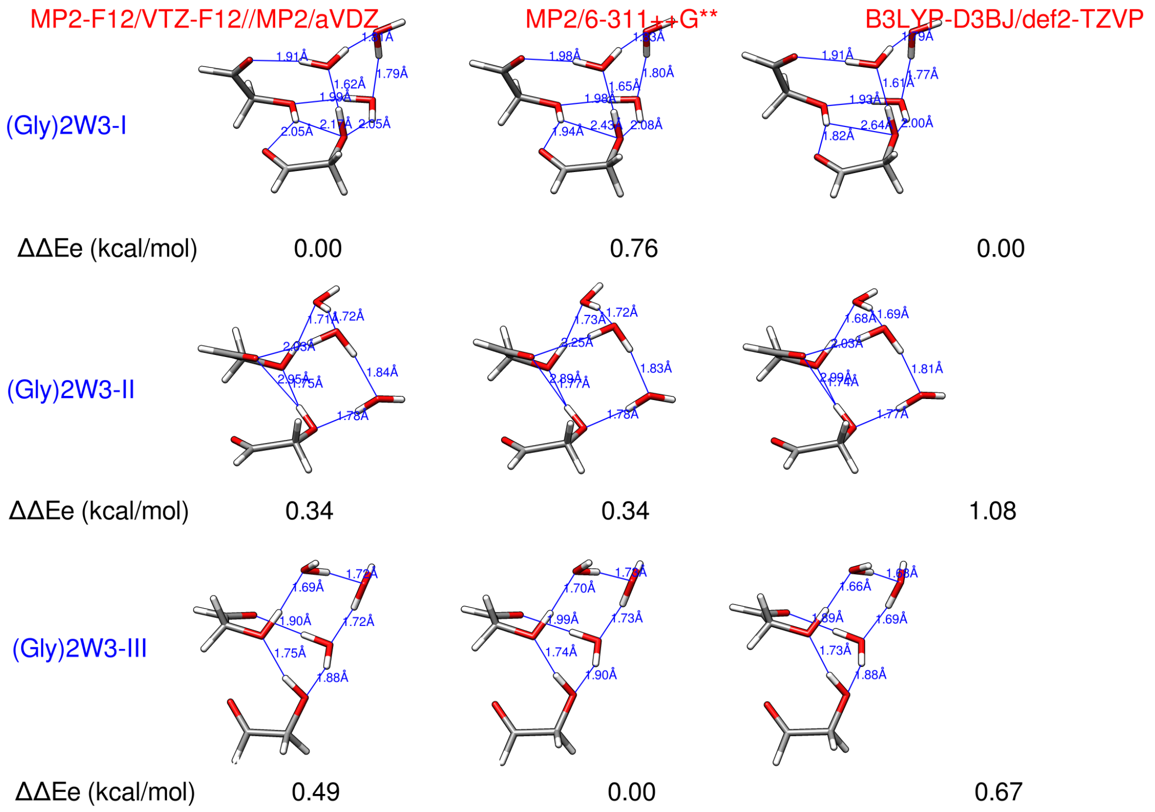


Figure S8: Comparison of the lowest energy isomers of $(Gly)_2W_3$ computed using different QM methods. See Table S25 below.

Table S25: The rotational constants (A, B, C in MHz) and relative energies ($\Delta\Delta E_e$ in kcal mol$^{-1}$) of the low energy isomers of $(Gly)_2W_3$ calculated using MP2-F12/VTZ-F12//MP2/aVDZ, MP2/6-311++G(d,p) and B3LYP-D3BJ/def2-TZVP

| Source | Rot. Constants (MHz) A | B | C | RMSE (MHz) Vs. Expt. | Relative Energy (kcal mol$^{-1}$) $\Delta\Delta E_e$ |
|---|---|---|---|---|---|
| **Conformer $(Gly)_2W_3$-I** | | | | | |
| Experiment(this work) | 790.0 | 673.9 | 507.1 | | |
| MP2/aVDZ6 | 817.3 | 687.5 | 525.2 | 20.5 | 0.00 |
| MP2/6-311++G(d,p) | 767.1 | 712.0 | 511.3 | 25.8 | 0.76 |
| B3LYP-D3BJ/def2-TZVP | 744.8 | 732.5 | 513.2 | 42.9 | 0.00 |
| **Conformer $(Gly)_2W_3$-II** | | | | | |
| MP2/aVDZ$^b$ | 889.1 | 736.3 | 519.7 | - | 0.34 |
| MP2/6-311++G(d,p) | 898.4 | 714.0 | 507.2 | - | 0.34 |
| B3LYP-D3BJ/def2-TZVP | 898.4 | 714.0 | 507.2 | - | 1.08 |
| **Conformer $(Gly)_2W_3$-III** | | | | | |
| MP2/aVDZ$^b$ | 984.7 | 581.2 | 479.5 | - | 0.49 |
| MP2/6-311++G(d,p) | 992.9 | 564.9 | 469.2 | - | 0.00 |
| B3LYP-D3BJ/def2-TZVP | 989.6 | 568.2 | 476.0 | - | 0.67 |

$^a$ Rotational constants rounded off to $10^{th}$ decimal place to ease comparison with computed rotational constants
$^b$ $\Delta\Delta E_e$ calculated using MP2-F12/VTZ-F12//MP2/aVDZ energies

### 2.3.5 Takeaways

The main takeways from this exercise are:

- Of the three most commonly used QM methods for getting structures to be compared with fits from broadband rotational spectroscopy, none consistently outperform the others. The comparisons and reported RMSE values in Tables S22 to S25 demonstrate that point. For example, from the three levels of theory compared here, the best performers were
  - $(Gly)_2$ - MP2/6-311++G(d,p)
  - $(Gly)_2W_1$ - MP2/aVDZ
  - $(Gly)_2W_2$ - B3LYP-D3BJ/def2-TZVP
  - $(Gly)_2W_3$ - MP2/aVDZ
- DFT structures and energies were very sensitive to
  - starting structure
  - integration grid
  - inclusion of dispersion correction (DFT-D2, DFT-D3) and damping (DFT-D3BJ),
  - the implementation and versions of different packages (Gaussian, Orca, Psi4, Molpro, ... )

- As a result of this sensitivity, it was difficult to reproduce DFT structures and energies reported in past literature. For instance, reproducing the B3LYP-D3/aVTZ and B3LYP-D3BJ/def2-TZVP reported by Zinn *et al.*[S17] for Gly)$_2$'s two experimentally fitted conformers was not possible unless we used (a) Gaussian09 Rev D.01 and (b) started with the same precise structure the authors started with. On the other hand, we were able to reproduce their MP2/def2-TZVP and MP2/aVTZ structures using Gaussian16 Rev B.01 as well as Orca 4.1.2 and 4.2.1.

## 2.4 Many-Body Expansion of Cluster Interaction Energies

### 2.4.1 Definition of Many-Body Expansion

The total binding energy of a molecular cluster ($E_{bind}$) is often calculated in supermolecular approaches as the difference in energy between the cluster ($E_{cluster}$) and the $N$ monomers constituting the cluster($E^i_m$) in their isolated gas phase minimum geometries.[S18]

$$E_{bind} = E_{cluster} - \sum_{i}^{N} E^i_m \tag{5}$$

While binding energies calculated this way are meaningful, it is much more interesting to examine the energy associated with distortion of the monomers as they form a cluster, and the interactions between the monomers or groups thereof. When formulated that way, the binding energy is a sum of the monomer distortion energy ($E_{dist}$) and interaction energy ($E_{int}$) of a cluster.

$$E_{bind} = E_{dist} + E_{int} \tag{6}$$

The monomer distortion or relaxation energy ($E_{dist}$), which is also referred to as one-body energy ($E_{1B}$), is difference in energy between monomers in their isolated gas phase geometry ($E^i_m$) and cluster form ($E^i_c$)

$$E_{dist} = E_{1B} = \sum_{i}^{N} \left[ E^i_c - E^i_m \right] \tag{7}$$

The interaction energy ($E_{int}$) can be expanded into its many-body components. The total interaction energy of a molecular cluster composed of N monomers is the sum of its two-body (2B), three-body (3B), four-body (4B), ... , N-body (NB) components.

$$E_{int} = E_{2B} + E_{3B} + E_{4B} + ... + E_{NB} \tag{8}$$

$E_{2B}$ is the pair-wise interaction between all pairs of monomers in their cluster geometry

$$E_{2B} = \sum_{i>j}^{N} \left[ E^{ij}_c - (E^i_c + E^j_c) \right] \tag{9}$$

$E_{3B}$ is the interaction between all sets of three monomers in their cluster geometry, excluding the 2B contribution

$$E_{3B} = \sum_{i>j>k}^{N} \left[ E^{ijk}_c - (E^i_c + E^j_c + E^k_c) \right] - E_{2B} \tag{10}$$

$E_{4B}$ is the interaction between all sets of four monomers in their cluster geometry, excluding the smaller many-body contributions

$$E_{4B} = \sum_{i>j>k>l}^{N} \left[ E_c^{ijkl} - (E_c^i + E_c^j + E_c^k + E_c^l) \right] - E_{3B} - E_{2B} \tag{11}$$

and the general n-body contribution is

$$E_{nB} = \sum_{i>j...n}^{N} \left[ E_c^{ij...n} - (E_c^i + E_c^j + ... + E_c^n) \right] - \sum_{a=2}^{n-1} E_{aB} \tag{12}$$

The monomer distortion or relaxation energy ($E_{dist}$, $E_{1B}$) is a generally positive quantity because monomers are adopting configurations with higher energies than in their isolated gas phase geometry in order to optimize their interactions with the other monomers constituting the cluster. Two-body interactions ($E_{2B}$) are the largest contributor to the stabilization of clusters, accounting for more than 80% of the total interaction energy in water clusters. Three-body interactions ($E_{3B}$) are also important especially in clusters composed of polar molecules where long-range forces are strong. $E_{3B}$ generally accounts for 10-15% of the interaction energy in water clusters. Four- and other higher-body interactions may be non-negligible depending on the system and the desired level of accuracy.[S19]

For our purposes, many-body decomposition of the interaction energy provides insights into the strength of interactions between any pairs or triplets of monomers. Using a many-body decomposition code,[S20] we calculated all individual many-body interaction energy components between all sets of monomers to isolate different interactions.

Just to emphasize the subtle difference between the total interaction energy and binding energy, the two terms are defined below. Binding energy is the difference between the energy of the cluster and the energy of the isolated monomers in their minimum configuration. Interaction energy is the difference between the energy of the cluster and the energy of the isolated monomers in the cluster geometry. The difference between binding and interaction energy is the monomer distortion or relaxation energy ($E_{dist}$, $E_{1B}$).

### 2.4.2 $(Gly)_2$

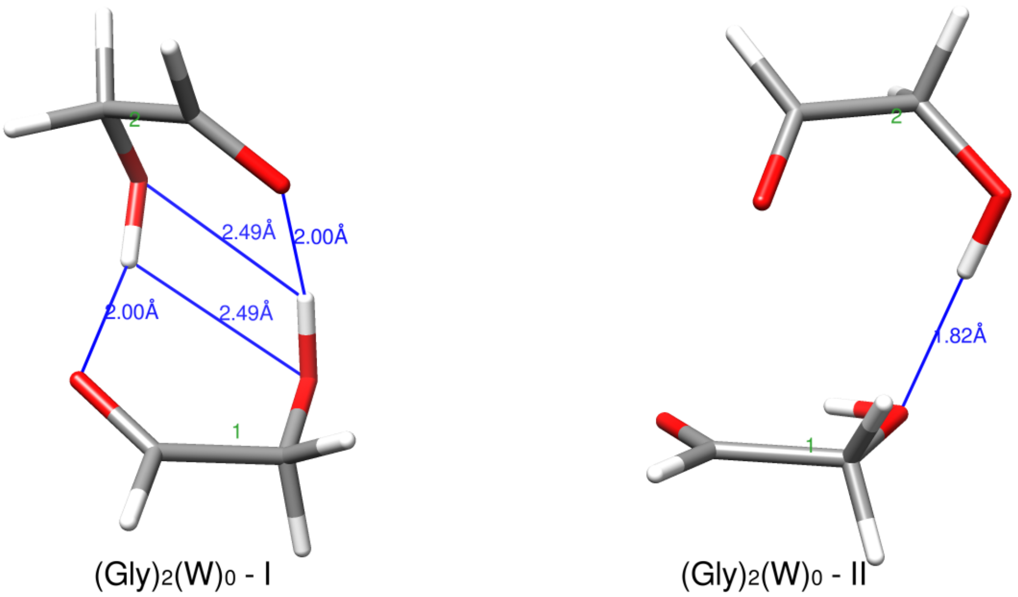


Figure S9: The two MP2-F12/VTZ-F12//MP2/aVDZ lowest energy isomers of $(Gly)_2$. The numbers in green correspond to the monomer/fragment labels in the table below.

Table S26: Many-body decomposition of the MP2-F12/VTZ-F12//MP2/aVDZ binding energy of two $(Gly)_2(W)_0$ isomers in kJ mol$^{-1}$

| | $Gly_2$-I | $Gly_2$-II |
|---|---|---|
| Monomers: | | |
| 1 = | Gly | Gly |
| 2 = | Gly | Gly |
| | | |
| Individual 1-body contributions: | | |
| Fragment 1(1) : | 14.6 | 1.8 |
| Fragment 2(2) : | 14.6 | 12.6 |
| | | |
| Individual 2-body contributions: | | |
| Fragment 1(1-2) : | -62.8 | -43.6 |
| | | |
| Many-body energies: | | |
| 1-body = | 29.1 | 14.4 |
| 2-body = | -62.8 | -43.6 |
| | | |
| Interaction Energy = | -62.8 | -43.6 |
| **Binding Energy =** | **-33.7** | **-29.1** |

### 2.4.3 $(Gly)_2W_1$

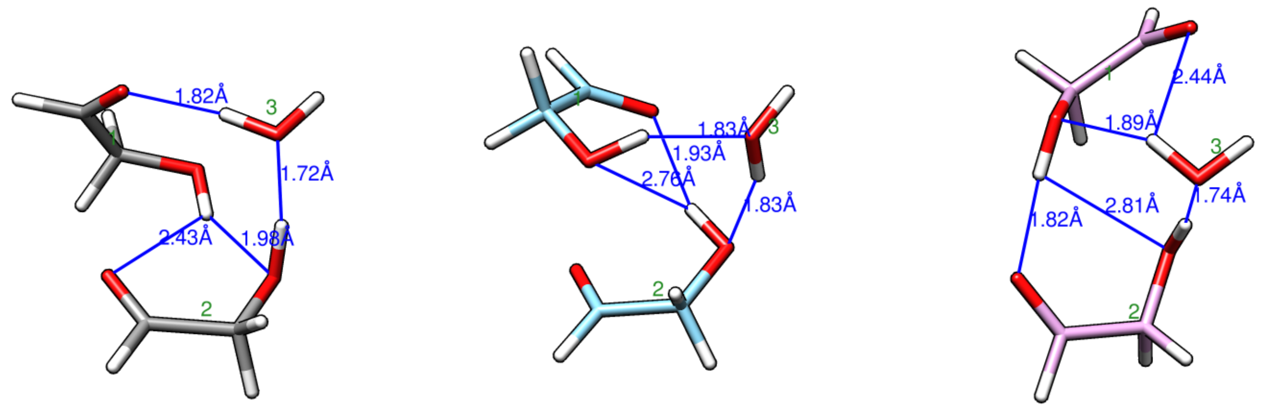


$(Gly)_2(W)_1$ - I $(Gly)_2(W)_1$-II $(Gly)_2(W)_1$-III

Figure S10: The three MP2-F12/VTZ-F12//MP2/aVDZ lowest energy isomers of $(Gly)_2(W)_1$. The numbers in green correspond to the monomer/fragment labels in the table below.

Table S27: Many-body decomposition of the MP2-F12/VTZ-F12//MP2/aVDZ binding energy of three $(Gly)_2(W)_1$ isomers in kJ $mol^{-1}$

| | $Gly_2W_1$-I | $Gly_2W_1$-II | $Gly_2W_1$-III |
|---|---|---|---|
| Monomers: | | | |
| 1 = | Gly | Gly | Gly |
| 2 = | Gly | Gly | Gly |
| 3 = | W | W | W |
| | | | |
| Individual 1-body contributions: | | | |
| Fragment 1(1) : | 17.2 | 12.5 | 25.8 |
| Fragment 2(2) : | 20.4 | 4.8 | 18.5 |
| Fragment 3(3) : | 1.3 | 1.3 | 1.2 |
| | | | |
| Individual 2-body contributions: | | | |
| Fragment 1(1-2) : | -40.6 | -29.7 | -42.0 |
| Fragment 2(1-3) : | -24.9 | -27.9 | -26.2 |
| Fragment 3(2-3) : | -29.9 | -21.3 | -28.5 |
| | | | |
| Individual 3-body contributions: | | | |
| Fragment 1(1-2-3) : | -16.5 | -7.8 | -18.5 |
| | | | |
| Many-body energies: | | | |
| 1-body = | 38.9 | 18.5 | 45.5 |
| 2-body = | -95.5 | -78.8 | -96.8 |
| 3-body = | -16.5 | -7.8 | -17.7 |
| | | | |
| Interaction Energy = | -112.0 | -86.7 | -114.5 |
| **Binding Energy** = | **-73.1** | **-68.1** | **-69.0** |

### 2.4.4 $(Gly)_2W_2$

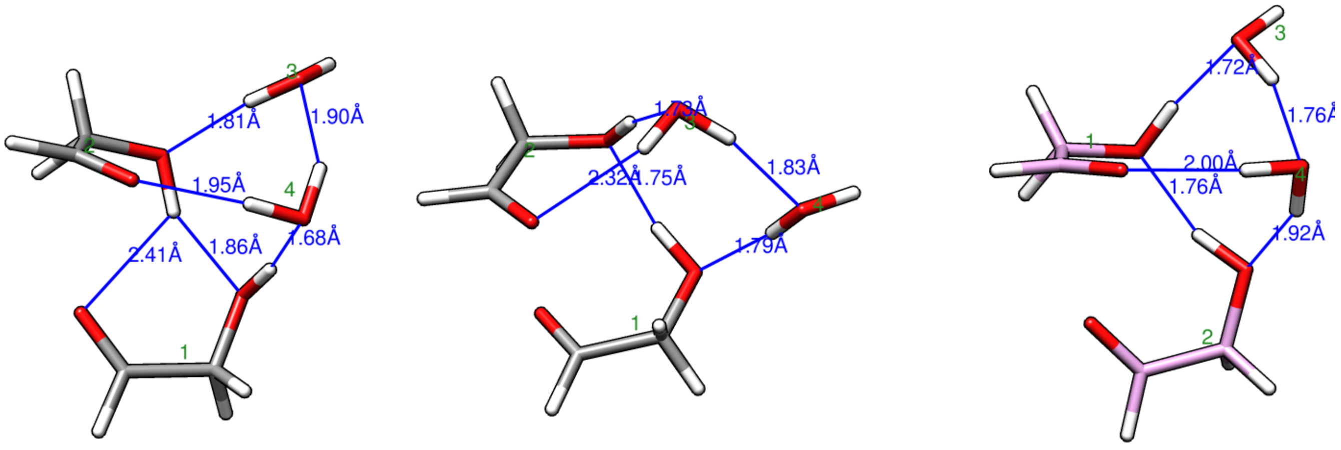


$(Gly)_2(W)_2$ - I $(Gly)_2(W)_2$ - II $(Gly)_2(W)_2$ - III

Figure S11: The three MP2-F12/VTZ-F12//MP2/aVDZ lowest energy isomers of $(Gly)_2(W)_2$. The numbers in green correspond to the monomer/fragment labels in the table below.

Table S28: Many-body decomposition of the MP2-F12/VTZ-F12//MP2/aVDZ binding energy of two $(Gly)_2(W)_2$ isomers in kJ $mol^{-1}$

| | $Gly_2W_2$-I | $Gly_2W_2$-II |
|---|---|---|
| Monomers: | | |
| 1 = | Gly | Gly |
| 2 = | Gly | Gly |
| 3 = | W | W |
| 4 = | W | W |
| | | |
| Individual 1-body contributions: | | |
| Fragment 1(1) : | 25.6 | 10.1 |
| Fragment 2(2) : | 15.4 | 12.3 |
| Fragment 3(3) : | 1.2 | 1.3 |
| Fragment 4(4) : | 1.6 | 1.6 |
| | | |
| Individual 2-body contributions: | | |
| Fragment 1(1-2) : | -45.1 | -39.3 |
| Fragment 2(1-3) : | -9.1 | -3.4 |
| Fragment 3(1-4) : | -25.4 | -22.3 |
| Fragment 4(2-3) : | -16.9 | -34.4 |
| Fragment 5(2-4) : | -22.5 | -6.2 |
| Fragment 6(3-4) : | -15.0 | -18.8 |
| | | |
| Individual 3-body contributions: | | |
| Fragment 1(1-2-3) : | -5.0 | -3.3 |
| Fragment 2(1-2-4) : | -16.7 | -4.0 |
| Fragment 3(1-3-4) : | -6.9 | -4.8 |
| Fragment 4(2-3-4) : | 0.0 | -5.3 |
| | | |
| Individual 4-body contributions: | | |
| Fragment 1(1-2-3-4) : | -1.3 | -1.3 |
| | | |
| Many-body energies: | | |
| 1-body = | 43.8 | 25.3 |
| 2-body = | -133.9 | -124.4 |
| 3-body = | -28.6 | -17.4 |
| 4-body = | -1.3 | -1.3 |
| | | |
| Interaction Energy = | -163.9 | -143.1 |
| **Binding Energy** = | **-120.0** | **-117.8** |

### 2.4.5 $(Gly)_2W_3$

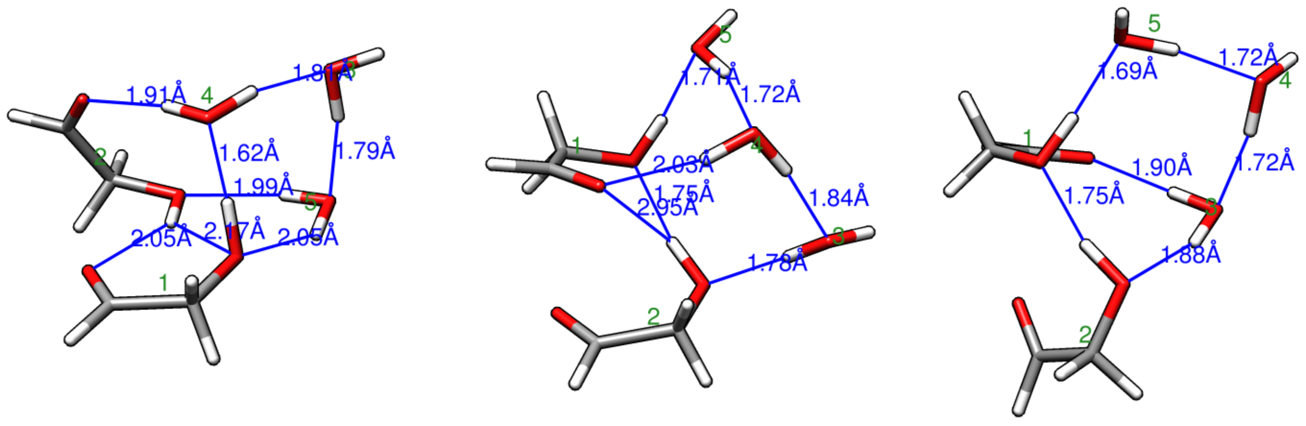


(Gly)2(W)3 - I (Gly)2(W)3 - II (Gly)2(W)3 - III

Figure S12: The three MP2-F12/VTZ-F12//MP2/aVDZ lowest energy isomers of $(Gly)_2(W)_3$. The numbers in green correspond to the monomer/fragment labels in the table below.

Table S29: Many-body decomposition of the MP2-F12/VTZ-F12//MP2/aVDZ binding energy of three $(Gly)_2(W)_3$ isomers in kJ $mol^{-1}$

| | $Gly_2W_3$-I | $Gly_2W_3$-II | $Gly_2W_3$-III |
|---|---|---|---|
| Monomers: | | | |
| 1 = | Gly | Gly | Gly |
| 2 = | Gly | Gly | Gly |
| 3 = | W | W | W |
| 4 = | W | W | W |
| 5 = | W | W | W |
| | | | |
| Individual 1-body contributions: | | | |
| Fragment 1(1) : | 22.0 | 17.9 | 17.2 |
| Fragment 2(2) : | 14.6 | 10.2 | 8.5 |
| Fragment 3(3) : | 1.8 | 1.6 | 1.4 |
| Fragment 4(4) : | 1.9 | 1.3 | 2.6 |
| Fragment 5(5) : | 1.1 | 2.9 | 2.3 |
| Individual 2-body contributions: | | | |
| Fragment 1(1-2) : | -38.2 | -41.3 | -29.8 |
| Fragment 2(1-3) : | -6.9 | -3.0 | -20.9 |
| Fragment 3(1-4) : | -27.2 | -23.0 | -8.9 |
| Fragment 4(1-5) : | -17.2 | -30.6 | -27.7 |
| Fragment 5(2-3) : | 0.6 | -22.7 | -16.3 |
| Fragment 6(2-4) : | -24.2 | -1.0 | -2.7 |
| Fragment 7(2-5) : | -9.3 | -2.1 | -2.5 |
| Fragment 8(3-4) : | -19.1 | -16.5 | -17.7 |
| Fragment 9(3-5) : | -19.7 | -4.3 | -6.5 |
| Fragment 10(4-5) : | -5.3 | -17.2 | -18.6 |
| Individual 3-body contributions: | | | |
| Fragment 1(1-2-3) : | 0.3 | -3.4 | 1.5 |
| Fragment 2(1-2-4) : | -14.7 | 0.0 | -0.2 |
| Fragment 3(1-2-5) : | 0.2 | -4.0 | -4.2 |
| Fragment 4(1-3-4) : | -7.5 | 2.0 | -6.3 |
| Fragment 5(1-3-5) : | -4.5 | -1.3 | -3.8 |
| Fragment 6(1-4-5) : | -4.9 | -14.3 | -9.4 |
| Fragment 7(2-3-4) : | 3.0 | -3.8 | -4.7 |
| Fragment 8(2-3-5) : | -2.4 | -0.9 | -0.6 |
| Fragment 9(2-4-5) : | 1.3 | 0.2 | -0.9 |
| Fragment 10(3-4-5) : | -6.0 | -4.9 | -7.4 |
| Individual 4-body contributions: | | | |
| Fragment 1(1-2-3-4) : | 0.1 | 0.0 | 0.0 |
| Fragment 2(1-2-3-5) : | 0.0 | -0.8 | -0.3 |
| Fragment 3(1-2-4-5) : | 0.3 | -0.5 | -0.5 |
| Fragment 4(1-3-4-5) : | -1.8 | -0.5 | -2.3 |
| Fragment 5(2-3-4-5) : | 0.0 | -0.3 | -0.7 |
| Individual 5-body contributions: | | | |
| Fragment 1(1-2-3-4-5) : | 0.1 | -0.2 | -0.1 |
| | | | |
| Many-body energies: | | | |
| 1-body = | 41.4 | 34.1 | 31.9 |
| 2-body = | -166.4 | -161.7 | -151.7 |
| 3-body = | -35.4 | -30.4 | -36.1 |
| 4-body = | -1.5 | -2.1 | -3.7 |
| 5-body = | 0.1 | -0.2 | -0.1 |
| | | | |
| Interaction Energy = | -203.1 | -194.3 | -191.6 |
| **Binding Energy** = | **-161.7** | **-160.1** | **-159.6** |

### 2.4.6 Comparison of $(Gly)_2$ and $(Gly)_1W_1$

To gain better perspective of the many-body effects in $(Gly)_2$, a MB decomposition of the two lowest energy isomers of $(Gly)_1W_1$ are given below in Table S30.

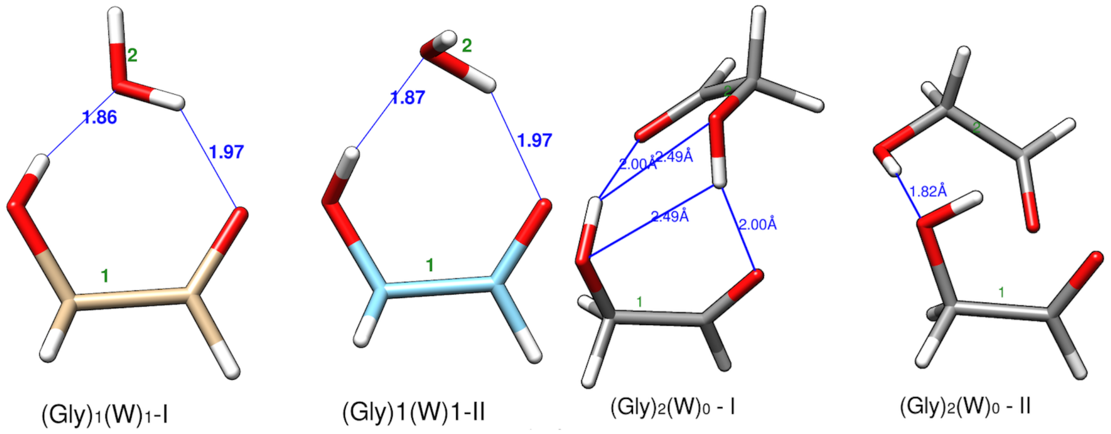


Figure S13: Comparison of $(Gly)_2$ and $(Gly)_1W_1$'s MP2-F12/VTZ-F12//MP2/aVDZ interaction energies. The numbers in green correspond to the monomer/fragment labels in the table below.

Table S30: Many-body decomposition of the MP2-F12/VTZ-F12//MP2/aVDZ binding energy of two $(Gly)_2$ and $(Gly)_1(W)_1$ isomers in kJ mol$^{-1}$

| | $Gly_1W_1$ | | $Gly_2$ | |
|---|---|---|---|---|
| | I | II | I | II |
| Monomers: | | | | |
| 1 = | Gly | Gly | Gly | Gly |
| 2 = | W | W | Gly | Gly |
| Individual 1-body contributions: | | | | |
| Fragment 1(1) : | 11.0 | 11.0 | 14.6 | 1.8 |
| Fragment 2(2) : | 0.6 | 0.6 | 14.6 | 12.6 |
| Individual 2-body contributions: | | | | |
| Fragment 1(1-2) : | -41.0 | -38.5 | -62.8 | -43.6 |
| Many-body energies: | | | | |
| 1-body = | 11.6 | 11.6 | 29.1 | 14.4 |
| 2-body = | -41.0 | -38.5 | -62.8 | -43.6 |
| Interaction Energy = | -41.0 | -38.5 | -62.8 | -43.6 |
| **Binding Energy =** | **-29.4** | **-26.9** | **-33.7** | **-29.1** |

### 2.4.7 Takeaways from MBE Analysis

The many-body decomposition of the binding energy for a few low energy isomers is reported below. Here are some interesting points to note about these results.

- **Competition between 1-body and many-body forces:** In some cases, the monomers distort (1-body effect) significantly to adapt configurations that maximize the strength of the hydrogen bonds with their neighbors (many-body effect). This is especially true of Gly monomers which distort to form strong hydrogen bonds.
- **The importance of 3-body interactions:** They generally account for about 20% of the interaction energy and vary substantially between isomers. Therefore, they are essential to modeling these kinds of system.
- **The importance of 4- and 5-body interactions:** 4-body interactions are small, but they are essential if one wants to achieve chemical accuracy (errors less than 1 kcal mol$^{-1}$. 5-body interactions are small enough to be negligible.
- **The strength of Gly-Gly, Gly-W and W-W interactions:** Gly-Gly interactions are consistently the strongest, while Gly-W are generally, but not consistently stronger than W-W interactions.
- **Bifurcated hydrogen bonds:** The two Gly monomers form either one conventional hydrogen bond or a bifurcated one comprised of one strong and one weak hydrogen bond. Analysis of the two-body interaction between the Gly monomers in all the clusters indicates that the bifurcated hydrogen bonds are cumulatively stronger than the conventional one.

Since Gly and W are very polar molecules, their clusters are expected to have a large many-body component. The data in Tables S26 - S29 does indeed indicate that the 3-body contribution to the interaction energy is large and that 4-body contribution is non-negligible.

## 2.5 Torsional Potentials of Glycoaldhyde

The glycoaldehyde monomer's global minimum structure is a *cis* conformer stabilized by an intramolecular hydrogen bond between the hydroxy and carbonyl groups.[S21] The geometry of the glycoaldehyde monomers in $(Gly)_2(W)_n = 0-3$ clusters distorts substantially from their isolated gas phase geometries to optimize the hydrogen bonding interactions with each other and solvating water molecules. These distortions are reflected in the large 1-body (deformation) energy of the glycoaldehydes in a large majority of the clusters studied here. The deformation energy for glycoaldehyde monomers in the different global minima ranges from 14.6 kJ $mol^{-1}$ for the $(Gly)_2$ to as high as 25.8 kJ $mol^{-1}$ for the $(Gly)_2(W)_3$. Most of these deformations involve the torsion of the hydroxyl group about the C-O and C-C axis to find the most direct hydrogen bonding orientation. Given the importance of this distortion, it would be interesting to see the energetics associated with these torsions. Figure S15 shows a relaxed potential energy plot of the O-H torsion about the C-O axis.

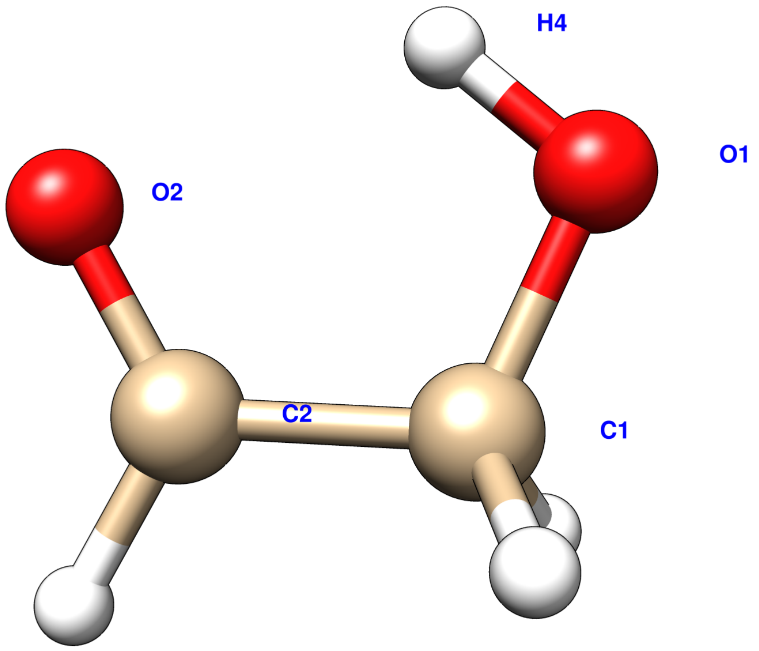


Figure S14: The MP2/aVDZ geometry of glycoaldehyde. The torsional potentials of the H4-O1 are about the O1-C1 axis, and O2-C2 about the C2-C1 axis are plotted below.

### 2.5.1 Rotation of -O-H Group about -C-O- Axis

The rotation potential of the H4-O1 about the O1-C1 axis is plotted below. The relevant dihedral angle we scan over is H4-O1-C1-C2 as labeled in Figure S14.

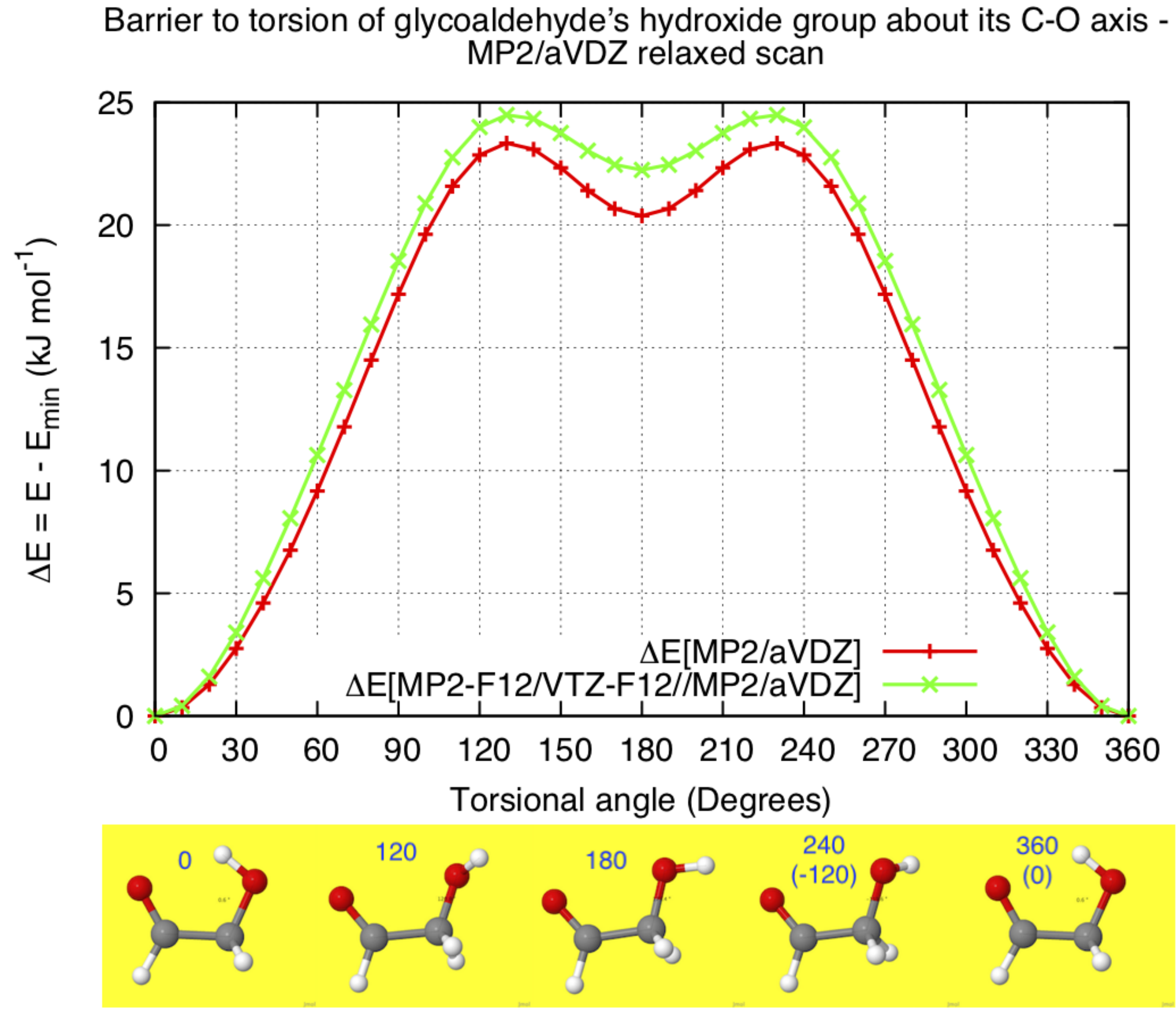


Figure S15: The MP2/aVDZ and MP2-F12/VTZ-F12//MP2/aVDZ relaxed potential energy scan of the O-H torsion about the adjacent C-O axis of glycoaldehyde (H4-O1-C1-C2 in Figure S14). The barrier to free rotation of the hydroxyl group is 20 - 25 kJ $mol^{-1}$.

### 2.5.2 Torsion about the C-C bond

Likewise, rotations about the central C-C axis has a barrier of 21 - 33 kJ mol$^{-1}$ at MP2/aVDZ level of theory, as shown in Figure S16. The torsional angle here is O2-C2-C1-O1 as labeled in Figure S14.

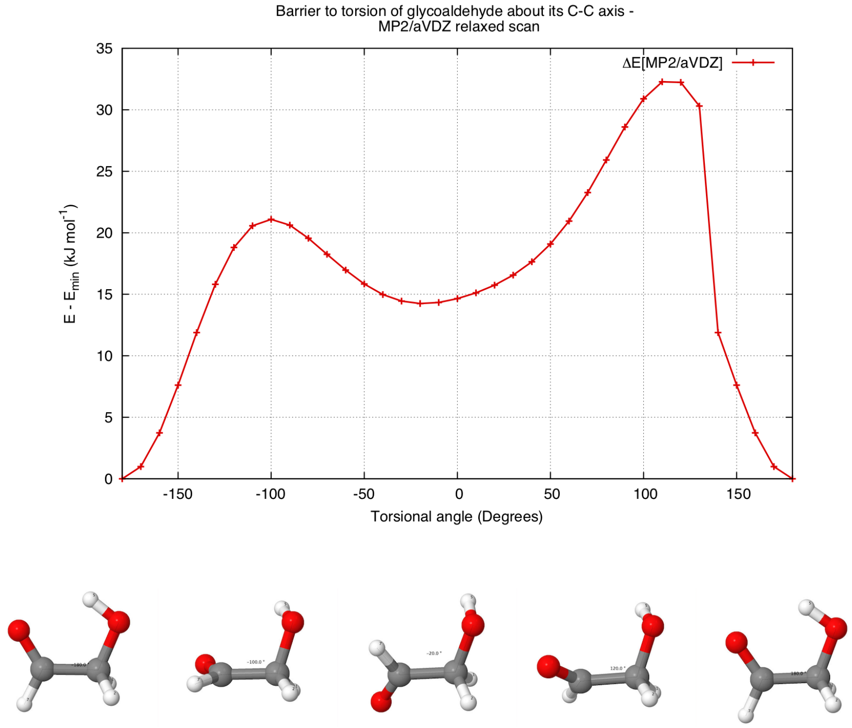


Figure S16: The MP2/aVDZ relaxed potential energy scan of glycoaldehyde about the central C-C bond. The relevant dihedral angle here is O2-C2-C1-O1 in Figure S14. The barrier to rotation about C-C axis is 21 - 33 kJ mol$^{-1}$.

## 2.6 Effects of Adding Water Molecules Incrementally

One of the interesting aspects of the hydration of glycoaldehyde dimer is that the structural features of the $(Gly)_2(W)_1$ global minimum are preserved in the $Gly)_2(W)_2$ and $(Gly)_2(W)_3$ global minima, as shown in Figure S17. The second water molecule of $Gly)_2(W)_2$ and the second and third water molecules of $Gly)_2(W)_3$ are removed for clarity.

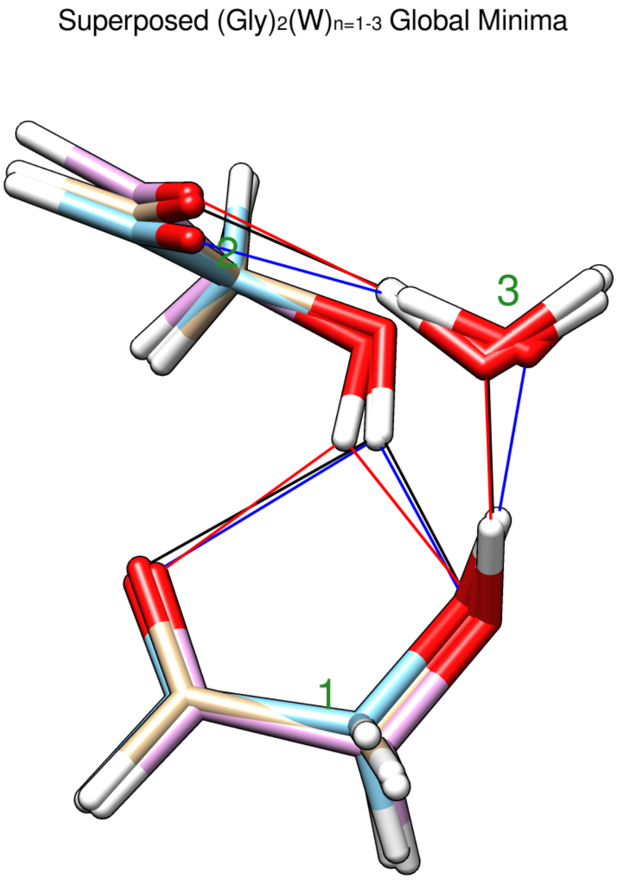


Figure S17: The global minima of $(Gly)_2(W)_{1-3}$ superimposed on The global minimum of $(Gly)_2(W)_1$ is preserved in $(Gly)_2(W)_2$ and $(Gly)_2(W)_3$. The second water molecule of $Gly)_2(W)_2$ and the second and third water molecules of $Gly)_2(W)_3$ are removed for clarity. The numbers in green correspond to the monomer/fragment labels in the Table S31.

Interestingly, the many body contributions of the interaction energy of these three global minima are also comparable, as shown in the table below.

Table S31: Many-body decomposition of the MP2-F12/VTZ-F12//MP2/aVDZ interaction energy of the global minima of $(Gly)_2(W)_{1-3}$ in kJ mol$^{-1}$. Only the components corresponding to the $(Gly)_2(W)_1$ unit in $(Gly)_2(W)_{1-3}$ shown in Figure S17 are reported here.

| | $Gly_2W_1$-I | $Gly_2W_2$-I | $Gly_2W_3$-I |
|---|---|---|---|
| Monomers: | | | |
| 1= | Gly | Gly | Gly |
| 2= | Gly | Gly | Gly |
| 3= | W | W | W |
| 4= | - | W | W |
| 5= | - | - | W |
| Individual 1-body contributions: | | | |
| Fragment 1(1) : | 17.2 | 25.6 | 22.0 |
| Fragment 2(2) : | 20.4 | 15.4 | 14.6 |
| Fragment 3(3) : | 1.3 | 1.6 | 1.9 |
| Individual 2-body contributions: | | | |
| Fragment 1(1-2) : | -40.6 | -45.1 | -38.2 |
| Fragment 2(1-3) : | -24.9 | -25.4 | -27.2 |
| Fragment 3(2-3) : | -29.9 | -22.5 | -24.2 |
| Individual 3-body contributions: | | | |
| Fragment 1(1-2-3) : | -16.5 | -16.7 | -14.7 |
| Total many-body contributions: | | | |
| 1-body = | 38.9 | 42.6 | 38.5 |
| 2-body = | -95.5 | -93.0 | -89.6 |
| 3-body = | -16.5 | -16.7 | -14.7 |
| **Total Binding Energy** = | **-73.1** | **-67.1** | **-65.8** |

## 2.7 Homochirality of Glycoaldehyde Monomers in $Gly_nW_m$ Clusters

As noted in the previous section, the addition of a water monomer to $Gly_2$ changes the structure of the dimer substantially and that change is preserved in the larger $Gly_2W_{n=2-3}$ clusters. Another notable point is that the glycolaldehyde monomers in all the observed clusters have the same chirality, suggesting that there is some molecular recognition. Chirality in this case refers to the rotation of the hydroxy group about the plane of symmetry, leading to non-superimposable structures as transient enantiomers.

To see if this handedness extends beyond the glycolaldehyde dimer and its hydrates, we calculated the global minimum structure of the $Gly_3$ and $Gly_3W_1$ using the same computational approach as the smaller clusters. The structures shown in Figure S18 demonstrate that $Gly_3$ and $Gly_3W_1$ exhibit the same preference for homochiral glycoaldehyde monomers as in the dimer and dimer hydrates.

The homochiral configuration allows the monomers to form cooperative, homodromic hydrogen bond cycles that confer them special stability. It is also worth noting that the third glycoaldehyde in $Gly_3$ takes the place of the water monomer in $Gly_2W_1$. The addition of a water monomer to $Gly_3$ leads to a $Gly_3W_1$ cluster with a homodromic tetramer hydrogen bond ring.

All in all, the binding motif observed in $Gly_2W_{n=1-3}$ is also preserved in $Gly_3$ and $Gly_3W$. The reorganization observed when the first water monomer is added to $Gly_2$ is preserved even in larger clusters. To see if this pattern extends to larger clusters would require work that is beyond the scope of this study.

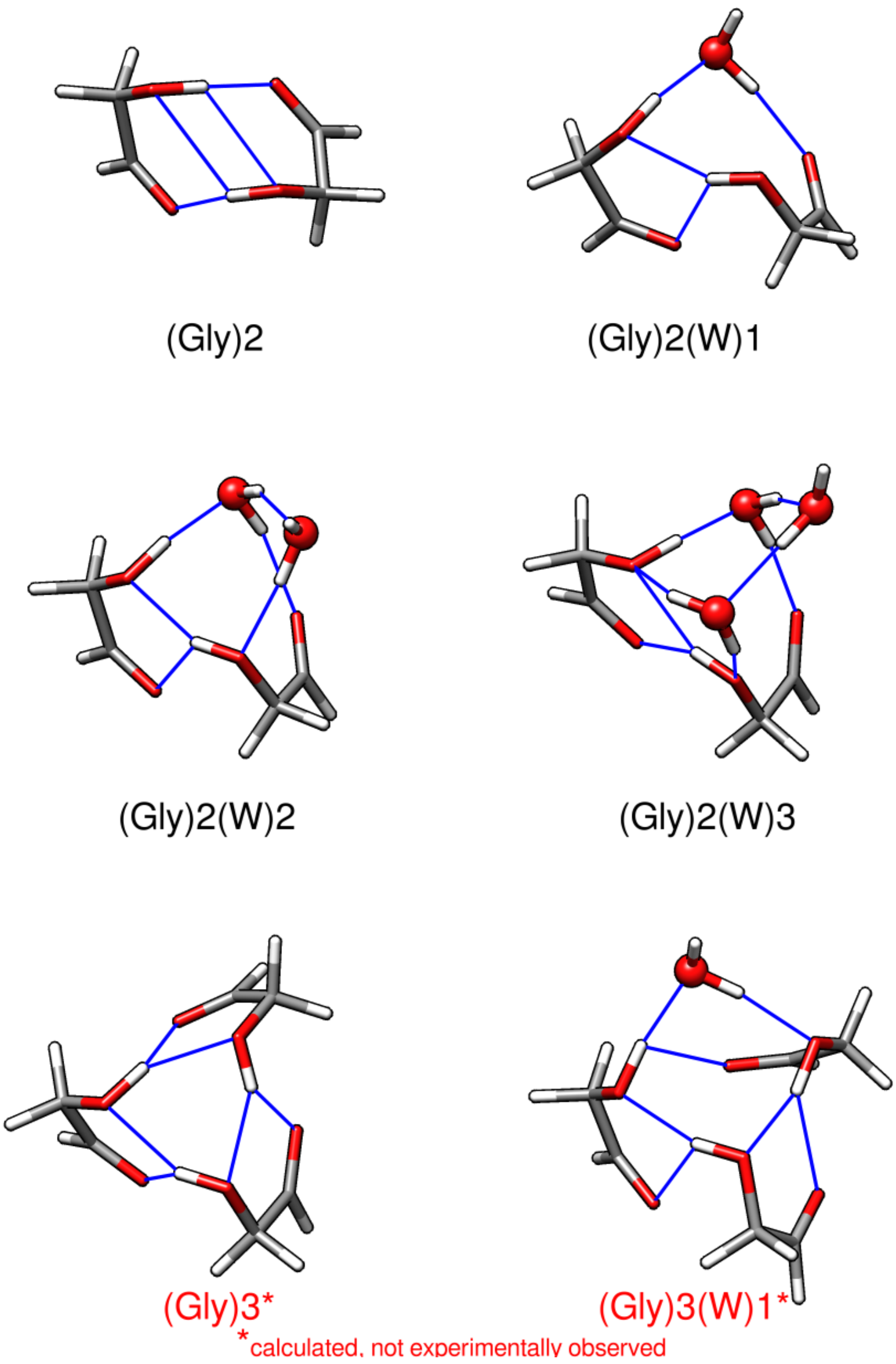


Figure S18: Experimentally observed structures of $(Gly)_2W_{m=0-3}$ and MP2/aVDZ calculated structures of $(Gly)_3W_{m=0-1}$.

## 2.8 Cartesian Coordinates

### 2.8.1 Glycoaldehyde and Water Monomers

The structures of the isomers discussed above are shown in Figure S19 and their MP2/aVDZ Cartesian coordinates are provided in Table S32-S42 below. In many cases, the presence of hydrogen bonds that either have bifurcation, long hydrogen bond distances or small hydrogen bonds angles may raise questions whether they can indeed be considered hydrogen bonds. They are considered hydrogen bonds based on the criteria set by Mills and Dean[S22] which is implemented in the UCSF Chimera[S23] used to generate the molecular graphics in this manuscript.

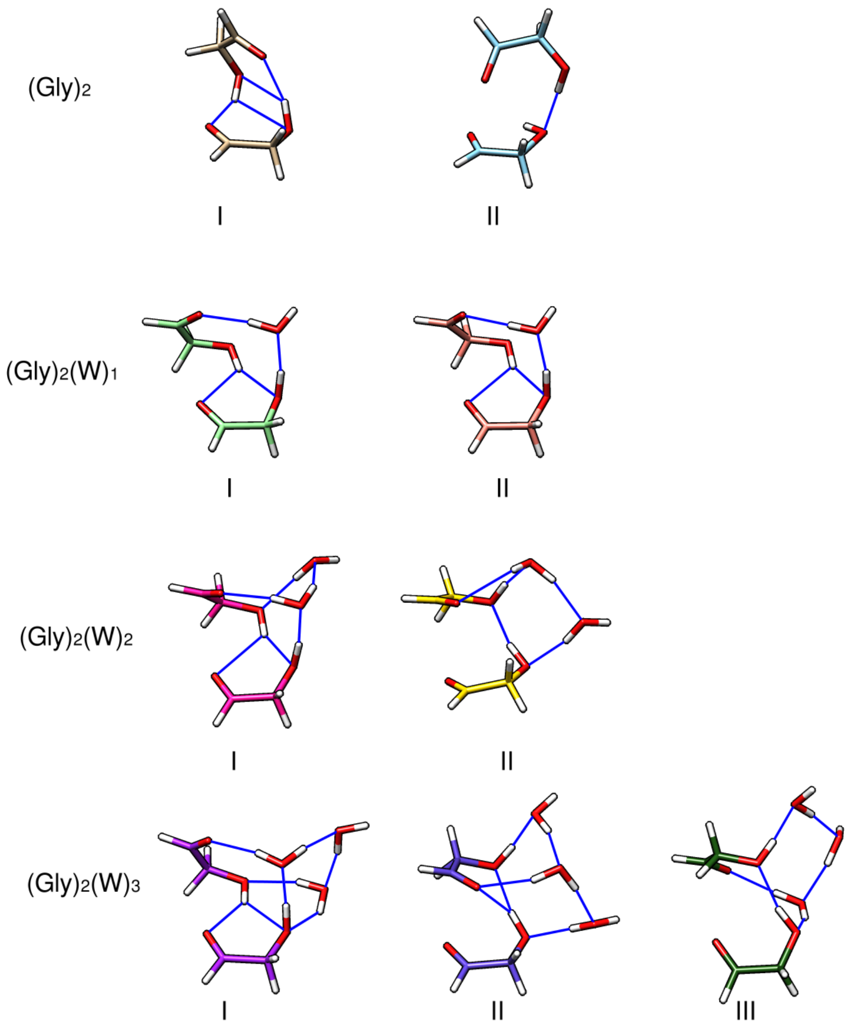


Figure S19: The structures of the low energy $(Gly)_2W_{n=0-3}$ isomers. See the tables below for their Cartesian coordinates.

Table S32: MP2/aVDZ optimized Cartesian coordinates[a] of glycoaldehyde (Gly) and water monomers

| | | | |
|---|---|---|---|
| 8 | | | |
| Gly | | | |
| O | -1.33943 | -0.5912 | 0.00000 |
| O | 1.36278 | -0.60735 | 0.00000 |
| C | -0.67449 | 0.65542 | 0.00000 |
| C | 0.82857 | 0.49747 | 0.00000 |
| H | -0.95403 | 1.25093 | 0.89049 |
| H | -0.95403 | 1.25093 | -0.89049 |
| H | 1.43128 | 1.43287 | 0.00000 |
| H | -0.63448 | -1.26371 | 0.00000 |
| 3 | | | |
| Water | | | |
| O | 0.00000 | 0.11911 | 0.00000 |
| H | 0.76050 | -0.47642 | 0.00000 |
| H | -0.76050 | -0.47642 | 0.00000 |

[a] In Angstroms

#### 2.8.2 $(Gly)_2$

Table S33: MP2/aVDZ optimized Cartesian coordinates[a] of $(Gly)_2W_0$-I

| | | | |
|---|---|---|---|
| 16 | | | |
| $Gly_2W_0$-I (global minimum) | | | |
| C | -2.123478 | 0.554634 | 0.051819 |
| H | -3.179475 | 0.418852 | 0.345675 |
| H | -2.090687 | 1.441472 | -0.615001 |
| O | -0.778261 | -1.371473 | -0.575700 |
| O | -1.358761 | 0.712272 | 1.221965 |
| H | -0.460906 | 0.963668 | 0.934839 |
| C | -1.735489 | -0.635340 | -0.798244 |
| H | -2.385755 | -0.812701 | -1.687359 |
| C | 2.123470 | -0.554627 | 0.051822 |
| H | 3.179466 | -0.418836 | 0.345673 |
| H | 2.090688 | -1.441465 | -0.614999 |
| O | 0.778269 | 1.371448 | -0.575719 |
| O | 1.358768 | -0.712284 | 1.221977 |
| H | 0.460913 | -0.963681 | 0.934859 |
| C | 1.735483 | 0.635340 | -0.798250 |
| H | 2.385755 | 0.812721 | -1.687355 |

[a] In Angstroms

Table S34: MP2/aVDZ optimized Cartesian coordinates[a] of $(Gly)_2W_0$-II

| | | | |
|---|---|---|---|
| 16 | | | |
| $Gly_2$-II | | | |
| O | -1.143701 | 0.834070 | -0.202867 |
| O | 0.493629 | 2.848773 | -0.775138 |
| C | -0.067119 | 0.548993 | -1.094371 |
| C | 0.816433 | 1.765288 | -1.253395 |
| H | -0.451868 | 0.265399 | -2.092340 |
| H | 0.535195 | -0.282224 | -0.696770 |
| H | 1.752269 | 1.641514 | -1.837471 |
| H | -1.116043 | 1.804023 | -0.084523 |
| O | -0.413050 | -0.644072 | 2.058923 |
| O | 1.895775 | 0.752440 | 1.180787 |
| C | 0.327925 | 0.157438 | 2.944712 |
| C | 1.573196 | 0.795210 | 2.362187 |
| H | 0.649480 | -0.475833 | 3.790642 |
| H | -0.266211 | 0.985889 | 3.388962 |
| H | 2.200298 | 1.346964 | 3.104494 |
| H | -0.720268 | -0.083271 | 1.316695 |

[a] In Angstroms

#### 2.8.3 $(Gly)_2W_1$

Table S35: MP2/aVDZ optimized Cartesian coordinates[a] of $(Gly)_2W_1$-I

| | | | |
|---|---|---|---|
| 19 | | | |
| $Gly_2W_1$-I (global minimum) | | | |
| O | 1.11004 | 0.13237 | 1.5341 |
| O | 1.72301 | 0.54261 | -1.2143 |
| C | 1.95611 | -0.75254 | 0.8432 |
| C | 2.19123 | -0.41925 | -0.6126 |
| H | 1.61216 | -1.80424 | 0.8736 |
| H | 2.94822 | -0.72866 | 1.3319 |
| H | 2.86666 | -1.13002 | -1.1446 |
| H | 0.18760 | -0.04336 | 1.2630 |
| O | -1.69508 | 0.51105 | 0.9867 |
| O | -0.52629 | -1.50250 | -0.5475 |
| C | -2.48396 | -0.20698 | 0.0645 |
| C | -1.72237 | -1.27656 | -0.6919 |
| H | -2.96541 | 0.44862 | -0.6882 |
| H | -3.30140 | -0.71437 | 0.6094 |
| H | -2.33364 | -1.87189 | -1.4118 |
| H | -1.24955 | 1.23718 | 0.4854 |
| O | -0.34301 | 2.28431 | -0.5338 |
| H | 0.45935 | 1.73824 | -0.6864 |
| H | -0.01947 | 3.05775 | -0.0538 |

[a] In Angstroms

Table S36: MP2/aVDZ optimized Cartesian coordinates[a] of $(Gly)_2W_1$-II

| | | | |
|---|---|---|---|
| 19 | | | |
| $Gly_2W_1$-II | | | |
| O | 0.752008 | 0.109086 | -1.34667 |
| O | 1.270316 | -0.229684 | 1.42935 |
| C | 1.731013 | -0.795218 | -0.894609 |
| C | 1.893738 | -0.891767 | 0.60675 |
| H | 2.740815 | -0.578906 | -1.30433 |
| H | 1.457188 | -1.80313 | -1.25396 |
| H | 2.669414 | -1.62148 | 0.939737 |
| H | 0.917260 | 0.999232 | -0.966867 |
| O | -1.316637 | 0.876487 | 0.929094 |
| O | -1.079823 | -1.78753 | 0.195351 |
| C | -2.023712 | 0.400328 | -0.206547 |
| C | -1.794476 | -1.06567 | -0.489542 |
| H | -1.744038 | 0.950006 | -1.12601 |
| H | -3.105192 | 0.555423 | -0.042686 |
| H | -2.328335 | -1.45942 | -1.38561 |
| H | -0.557403 | 0.274112 | 1.07759 |
| O | 0.464776 | 2.62818 | -0.255037 |
| H | -0.200862 | 2.20186 | 0.32865 |
| H | 1.015842 | 3.15279 | 0.338826 |

[a] In Angstroms

Table S37: MP2/aVDZ optimized Cartesian coordinates[a] of $(Gly)_2W_1$-III

| | | | |
|---|---|---|---|
| 19 | | | |
| $Gly_2W_1$-III | | | |
| O | -0.825268 | -1.09855 | 0.776881 |
| O | -2.806376 | 0.657691 | 0.003251 |
| C | -1.409884 | -1.26479 | -0.504095 |
| C | -2.514452 | -0.262459 | -0.750226 |
| H | -1.850237 | -2.27491 | -0.619526 |
| H | -0.671967 | -1.12528 | -1.31635 |
| H | -3.069474 | -0.419348 | -1.70631 |
| H | 0.141941 | -1.2064 | 0.669957 |
| O | 0.941924 | 0.871218 | -1.04848 |
| O | 1.957172 | -1.23253 | 0.56406 |
| C | 2.312500 | 0.795843 | -0.747747 |
| C | 2.721716 | -0.395401 | 0.092432 |
| H | 2.694412 | 1.69609 | -0.222744 |
| H | 2.881976 | 0.725925 | -1.69318 |
| H | 3.819104 | -0.482318 | 0.272934 |
| H | 0.483410 | 1.21189 | -0.243485 |
| O | -0.270222 | 1.58091 | 1.28561 |
| H | -0.734465 | 0.715677 | 1.28886 |
| H | -0.995150 | 2.21628 | 1.21086 |

[a] In Angstroms

### 2.8.4 $(Gly)_2W_2$

Table S38: MP2/aVDZ optimized Cartesian coordinates[a] of $(Gly)_2W_2$-I

| | | | |
|---|---|---|---|
| 22 | | | |
| $Gly_2W_2$-I (global minimum) | | | |
| O | -0.93127 | -1.43745 | -0.72408 |
| O | -1.94621 | 1.10031 | -0.23153 |
| C | -2.21429 | -1.30936 | -0.15130 |
| C | -2.64521 | 0.13013 | 0.03807 |
| H | -2.28750 | -1.80899 | 0.83451 |
| H | -2.96903 | -1.78855 | -0.80409 |
| H | -3.67566 | 0.26377 | 0.44557 |
| H | -0.29921 | -1.67424 | 0.00775 |
| O | 0.92949 | 0.55678 | -1.22529 |
| O | 0.59990 | 1.05552 | 1.56505 |
| C | 0.84422 | 1.87321 | -0.72354 |
| C | 0.65691 | 1.98955 | 0.77374 |
| H | 0.02315 | 2.44836 | -1.19035 |
| H | 1.78732 | 2.39267 | -0.97235 |
| H | 0.57067 | 3.04068 | 1.13821 |
| H | 0.07685 | 0.09142 | -1.06490 |
| O | 2.92197 | -1.21495 | -0.44955 |
| H | 2.29696 | -0.56476 | -0.83754 |
| H | 3.29498 | -1.68614 | -1.20455 |
| O | 0.78985 | -1.81719 | 1.27617 |
| H | 1.63506 | -1.87694 | 0.78552 |
| H | 0.78684 | -0.88258 | 1.55412 |

[a] In Angstroms

Table S39: MP2/aVDZ optimized Cartesian coordinates[a] of $(Gly)_2W_2$-II

| | | | |
|---|---|---|---|
| 22 | | | |
| $Gly_2W_2$-II | | | |
| O | -0.63315 | 1.54915 | -0.75920 |
| O | 2.13124 | 1.28138 | -0.40928 |
| C | -0.02847 | 1.95819 | 0.45369 |
| C | 1.47732 | 1.82897 | 0.47035 |
| H | -0.39597 | 1.38350 | 1.33007 |
| H | -0.28896 | 3.01609 | 0.62841 |
| H | 1.97114 | 2.25005 | 1.37811 |
| H | -0.25406 | 0.66881 | -1.00731 |
| O | 0.00238 | -1.04640 | -1.27052 |
| O | 0.81318 | -1.10390 | 1.42178 |
| C | 1.25101 | -1.60346 | -0.91479 |
| C | 1.58577 | -1.50671 | 0.55682 |
| H | 2.03910 | -1.06849 | -1.46971 |
| H | 1.32413 | -2.67611 | -1.19035 |
| H | 2.61180 | -1.85113 | 0.82554 |
| H | -0.70258 | -1.43767 | -0.69083 |
| O | -1.95157 | -1.82503 | 0.43526 |
| H | -2.51497 | -1.02428 | 0.35409 |
| H | -1.43603 | -1.66104 | 1.23932 |
| O | -3.10004 | 0.68677 | 0.08608 |
| H | -2.31506 | 1.09588 | -0.34502 |
| H | -3.84858 | 0.90676 | -0.48164 |

[a] In Angstroms

### 2.8.5 $(Gly)_2W_3$

Table S40: MP2/aVDZ optimized Cartesian coordinates[a] of $(Gly)_2W_3$-I

| | | | |
|---|---|---|---|
| 25 | | | |
| $Gly_2W_3$-I (global minimum) | | | |
| O | -0.948336 | -1.718152 | -0.632504 |
| O | -1.959653 | 0.804150 | -0.115852 |
| C | -2.236802 | -1.602991 | -0.076996 |
| C | -2.662046 | -0.166763 | 0.150517 |
| H | -2.332213 | -2.131583 | 0.892820 |
| H | -2.973939 | -2.060513 | -0.761950 |
| H | -3.678728 | -0.029167 | 0.586958 |
| H | -0.292479 | -1.583924 | 0.115324 |
| O | 0.578003 | 0.674862 | -1.482581 |
| O | 0.861369 | 1.594501 | 1.179574 |
| C | 0.560498 | 2.054485 | -1.196540 |
| C | 0.733259 | 2.402070 | 0.266883 |
| H | -0.374074 | 2.547219 | -1.531066 |
| H | 1.390136 | 2.531129 | -1.747764 |
| H | 0.732380 | 3.497230 | 0.481306 |
| H | -0.219626 | 0.282653 | -1.074741 |
| O | 2.895238 | -2.316902 | -0.086984 |
| H | 3.333105 | -3.175379 | -0.050579 |
| H | 2.435248 | -2.291783 | -0.958201 |
| O | 0.751612 | -1.278090 | 1.317529 |
| H | 1.587109 | -1.675751 | 0.987373 |
| H | 0.905200 | -0.315010 | 1.280522 |
| O | 1.368506 | -1.935636 | -2.352411 |
| H | 1.345708 | -0.962418 | -2.317507 |
| H | 0.471208 | -2.158910 | -2.050756 |

[a] In Angstroms

Table S41: MP2/aVDZ optimized Cartesian coordinates[a] of $(Gly)_2W_3$-II

| | | | |
|---|---|---|---|
| 25 | | | |
| $Gly_2W_3$-II | | | |
| O | -0.515794 | 1.407747 | 1.274978 |
| O | -0.690300 | -1.092895 | 2.561740 |
| C | -0.903566 | 1.331253 | 2.632142 |
| C | -0.985805 | -0.075499 | 3.181215 |
| H | -1.906700 | 1.776881 | 2.736694 |
| H | -0.217055 | 1.897419 | 3.292245 |
| H | -1.349753 | -0.141682 | 4.233236 |
| H | 0.456511 | 1.228836 | 1.241964 |
| O | -1.797022 | -0.101074 | -0.603984 |
| O | -3.520491 | 0.195314 | 1.592141 |
| C | -2.498322 | -1.216763 | -0.089987 |
| C | -3.450711 | -0.894724 | 1.037169 |
| H | -1.822260 | -2.009461 | 0.294282 |
| H | -3.078231 | -1.665433 | -0.914819 |
| H | -4.088855 | -1.749832 | 1.364482 |
| H | -1.425646 | 0.427802 | 0.146122 |
| O | 0.247723 | -1.471444 | -1.781792 |
| H | 0.641902 | -1.085179 | -2.572956 |
| H | -0.458758 | -0.843617 | -1.505778 |
| O | 1.513678 | -1.767117 | 0.706218 |
| H | 1.150761 | -1.738513 | -0.204315 |
| H | 0.725103 | -1.777489 | 1.273753 |
| O | 2.076247 | 0.740880 | 1.482129 |
| H | 2.844002 | 1.147581 | 1.063054 |
| H | 2.040183 | -0.191734 | 1.144422 |

[a] In Angstroms

Table S42: MP2/aVDZ optimized Cartesian coordinates[a] of $(Gly)_2W_3$-III

| | | | |
|---|---|---|---|
| 25 | | | |
| $Gly_2W_3$-III | | | |
| O | 0.571475 | 1.436254 | -0.538859 |
| O | 0.760593 | -1.374231 | -0.075784 |
| C | 1.035772 | 0.558446 | -1.542105 |
| C | 1.029787 | -0.910106 | -1.176407 |
| H | 2.073019 | 0.799904 | -1.852144 |
| H | 0.397611 | 0.671036 | -2.433678 |
| H | 1.326298 | -1.585028 | -2.014534 |
| H | 1.259106 | 1.476016 | 0.174701 |
| O | -1.879712 | 1.425069 | 0.629598 |
| O | -1.848844 | -0.224218 | -1.637807 |
| C | -3.033475 | 1.291548 | -0.167081 |
| C | -2.901536 | 0.313304 | -1.313561 |
| H | -3.351606 | 2.256891 | -0.616272 |
| H | -3.866938 | 0.955847 | 0.474925 |
| H | -3.843331 | 0.125223 | -1.882288 |
| H | -1.070537 | 1.412756 | 0.060938 |
| O | -0.678297 | -0.533282 | 2.266883 |
| H | -1.247302 | 0.153324 | 1.867683 |
| H | -0.210966 | -0.885073 | 1.486817 |
| O | 1.435533 | 0.533976 | 3.561353 |
| H | 0.612195 | 0.129036 | 3.184224 |
| H | 1.828027 | -0.158903 | 4.106184 |
| O | 2.538149 | 1.493932 | 1.286533 |
| H | 2.225494 | 1.124346 | 2.150742 |
| H | 2.860207 | 2.377775 | 1.504709 |

[a] In Angstroms

# References


(S1) Plusquellic, D. F.; Suenram, R. D.; Mat, B.; Jensen, J. O.; Samuels, A. C. *The Journal of Chemical Physics* **2001**, *115*, 3057–3067.

(S2) Kisiel, Z.; Pszczółkowski, L.; Medvedev, I. R.; Winnewisser, M.; Lucia, F. C. D.; Herbst, E. *Journal of Molecular Spectroscopy* **2005**, *233*, 231 – 243.

(S3) Western, C. M. *Journal of Quantitative Spectroscopy and Radiative Transfer* **2017**, *186*, 221 – 242, Satellite Remote Sensing and Spectroscopy: Joint ACE-Odin Meeting, October 2015.

(S4) Goldberg, D. E. *Genetic Algorithms in Search, Optimization and Machine Learning*; Addison-Wesley, 1989.

(S5) Dieterich, J. M.; Hartke, B. *Molecular Physics* **2009**, *108*, 279–291.

(S6) Stewart, J. P. *Journal of Molecular Modeling* **2013**, *19*, 1–32.

(S7) Stewart, J. J. P. MOPAC2009 Computational Chemistry, http://OpenMOPAC.net (2008). 2008.

(S8) Sure, R.; Grimme, S. *Journal of Computational Chemistry* **2013**, *34*, 1672–1685.

(S9) Neese, F. ORCA 4.2.1 - An ab initio, DFT and semiempirical SCF-MO package. Max-Planck-Institut fur Kohlenforschunmg, Ruhr, Germany. 2019. See https://orcaforum.kofo.mpg.de. 2019.

(S10) Kanters, R. P. F.; Donald, K. J. *Journal of Chemical Theory and Computation* **2014**, *10*, 5729–5737.

(S11) Kaliman, I. A.; Slipchenko, L. V. *Journal of Computational Chemistry* **2013**, *34*, 2284–2292.

(S12) Møller, C.; Plesset, M. S. *Physical Review* **1934**, *46*, 618–622.

(S13) Dunning, T. H. *Journal of Chemical Physics* **1989**, *90*, 1007–1023.

(S14) Frisch, M. J.; Trucks, G. W.; Schlegel, H. B.; Scuseria, G. E.; Robb, M. A.; Cheeseman, J. R.; Scalmani, G.; Barone, V.; Petersson, G. A.; Nakatsuji, H.; Li, X.; Caricato, M.; Marenich, A. V.; Bloino, J.; Janesko, B. G.; Gomperts, R.; Mennucci, B.; Hratchian, H. P.; Ortiz, J. V.; Izmaylov, A. F.; Sonnenberg, J. L.; Williams-Young, D.; Ding, F.; Lipparini, F.; Egidi, F.; Goings, J.; Peng, B.; Petrone, A.; Henderson, T.; Ranasinghe, D.; Zakrzewski, V. G.; Gao, J.; Rega, N.; Zheng, G.; Liang, W.; Hada, M.; Ehara, M.; Toyota, K.; Fukuda, R.; Hasegawa, J.; Ishida, M.; Nakajima, T.; Honda, Y.; Kitao, O.; Nakai, H.; Vreven, T.; Throssell, K.; Montgomery, J. A., Jr.; Peralta, J. E.; Ogliaro, F.; Bearpark, M. J.; Heyd, J. J.; Brothers, E. N.; Kudin, K. N.; Staroverov, V. N.; Keith, T. A.; Kobayashi, R.; Normand, J.; Raghavachari, K.; Rendell, A. P.; Burant, J. C.; Iyengar, S. S.; Tomasi, J.; Cossi, M.; Millam, J. M.; Klene, M.; Adamo, C.; Cammi, R.; Ochterski, J. W.; Martin, R. L.; Morokuma, K.; Farkas, O.; Foresman, J. B.; Fox, D. J. Gaussian16 Revision B.01. 2016; Gaussian Inc. Wallingford CT.

(S15) Neese, F. *Wiley Interdisciplinary Reviews: Computational Molecular Science* **2012**, *2*, 73–78.

(S16) Grimme, S.; Ehrlich, S.; Goerigk, L. *Journal of Computational Chemistry* **2011**, *32*, 1456–1465.

(S17) Zinn, S.; Medcraft, C.; Betz, T.; Schnell, M. *Angewandte Chemie International Edition* **2016**, *55*, 5975–5980.

(S18) Richard, R. M.; Herbert, J. M. *The Journal of Chemical Physics* **2012**, *137*, 064113.

(S19) Hodges, M. P.; Stone, A. J.; Xantheas, S. S. *Journal of Physical Chemistry A* **1997**, *101*, 9163–9168.

(S20) Riera, M. Many-Body Decomposition. `https://github.com/chemphys/ManyBodyDecomposition`, 2019.

(S21) Senent, M. L. *The Journal of Physical Chemistry A* **2004**, *108*, 6286–6293.

(S22) Mills, J.; Dean, P. *Journal of Computer-Aided Molecular Design* **1996**, *10*, 607–622.

(S23) Pettersen, E. F.; Goddard, T. D.; Huang, C. C.; Couch, G. S.; Greenblatt, D. M.; Meng, E. C.; Ferrin, T. E. *Journal of Computational Chemistry* **2004**, *25*, 1605–1612.

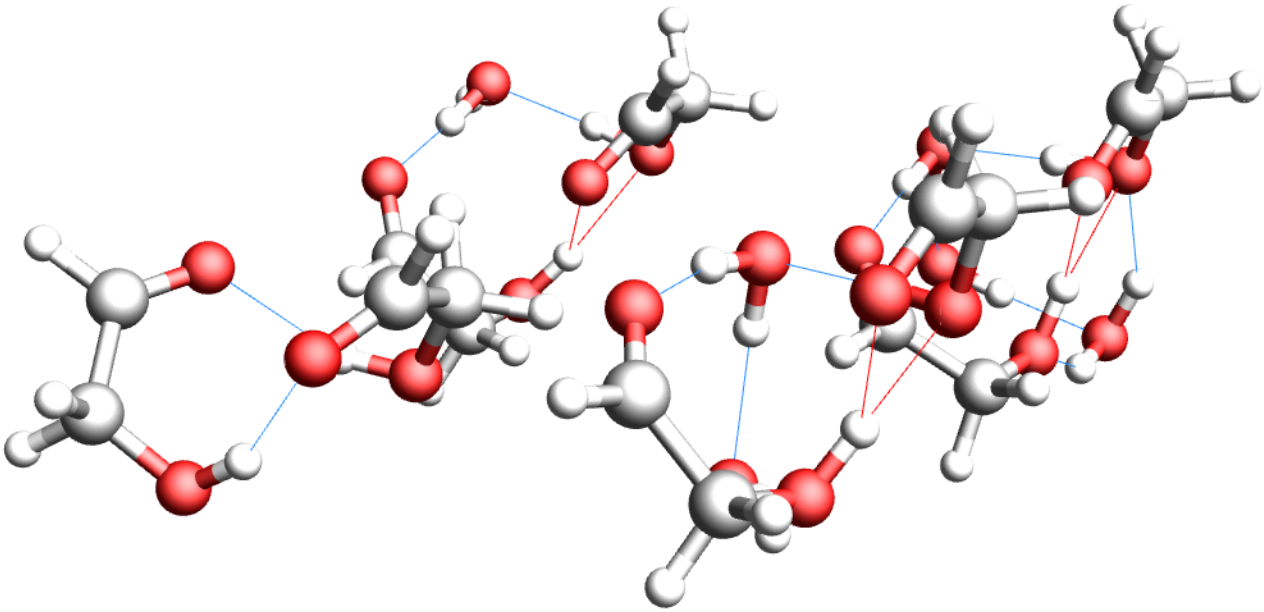

Supplement: Supplementary file 1 — Supplementary [file ANIE-59-8401-s001.pdf]
